# TBX20 promotes doxorubicin resistance in breast cancer cells through enhanced ABCC1 expression and inhibition of mitophagy

**DOI:** 10.1371/journal.pone.0353376

**Published:** 2026-07-17

**Authors:** Shuai Nie, Dejian Liu, Jun Hu, Yu Gan, Liangliang Mi

**Affiliations:** Physician of general surgery, Hunan Aerospace Hospital Affiliated to Hunan Normal University, Changsha, China; Longgang Otorhinolaryngology Hospital & Shenzhen Key Laboratory of Otorhinolaryngology, Shenzhen Institute of Otorhinolaryngology, CHINA

## Abstract

This study aimed to investigate the role and mechanism of T-box transcription factor 20 (TBX20) in doxorubicin resistance in breast cancer cells. RNA-seq data from breast cancer samples in the TCGA database were analyzed. Lentiviral vectors were used to establish TBX20 overexpression and silencing models in MCF-7 and MDA-MB-231 cells. Gene and protein expression were detected by qPCR and Western blot, respectively. Cell viability and the half-maximal inhibitory concentration of doxorubicin were measured using the CCK-8 assay. Apoptosis, migration, and invasion were analyzed by flow cytometry, wound healing assay, and Transwell assay. Mitophagy levels were assessed via immunofluorescence staining and western blotting. ChIP and dual-luciferase reporter assays were performed to validate the transcriptional regulation of ABCC1 by TBX20. Results showed that TCGA data analysis revealed a high expression of TBX20 in breast cancer tissues, which was positively correlated with ABCC1 expression. In MCF-7 and MDA-MB-231 cells, TBX20 overexpression significantly enhanced cell proliferation, migration, invasion, and resistance to doxorubicin, while suppressing the expression of mitophagy-related proteins LC3-II/LC3-I, PINK1, and BNIP3. ChIP and dual-luciferase reporter assays confirmed that TBX20 directly binds to and activates the ABCC1 promoter. Silencing of ABCC1 or restoration of mitophagy by CCCP reversed TBX20 overexpression‑induced doxorubicin resistance. TBX20 enhances the resistance of breast cancer cells to doxorubicin by transcriptionally upregulating ABCC1 and is correlated with the suppression of mitophagy.

## Introduction

Breast Cancer is one of the most common malignancies among women worldwide, with high incidence and mortality rates making it a leading cause of female cancer deaths. This underscores the critical need for innovative therapeutic approaches in breast cancer treatment [1,2]. According to data from GLOBOCAN 2020, there were over 2.3 million new cases of breast cancer and 685,000 deaths [3]. Treatment strategies for breast cancer primarily include surgery, chemotherapy, radiotherapy, hormonal therapy, and targeted therapy [4–6]. However, one of the primary challenges in treatment is tumor recurrence and metastasis, particularly due to the emergence of Multidrug Resistance (MDR), which diminishes the efficacy of many chemotherapeutic agents. Reports suggest that over 90% of chemotherapy-related deaths are associated with MDR, highlighting the urgent need for novel treatment modalities [7]. In the context of breast cancer therapy, Doxorubicin (Adriamycin, ADR) is a commonly used chemotherapeutic agent whose effectiveness is often limited by resistance, constraining its clinical utility [3].

T-box transcription factor 20 (TBX20) is part of the T-box transcription factor family, playing a critical role in embryonic development and is closely associated with various disorders, including heart defects, intellectual disabilities, and cancer [8,9]. Yet, the biological functions, clinical significance, and molecular mechanisms of TBX20 in breast cancer remain unreported. Mitophagy, the selective encapsulation and degradation of damaged mitochondria through the autophagy mechanism, is essential for maintaining mitochondrial and intracellular homeostasis [10]. To date, no studies have investigated the impact of TBX20 on mitochondrial autophagy in cells. ATP binding cassette subfamily C member 1 (ABCC1), also known as Multidrug Resistance Protein 1 (MRP1), plays a crucial role in maintaining the balance of the intracellular and extracellular environment [11].

This study aims to investigate the role of the transcription factor TBX20 in breast cancer, particularly focusing on its contribution to ADR resistance. We will explore the underlying mechanisms, specifically examining whether TBX20 promotes chemoresistance through the ABCC1 and/or via the modulation of mitochondrial autophagy. This research is designed to provide foundational insights into the complex mechanisms of ADR resistance in breast cancer and to assess the therapeutic potential of targeting the TBX20 pathway. The findings may contribute to the development of novel strategies to overcome chemoresistance and improve clinical outcomes for breast cancer patients.

## Materials and methods

### TCGA database analysis

RNA-seq data from the TCGA-BRCA (Breast Cancer) project were downloaded from the TCGA database (https://portal.gdc.cancer.gov). TPM formatted data were extracted and subsequently processed using R software (version 4.2.1) employing the log_2_(value+1) method. Based on the data characteristics, visualizations were generated using the ggplot2 package (version 3.3.6).

### Breast cancer cell culture

Breast cancer cell lines MCF-7 and MDA-MB-231 were purchased from Wuhan Pricella Biotechnology Co., Ltd. Cells were cultured in DMEM medium (Hyclone, SH30022.01B) supplemented with 10% (v/v) fetal bovine serum (Gibco, 42F7180K) and 1% penicillin-streptomycin (Beyotime, ST488S). Cultures were maintained in a cell incubator (Thermo Fisher, 3111) at 37℃ with 95% relative humidity and 5% CO_2_. The medium was replaced every two days until the desired cell density was achieved for experiments.

### Vector construction and transduction

The TBX20 gene coding sequence (GenBank ID: NM_001077653.2) was synthesized and subsequently verified by sequencing (Generalbiol, Anhui, China). It was then cloned into the GV358 lentiviral overexpression vector (Genechem, Shanghai, China) using BamHI and AgeI restriction sites. The restriction digestion was performed at 37°C for 2 h, followed by ligation at 4°C overnight. Lentiviral particles were produced using a third-generation packaging system (Genechem). Briefly, 293T cells were co-transfected with the overexpression plasmid, along with the packaging plasmids psPAX2 and pMD2.G, at a mass ratio of 4:3:1 using Lipofectamine 3000 (Invitrogen, L3000015). The viral supernatant was harvested 48 h post-transfection, concentrated, and titrated. For transduction, MCF-7 and MDA-MB-231 cells were incubated with the lentiviral particles at a multiplicity of infection (MOI) of 10 in the presence of 5 μg/mL polybrene (Genechem) for 24 h, after which the medium was replaced with fresh complete medium. Transduction efficiency was assessed 96 h later by quantifying TBX20 mRNA and protein levels via qPCR and Western blot analysis, respectively. For silencing experiments, small interfering RNAs (siRNAs) targeting TBX20 (si-TBX20#1 sequence: AACAAUGAACUGGAUCAACAU; si-TBX20#2 sequence: UGGAUCAACAUGGCCAUAUAA) and a negative control siRNA (NC sequence: ACGUGACUCGUUCGGAGAA) were synthesized (Huzhou Hippo Biotechnology Co., Ltd.).

Similarly, ABCC1 gene (GenBank ID: NM_004996.4) siRNA and a negative control were synthesized by Huzhou Hippo Biotechnology Co., Ltd. The siRNA (si-ABCC1#1 sequence: GGCCUGUUUCCCCUUCUAC; si-ABCC1#2 sequence: UACUCUUUCUGGGAAAGAAGU; NC sequence: ACGUGACUCGUUCGGAGAA) was transfected using Lipofectamine 3000 (Invitrogen, L3000015) in a 1:3 ratio into MCF-7 and MDA-MB-231 cells. After 12 h, the medium was replaced with regular medium, and cells were analyzed after 48 h for mRNA and protein levels of ABCC1 using Real-time PCR and Western blot.

### Real-time quantitative PCR (qPCR)

Samples were ground in liquid nitrogen, and RNA was extracted using Trizol reagent (TAKARA, 9109) following the manufacturer’s instructions. RNA concentration was measured using a Nanodrop spectrophotometer (Q6000UV, Quawell Technology). cDNA synthesis was performed using the BestarTM qPCR RT kit (DBI, 2220), which includes a gDNA Remover and RT Enzyme Mix. Real-time PCR amplifications were conducted using the Stratagene Real-time PCR system (Mx3000P, Agilent) with DBI’s BestarTM qPCR MasterMix (2043) and specific primers for GAPDH, TBX20, and ABCC1. Relative expression levels were calculated using the 2^-△△Ct^ method. Primer sequences were as follows: GAPDH forward 5’-TGTTCGTCATGGGTGTGAAC-3’, reverse 5’-ATGGCATGGACTGTGGTCAT-3’; TBX20 forward 5’-CAACCCCAAATCGAGGGTCA-3’, reverse 5’-GCTATGGATGCTGTGCTGGT-3’; ABCC1 forward 5’-CCCGCTCTGGGACTGGAA-3’, reverse 5’-GTAGAAGGGGAAACAGGCCC-3’.

### Western blot analysis

Cells were washed with PBS and lysed in 200 µL of RIPA lysis buffer (Beyotime, P0013B) per well. The lysates were incubated at 4℃ for 20 min and centrifuged to collect the supernatant. Protein concentrations were determined using the BCA Protein Assay Kit (Beyotime, P0012) by plotting a standard curve. For SDS-PAGE, separation gels and stacking gels were prepared, and 20 µg of protein along with a marker (Servicebio, G2086) were loaded per lane. Electrophoresis was conducted at a constant voltage of 100 V until samples entered the separation gel, then voltage was increased to 120 V until the bromophenol blue reached the bottom of the gel. Proteins were transferred to a PVDF membrane (Beyotime, FFP26), and incubated with primary and secondary antibodies. Primary antibodies included anti-TBX20 (Abcam, ab197386, 1:500), anti-LC3 (Abcam, ab192890, 1:2000), anti-P62 (Abcam, ab109012, 1:10000), anti-PINK1 (Abcam, ab216144, 1:1000), anti-BNIP3 (Abcam, ab109362, 1:1000), anti-ABCC1 (the protein name of ABCC1 is MRP1) (Abcam, ab265256, 1:500), and anti-GAPDH (Abcam, ab8245, 1:1000). The secondary antibody was HRP-conjugated Goat anti-Rabbit IgG (Abcam, ab3368, 1:10000). The membrane was developed using ECL (Beyotime, P0018), and bands were visualized and quantified using ImageJ software (National Institutes of Health, version 1.8.0).

### CCK8 assay

To assess the effect of mitophagy induction on doxorubicin sensitivity, cells were first pretreated with or without the mitophagy inducer carbonyl cyanide m-chlorophenyl hydrazone (CCCP) (10 μM, Sigma-Aldrich, C2759) for 24 h. After pretreatment, the medium was replaced with fresh medium containing gradient concentrations of doxorubicin (Sigma, D1515). For MCF-7 cells, doxorubicin concentrations were set at 0, 0.5, 1.5, 2, 4, 8, and 16 µM; for MDA-MB-231 cells, concentrations were 0, 0.25, 0.5, 1.5, 2, 4, and 8 µM. Cells were then incubated for an additional 24 h. Subsequently, a CCK-8 assay was conducted by adding 1/10 volume of CCK-8 solution (TransGen Biotech, FC101−03) to each well, followed by incubation for 3 h. The absorbance at 450 nm (OD450) was measured using a microplate reader (BioTek, ELx800).

The concentration-response data were analyzed by normalizing the absorbance values to the untreated control (0 μM doxorubicin). For curve fitting and IC50 determination, doxorubicin concentrations were log-transformed (X = log_10_(X)). The half-maximal inhibitory concentration (IC_50_) values were calculated using a four-parameter logistic nonlinear regression model in Prism software (version 9.0, GraphPad Software, Inc), where the IC_50_ represents the midpoint of the sigmoidal curve. For cell proliferation assays following genetic manipulation (e.g., transduction), CCK-8 solution was added daily to the cells for 5 consecutive days, and OD450 readings were used to plot cell proliferation curves over time.

### Wound healing assay

A uniform line was marked on the underside of a 6-well plate at 1 cm intervals using a marker. Cells grown to 80% confluence were washed three times with PBS (absin, abs961), treated with trypsin solution (Beyotime, C0201) to detach, and once cells appeared spherical, fresh complete medium was added to prepare a single-cell suspension. Cell density was adjusted to 5 × 10^5^ cells/mL, and 1 mL of this suspension was seeded into each well of a 6-well plate containing drug-supplemented complete medium and incubated at 37℃ with 5% CO_2_ for 48 h. After incubation, a pipette tip was used to scratch vertically across the marked lines, and images were taken. Cells were washed three times with PBS to remove detached cells and cultured in serum-free medium for an additional 24 h at 37℃ with 5% CO_2_. The migration was observed and photographed using an inverted microscope (Nikon, DS-Fi3) and analyzed using Image-Pro Plus software (MEDIA CYBERNETICS, version 6.0).

### Transwell assay

Matrigel matrix gel (Corning, 356234) was thawed overnight at 4℃. The gel was mixed with precooled serum-free medium at a 1:15 ratio to prepare the gel solution for the Transwell upper chamber and allowed to solidify at 37℃ for 2 h. Excess liquid was removed from the solidified Matrigel in the Transwell inserts (Corning, 3422), and residual non-solidified Matrigel was washed away with sterile PBS. Before seeding, cells were starved in serum-free medium for 12 h. Cells were then digested with trypsin, resuspended, and the density adjusted to 2 × 10^5^ cells/mL. 100 µL of the cell suspension was added to the upper chamber of the Transwell, and 600 µL of complete medium containing 10% FBS was added to the lower chamber. After 24 h of incubation, the inserts were removed, washed three times with PBS, stained with crystal violet (Solarbio Life Science, G1062) for 10 min, rinsed with running water, and cells in three random fields were counted.

### Immunofluorescence assay

Cells were cultured to 80% confluence and seeded onto sterile glass coverslips in 24-well plates. After 24 h of culture, cells were washed three times with PBS and fixed with 4% paraformaldehyde (Beyotime, P0099) for 15 min at room temperature. Following fixation, cells were washed three times with PBS and permeabilized with 0.5% Triton X-100 (Beyotime, P0096) in PBS for 20 min at room temperature. Nonspecific binding sites were blocked by incubating cells with 5% BSA in PBS for 1 h at room temperature. Subsequently, cells were incubated overnight at 4°C with primary antibodies against LC3 (Abcam, ab192890, 1:300, Rabbit) and TOM20 (Santa Cruz, sc-17764, 1:200, Mouse). The next day, after washing three times with PBST (PBS containing 0.1% Tween-20), cells were incubated with species-specific fluorescently labeled secondary antibodies: FITC-conjugated Goat Anti-Rabbit IgG (Servicebio, GB22303, 1:500) and Cy3-conjugated Goat Anti-Mouse IgG (Beyotime, A0521, 1:500) at 37°C for 1 h in the dark. Nuclei were stained with DAPI (1 μg/mL, Beyotime, C1002) for 5 min, and excess DAPI was washed off with PBS. Finally, the slides were mounted with Antifade Mounting Medium (Beyotime, P0126) and examined under a fluorescence microscope (Nikon, DS-Fi3).

### Chromatin immunoprecipitation

MCF-7 cells at the logarithmic growth phase were cross-linked with 1% formaldehyde (Sigma, F8775) at room temperature for 10 min and quenched with 0.125 M glycine for 5 min. Cells were washed twice with PBS and lysed on ice for 10 min in lysis buffer containing 50 mM Tris-HCl, pH 8.0, 10 mM EDTA, 1% SDS, and 1 mM PMSF (Roche, 04693132001). Chromatin was sheared to 200–500 bp fragments using a sonicator (Bioruptor, Diagenode, B01020001) with 30 s on/30 s off cycles for 15 cycles. Lysates were diluted tenfold and incubated overnight at 4℃ with 5 μg anti-TBX20 antibody (Abcam, ab197386) or normal rabbit IgG (Abcam, ab172730), followed by 2 h incubation with pre-blocked Protein A/G magnetic beads (Thermo Fisher, 10003D). Beads were washed sequentially with low-salt, high-salt, LiCl, and TE buffers (5 min each), and the immunocomplexes were reverse cross-linked at 65℃ in 0.2 M NaCl for 4 h. Proteinase K digestion (20 μg, Thermo Fisher, 25530−049) was performed at 55℃ for 1 h, and DNA was extracted with phenol/chloroform/isoamyl alcohol and ethanol precipitated. Purified DNA was used for qPCR analysis of ABCC1 promoter binding.

### Dual luciferase reporter gene experiment

Retrieved from the NCBI database was the promoter sequence of the human ABCC1 gene (2000 bp upstream of the transcription start site). Prediction using the JASPAR database (https://jaspar.elixir.no/) revealed a TBX20 binding site within the ABCC1 promoter region, located 916 bp upstream of the TSS (sequence: GAGGTGGGAAG). A three-site mutation was designed (sequence: GAGCACGGAAG), as illustrated in Fig 8A. MCF-7 cells at logarithmic growth phase were seeded at 5 × 10^4^ cells per well in 24-well plates and cultured in medium supplemented with 10% FBS and 1% penicillin-streptomycin until they reached 70–80% confluence. The cells were transfected with 1 μg of pGL3-ABCC1 wild-type (WT) reporter plasmid or the mutant (MUT) reporter plasmid containing the ABCC1 promoter region(the 2-kb region upstream of the transcription start site, Genechem,Shanghai), along with 1 μg of TBX20 expression plasmid (pcDNA3.1-TBX20, Genechem,Shanghai) or an empty vector as a negative control, using Lipofectamine 3000 transduction reagent (Invitrogen, L3000015) at a 1:3 plasmid-to-reagent ratio. A control plasmid, pRL-TK (Promega, E2241), expressing Renilla luciferase, was co-transfected at 20 ng per well to normalize for transduction efficiency. After 24 h of incubation at 37℃ in 5% CO_2_, cells were lysed with 100 μL of passive lysis buffer (Promega, E1941) at room temperature for 15 min to fully lyse the cells. The lysates were transferred to a white 96-well plate, and firefly luciferase activity was measured using the GloMax 96 Microplate Luminometer (Promega, G9240) following the addition of Luciferase Assay Reagent II (Promega, E1910). Subsequently, Renilla luciferase activity was measured by adding Stop & Glo reagent (Promega, E1910). Each reading was taken after a 10 s delay to allow for signal stabilization. The assay was repeated three times, and firefly luciferase activity was normalized to Renilla luciferase activity to assess the regulatory effect of TBX20 on ABCC1 promoter activity.

### Statistical analysis

Data normality was assessed using the Kolmogorov-Smirnov test to ensure adherence to the prerequisites for statistical analysis. Statistical analyses and graphing were conducted using Prism software (version 9.0, GraphPad Software, Inc), with data presented as mean ± standard deviation (SD). The Wilcoxon rank-sum test was utilized to compare expression differences between clinical samples, Spearman correlation analysis was applied to explore the relationships between variables, and ANOVA was used for comparisons among multiple groups. A significance level of *p* < 0.05 was established to determine statistically significant differences.

## Results

### High expression of TBX20 in breast cancer tissues correlates positively with ABCC1 gene expression

**Fig 1:**
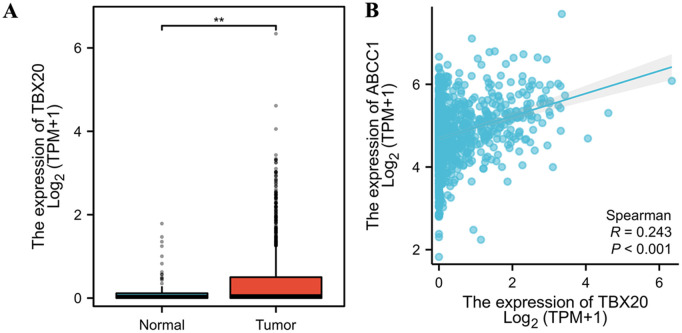
High expression of TBX20 in breast cancer tissues correlates with ABCC1 gene expression. **(A)** TBX20 gene expression levels in breast cancer samples compared with normal samples, analysed using the TCGA database. Data are shown as log_2_(TPM + 1) values. Statistical significance was determined using the Wilcoxon rank-sum test. **(B)** Correlation analysis between TBX20 and ABCC1 gene expressions in breast cancer samples from the TCGA database, assessed using Spearman correlation. Spearman R = 0.243, *P* < 0.001; n (Normal)=113, n (Tumor)=1113. ** *P* < 0.01.

An analysis of breast cancer samples and normal samples from The TCGA database was conducted to assess the expression levels of the TBX20 gene and its correlation with ABCC1 gene expression. The results indicated that, the expression of TBX20 in tumor tissue was significantly higher than that in normal tissue (*P* < 0.01) (Fig 1A). Using Spearman correlation analysis, a scatter plot was plotted between the expression of TBX20 and ABCC1 genes. The results showed a significant positive correlation between the expression of TBX20 and ABCC1 genes (R = 0.243, *P* < 0.001) (Fig 1B).

### Overexpression of TBX20 promotes doxorubicin resistance in breast cancer cells

**Fig 2:**
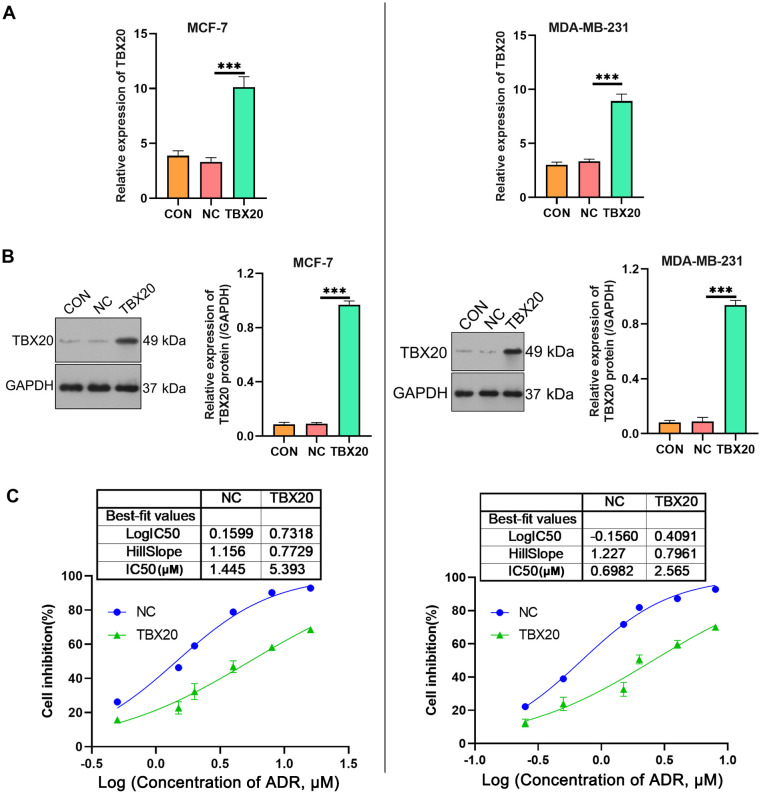
Overexpression of TBX20 enhances doxorubicin resistance in breast cancer cells. **(A)** qPCR and **(B)** Western blot analyses confirming overexpression of TBX20 in MCF-7 and MDA-MB-231 cells following lentiviral transduction. **(C)** CCK-8 assay-based dose-response curves for doxorubicin (Adriamycin, ADR) treatment in TBX20-overexpressing and negative control (NC) cells. IC_50_ values were determined using nonlinear regression analysis. Data are presented as mean ± SD (n = 3); *** *P* < 0.001. CON: Untreated control cells. NC: Negative Control; TBX20: Overexpression of TBX20; ADR: Adriamycin; IC_50_: Half maximal inhibitory concentration.

Through qPCR detection, it was found that the expression level of TBX20 in MCF-7 and MDA-MB-231 cells was significantly higher than that in the CON group and NC group (*P* < 0.001) (Fig 2A). The Western blot results showed that the expression of TBX20 protein was significantly increased in the TBX20 treatment group (*P* < 0.001) (Fig 2B). The analysis of cell inhibition rate after treatment with doxorubicin showed that the IC_50_ values of the TBX20 treatment group were significantly higher in both cell lines, indicating that TBX20 enhanced cell resistance to doxorubicin (Fig 2C).

### Suppression of TBX20 enhances chemosensitivity to doxorubicin in breast cancer cells

**Fig 3:**
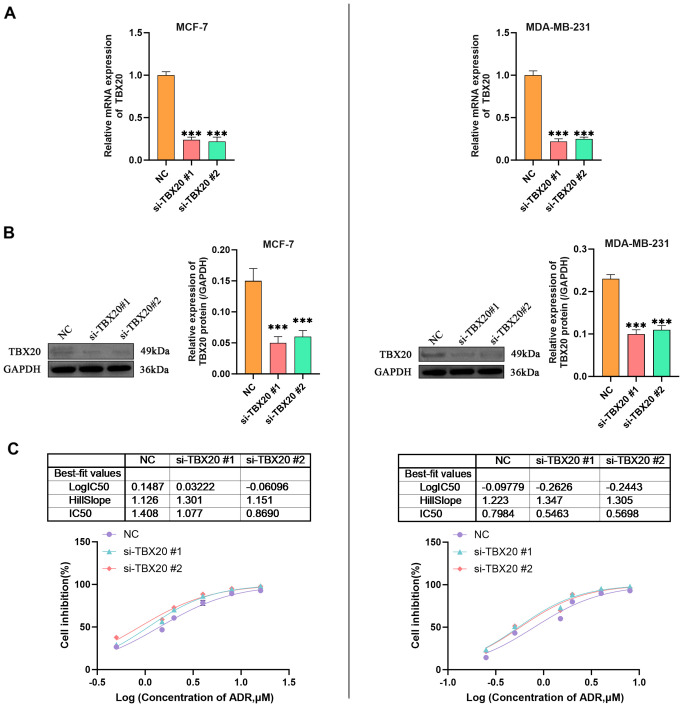
Suppression of TBX20 enhances chemosensitivity to doxorubicin in breast cancer cells. **(A)** qPCR and **(B)** Western blot analyses confirming silencing of TBX20 in MCF-7 and MDA-MB-231 cells. **(C)** CCK-8 assay-based dose-response curves for doxorubicin (Adriamycin, ADR) treatment in TBX20-suppressing and negative control (NC) cells. IC_50_ values were determined using nonlinear regression analysis. Data are presented as mean ± SD (n = 3); *** *P* < 0.001. NC: Negative Control; si-TBX20: suppression of TBX20 (#stands for different target); ADR: Adriamycin; IC_50_: Half maximal inhibitory concentration.

qPCR analysis revealed that TBX20 expression was significantly lower in both MCF-7 and MDA-MB-231 cells compared to the NC groups (*P* < 0.001; Fig 3A). Consistent with this, western blot results demonstrated a marked reduction in TBX20 protein levels following its suppression (*P* < 0.001; Fig 3B). Assessment of cell viability after doxorubicin treatment showed that the half-maximal inhibitory concentration (IC_50_) was significantly decreased in TBX20-suppressed cells across both lines (Fig 3C). This indicates that inhibition of TBX20 sensitizes breast cancer cells to doxorubicin.

### TBX20 overexpression promotes proliferation and migration of breast cancer cells

**Fig 4:**
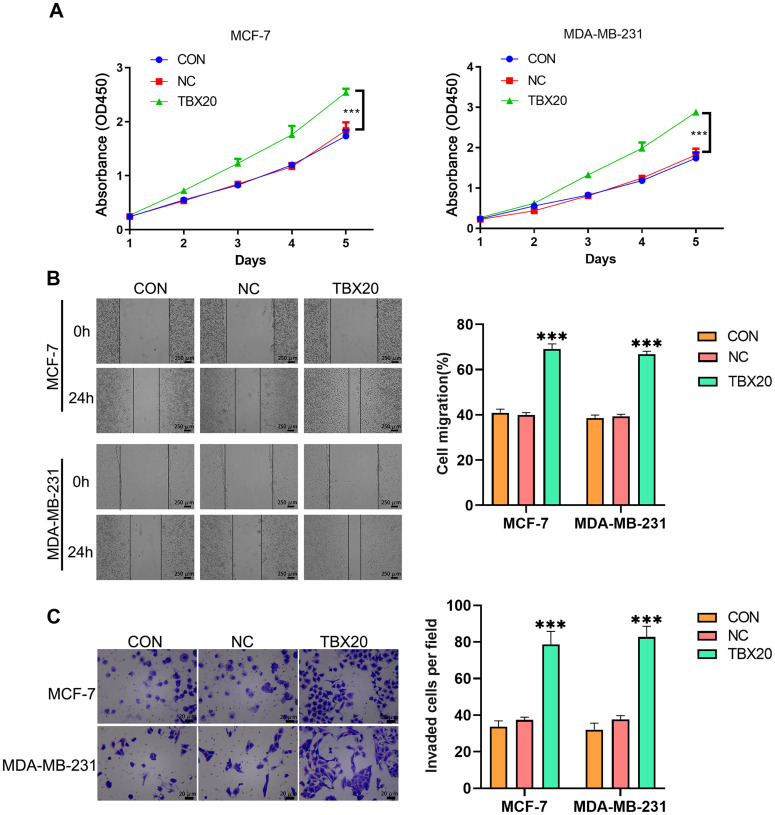
Overexpression of TBX20 promotes proliferation and migration of breast cancer cells. **(A)** CCK-8 assay showing that TBX20 overexpression enhances cell proliferation in MCF-7 and MDA-MB-231 cells over 5 days. **(B)** Wound healing assay assessing cell migration ability at 40 × magnification. **(C)** Transwell invasion assay at 100 × magnification. Data are presented as mean ± SD (n = 3); *** *P* < 0.001, TBX20 group vs NC group. CON: Untreated control cells. NC: Negative Control; TBX20: Overexpression of TBX20.

The proliferation of MCF-7 and MDA-MB-231 cells in different treatment groups was detected by CCK-8 method. The results showed that the cell proliferation in the TBX20 overexpression group (TBX20) was significantly higher than that in the control group (CON) and negative control (NC) group, and the difference was significant at 5 days (*P* < 0.001) (Fig 4A). The scratch test showed that the migration ability of cells in the TBX20 group was significantly enhanced, and the migration rate was significantly higher than that of the CON group and NC group after 24 h (*P* < 0.001) (Fig 4B). The Transwell invasion experiment results showed that the invasion ability of cells in the TBX20 group was significantly improved, and the number of invading cells was significantly higher than that of the CON group and NC group (*P* < 0.001) (Fig 4C).

### TBX20 overexpression inhibits mitophagy in breast cancer cells

**Fig 5:**
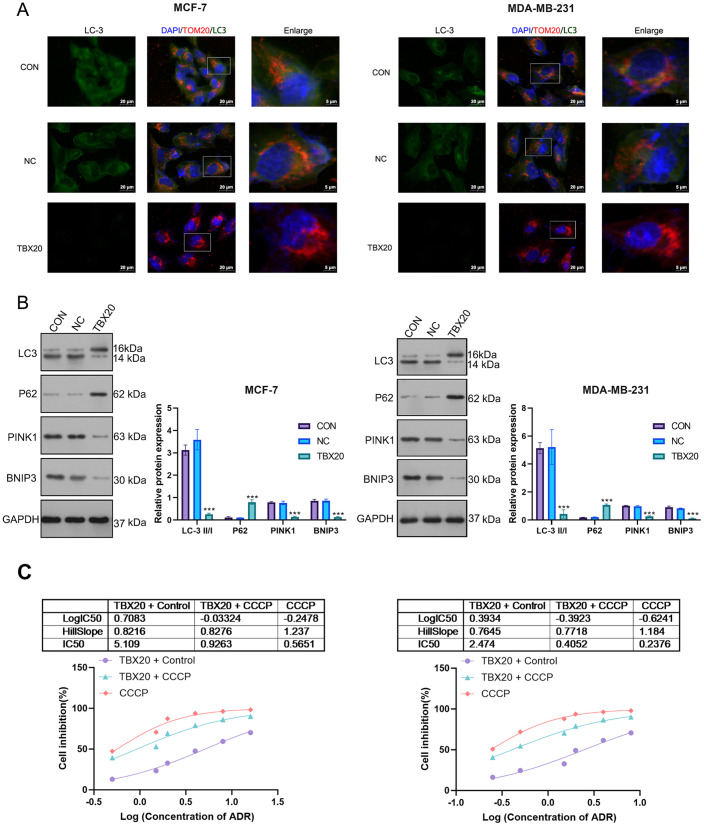
Overexpression of TBX20 inhibits mitophagy in breast cancer cells. **(A)** Immunofluorescence staining of mitochondria (TOM20, red), autophagosomes (LC3, green), and nuclei (DAPI, blue) in MCF-7 and MDA-MB-231 cells. Representative merged images show the spatial distribution of LC3 and TOM20 signals. Yellow regions in the merged channels visually indicate areas of signal overlap, which were qualitatively assessed. Representative images were acquired at 1500 × magnification; insets show enlarged regions at 5000X magnification. Scale bars: 20 μm (1500×) and 5 μm (5000×). **(B)** Western blot analysis of mitophagy-related proteins (LC3-II/I, PINK1, BNIP3, and p62) in MCF-7 and MDA-MB-231 cells transfected with TBX20-overexpressing or control vectors. **(C)** CCK-8 assay measuring IC_50_ values of doxorubicin in MCF-7 and MDA-MB-231 cells following treatment with the mitophagy inducer CCCP (10 μM, 24 h). IC_50_ values were calculated using nonlinear regression analysis. Data are presented as mean ± SD (n = 3); *** *P* < 0.001, TBX20 group vs NC group. CON: Untreated control cells. NC: Negative control; TBX20: Overexpression of TBX20; ADR: Adriamycin; IC_50_: Half maximal inhibitory concentration.

In confocal microscopy observation, the localization of LC3 (green) and TOM20 (red) in MCF-7 and MDA-MB-231 cells with TBX20 overexpression showed changes in the spatial pattern and fluorescence intensity compared to the NC and CON groups (*P* < 0.001), the TBX20 group exhibited markedly diminished LC3 puncta and reduced green fluorescence intensity (Fig 5A). The Western blot results showed that the LC3-II/LC3-I ratio in MCF-7 and MDA-MB-231 cells with TBX20 overexpression significantly decreased compared with the NC group, and the expression of PINK1 and BNIP3 was also reduced. Notably, the expression of P62 was elevated (*P* < 0.001) (Fig 5B). Dose-response curve analysis revealed that cell inhibition rates increased in a concentration-dependent manner across all groups with escalating ADR doses. Notably, the inhibition curve for the TBX20 + CCCP group was positioned highest, indicating the most potent inhibitory effect at equivalent drug concentrations. Moreover, this group exhibited a significantly lower IC_50_ value compared to the TBX20 + Control and CCCP groups, suggesting that the combined treatment markedly reduced the half-maximal inhibitory concentration. Conversely, the TBX20 + Control group yielded the highest IC_50_ value, demonstrating the lowest sensitivity to ADR (Fig 5C).

### TBX20 promotes doxorubicin resistance in breast cancer cells by increasing ABCC1 expression

**Fig 6:**
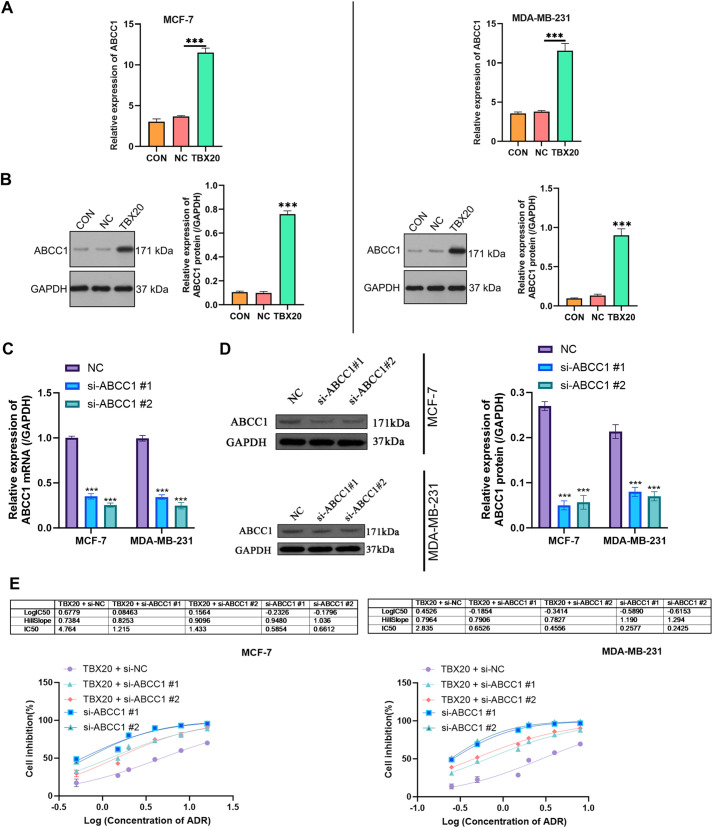
TBX20 enhances doxorubicin resistance in breast cancer cells by increasing ABCC1 expression. **(A)** Relative mRNA expression of ABCC1 in MCF-7 and MDA-MB-231 cells transfected with TBX20-overexpressing or control vectors, determined by qPCR. GAPDH was used as an internal control. **(B)** Western blot analysis confirming ABCC1 protein expression in MCF-7 and MDA-MB-231 cells following TBX20 overexpression. **(C)** Relative mRNA expression of ABCC1 in MCF-7 and MDA-MB-231 cells transfected with si-ABCC1, as determined by qPCR. **(D)** Western blot analysis confirming ABCC1 protein expression in MCF-7 and MDA-MB-231 cells. GAPDH was used as a loading control. **(E)** CCK-8 assay measuring IC_50_ values of doxorubicin in MCF-7 and MDA-MB-231 cells after co-transduction with TBX20 and si-ABCC1. IC_50_ values were determined via nonlinear regression analysis. Data are presented as mean ± SD (n = 3); *** *P* < 0.001. ADR: Adriamycin; TBX20: TBX20 overexpression with negative control siRNA; TBX20 + si-ABCC1: TBX20 overexpression with ABCC1-siRNA co-transduction.

In MCF-7 and MDA-MB-231 cells, TBX20 treatment significantly upregulated ABCC1 mRNA expression, with a notable increase in ABCC1 mRNA in the TBX20-treated group compared to the NC groups (*P* < 0.001) (Fig 6A). Western blot analysis showed that ABCC1 protein expression was significantly elevated in the TBX20 group, with higher levels of ABCC1 protein expression compared to the NC groups (*P* < 0.001) (Fig 6B). qPCR and western blot analyses were conducted to evaluate the silencing efficiency of two ABCC1-targeting siRNAs (si-ABCC1#1 and si-ABCC1#2). Both siRNAs effectively knocked down ABCC1 expression, as evidenced by decreased mRNA (Fig 6C) and protein (Fig 6D) levels. Dose-response curve analysis demonstrated that the inhibition curve for the TBX20 + si-NC group was positioned lowest, indicating the poorest sensitivity to doxorubicin and the highest IC_50_ value. In contrast, the TBX20 + si-ABCC1 #1 and TBX20 + si-ABCC1 #2 groups exhibited significantly higher inhibition rates and markedly reduced IC50 values compared to the control group. Furthermore, both si-ABCC1 #1 and #2 groups alone also displayed enhanced drug sensitivity and lower IC_50_, suggesting that silencing ABCC1effectively potentiates the cytotoxic effects of doxorubicin (Fig 6E).

**Fig 7:**
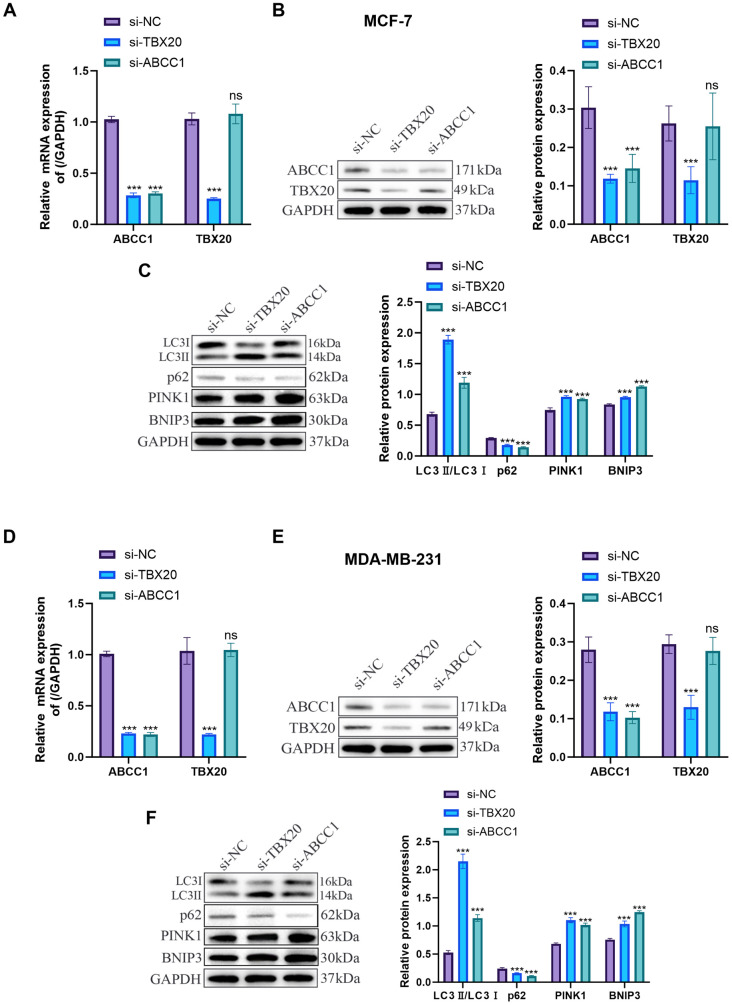
Silencing of ABCC1 or TBX20 alters the expression of autophagy-related proteins. **(A)**: qPCR analysis of ABCC1and TBX20 mRNA expression. **(B)** Western blot analysis of ABCC1 and TBX20 protein levels. **(C)** Western blot analysis of autophagy markers (LC3-I/II, p62) and mitophagy-associated proteins (PINK1, BNIP3). **(D)** Relative mRNA expression levels of ABCC1and TBX20 by qPCR. **(E)** Protein expression levels of ABCC1 and TBX20 by Western blotting. **(F)** Protein levels analysis of LC3-I/II, p62, PINK1, and BNIP3 by Western blotting. Data are presented as mean ± SD (n = 3); ***P < 0.001 compared with the si-NC group, ns: no significant difference. The si-TBX20 group refers to cells transfected with the TBX20 siRNA duplex si-TBX20#1, and the si-ABCC1 group refers to cells transfected with the ABCC1siRNA duplex si-ABCC1#1. The si-NC group indicates cells transfected with a non-targeting negative control siRNA.

In both MCF-7 and MDA-MB-231 cells, qPCR analysis revealed that the mRNA expression levels of ABCC1and TBX20 were significantly downregulated in the si-TBX20 and si-ABCC1 groups compared with the si-NC group (*P* < 0.001) (Fig 7A and 7D). Consistent with these findings, Western blot assays showed that the protein levels of ABCC1 and TBX20 were markedly decreased in both the si-TBX20 and si-ABCC1 groups relative to the si-NC group (*P* < 0.001) (Fig 7B and 7E). Furthermore, regarding autophagy-related proteins, the ratio of LC3-II/LC3-I was significantly increased, while the expression of p62 was notably reduced in both silencing groups (*P* < 0.001). Concurrently, the mitophagy-related proteins PINK1 and BNIP3 were also significantly upregulated (*P* < 0.001) (Fig 7C and 7F).

### TBX20 promotes the transcriptional activity of ABCC1 by directly binding to its promoter

**Fig 8:**
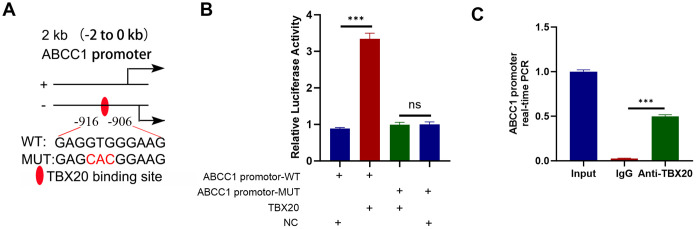
TBX20 promotes the transcriptional activity of ABCC1 by directly binding to its promoter. **(A)** Prediction of TBX20 binding sites in the ABCC1 promoter region and design of mutant sequences using the JASPAR database. **(B)** Dual-luciferase reporter assay showing the activation of the ABCC1 promoter by TBX20. **(C)** Chromatin immunoprecipitation assay demonstrating the direct binding of TBX20 to the ABCC1 gene promoter. Precipitated DNA was analyzed by qPCR. Data are presented as percentage of input. IgG was used as a negative control. ChIP: chromatin immunoprecipitation; WT: wild type; MUT: mutant; *** *P* < 0.001.

The results showed that a potential TBX20 binding sequence was predicted within the −916 to −906 region of the ABCC1 promoter, and a corresponding mutant construct was generated to disrupt this binding site (Fig 8A). The dual-luciferase reporter assay demonstrated that ectopic expression of TBX20 significantly increased the luciferase activity of the wild-type ABCC1 promoter, whereas mutation of the binding site markedly attenuated the TBX20-induced promoter activation, with no significant difference between the mutant and the control group (Fig 8B). Furthermore, chromatin immunoprecipitation followed by qPCR analysis revealed that ABCC1 promoter DNA precipitated by the anti-TBX20 antibody was significantly enriched compared with the IgG negative control and remained markedly elevated after normalization to input DNA, indicating that TBX20 directly binds to the ABCC1 gene promoter region (Fig 8C). Collectively, these findings demonstrate that TBX20 enhances ABCC1 transcriptional activity through specific binding to its promoter.

## Discussion

Breast cancer is one of the most prevalent malignancies among women globally, particularly noted for its high drug resistance and metastatic propensity, which significantly complicate treatment strategies [12]. Therefore, identifying new therapeutic targets and enhancing drug efficacy is crucial. Previous studies have demonstrated that the absence of the TBX20 gene results in mid-gestational embryonic lethality and significant cardiac morphological abnormalities in mice, suggesting a critical role in essential physiological processes [13]. Additionally, research by Tang et al. indicated that TBX20 can improve myocardial contractility and mitochondrial function, thereby directly ameliorating human cardiac remodeling [9] However, the specific role and mechanism of TBX20 in breast cancer remain unclear. Garc í a-Flores et al. found that differences in DNA methylation of the TBX20 gene can be associated with septal defects [14]. The 5-hydroxymethylation of DNA and the 5-methylation of RNA play a regulatory role in the expression of TBX20 during early human development [15]. The present findings clearly demonstrate that TBX20 is significantly upregulated in breast cancer samples, and its overexpression is associated with promoted proliferation and migration of breast cancer cells, as well as significantly increasing resistance to doxorubicin. This discovery contradicts previous studies that suggested an inhibitory role of TBX20 in colorectal cancer [16], implying that TBX20 may have diverse mechanisms of action in different cancer types. Future studies are required to further explore the specific mechanisms of TBX20 in breast cancer, which will not only elucidate its role in the pathological process but also provide critical theoretical support for the development of therapeutic strategies targeting TBX20.

Research indicates that mitophagy is significantly impacted in cancer. Key receptors and adaptors, such as PINK1, Parkin, BNIP3, BNIP3L/NIX, and p62/SQSTM1, along with associated signaling pathways, have shown impaired functionality across various cancers [17]. In this study, we discovered that overexpression of TBX20 in breast cancer cells is correlated with inhibited mitophagy levels and suppresses the expression of LC3 II/I, PINK1, and BNIP3 proteins. LC3, a crucial autophagy protein, and its form LC3 II/I, are widely used as indicators of autophagic activity within cells [18]. The accumulation of PINK1 protein and its association with BNIP3 on the outer mitochondrial membrane are critical steps in triggering mitophagy [19]. TBX20 reduces mitophagic activity by inhibiting the expression of LC3 II/I, PINK1, and BNIP3 proteins, a finding that was validated by immunofluorescence assays. Importantly, restoring mitophagy with CCCP treatment significantly reversed TBX20-induced doxorubicin resistance. Collectively, these data reveal that TBX20 promotes doxorubicin resistance in breast cancer cells by inhibiting mitophagy. The suppression of mitophagy by TBX20 likely disrupts mitochondrial homeostasis, leading to the accumulation of damaged mitochondria and subsequently triggering robust compensatory survival signals. This process may drive the transition of cells toward a more aggressive and drug-resistant phenotype. These findings align with observations from other studies under specific conditions, where deficiencies in mitophagy due to the loss of specific molecules have been shown to promote breast cancer tumor progression [20]. Similarly, in ERα-positive breast cancer, ADP-ribosylation factor-like protein 3 upregulates the deubiquitinase to stabilize ERα protein, and the concomitant inhibition of mitophagy contributes to tumorigenesis and endocrine resistance [21]. This paradigm suggests that TBX20 may confer drug resistance by locking cells into a state characterized by low autophagy, high stress, and heightened defense mechanisms.

ABCC1 is closely associated with multidrug resistance in breast cancer, primarily through its role in the transport of various chemotherapeutic agents [22]. ABCC1 occupies a significant position in the study of multidrug resistance due to its ability to transport a multitude of drugs and substances such as the immunostimulatory cyclic GMP-AMP (cGAMP) [23]. Studies have shown that overexpression of ABCC1 in breast cancer cells significantly enhances resistance to various chemotherapeutic drugs, manifested through enhanced drug efflux mechanisms [24]. Furthermore, ABCC1 is involved in the TGF-β-induced epithelial-to-mesenchymal transition process, which is not only critical to tumor progression but also plays a significant role in the invasiveness and metastasis of breast cancer [25]. However, no studies to date have reported on the regulatory effects of TBX20 on ABCC1 influencing drug resistance in breast cancer. The present findings establish a direct regulatory link, demonstrating that TBX20 transcriptionally upregulates ABCC1 expression, as evidenced by chromatin immunoprecipitation and dual-luciferase reporter assays. Furthermore, silencing ABCC1 reversed the doxorubicin resistance induced by TBX20 overexpression. These complementary gain- and loss-of-function experiments position ABCC1 as a key downstream effector of TBX20. This result aligns with the known upregulation of ABCC1 leading to multidrug resistance in breast cancer [24]. Importantly, while ABCC1 is established as a drug efflux pump, its potential role in modulating cellular processes like mitophagy is not well-defined. The data suggest that TBX20-induced ABCC1 upregulation is associated with changes in mitophagy markers. However, it remains to be determined whether ABCC1 plays a direct role in the mitophagy process itself or influences it indirectly. This represents an intriguing avenue for future research.

In addition to the ABCC1-mediated drug efflux pathway, recent studies have revealed that mitochondrial dynamics and redox balance also play essential roles in modulating doxorubicin resistance. For instance, aberrant mitochondrial fission and fusion processes contribute to the survival and chemoresistance of triple-negative breast cancer cells, highlighting mitochondrial dynamics as a potential therapeutic target [26]. Furthermore, the accumulation of autophagolysosomes following doxorubicin treatment is partly driven by mitochondrial ROS, and that elimination of mitochondrial ROS can alleviate this process and promote resistance in breast cancer cells [27]. Given that this study showed TBX20 is associated with suppressed mitophagy and downregulates PINK1 and BNIP3, it is plausible that TBX20 may also affect mitochondrial dynamics and ROS-mediated autophagic responses, thereby contributing to doxorubicin resistance through mechanisms beyond ABCC1 regulation. Future studies are warranted to elucidate whether TBX20 modulates mitochondrial morphology or redox signaling pathways as part of its role in chemoresistance.

However, this study also has limitations. Firstly, although our findings offer significant insights at the cellular level, the lack of in vivo validation of TBX20’s effects limits the generalizability of our findings. Future studies should employ animal models to validate our observations. Secondly, the correlation between TBX20 and ABCC1 expression in TCGA data, while statistically significant, shows a relatively weak correlation coefficient, suggesting that ABCC1 may be one of multiple downstream targets through which TBX20 exerts its effects in breast cancer. Thirdly, while a direct transcriptional regulation of ABCC1 by TBX20 was established and both were linked to altered mitophagy and drug resistance, the precise mechanistic connection between ABCC1 upregulation and the observed suppression of mitophagy markers requires deeper investigation. Fourthly, regarding the assessment of mitophagy, further and more definitive observations (e.g., using electron microscopy or dynamic monitoring with LC3 dual-fluorescence autophagy flux markers) are required to solidify our conclusions. These aspects will be explored further in our subsequent research.

In summary, the present study demonstrates that TBX20 enhances the resistance of breast cancer cells to doxorubicin by transcriptionally upregulating ABCC1 and is correlated with the suppression of mitophagy. This discovery not only provides new targeted strategies for the treatment of breast cancer but also lays an important foundation for the future development of medications and optimization of treatment protocols.

## Supporting information

S1 File Uncropped Western blot bands. (DOCX)
