# Supplementary material for: TBX20 promotes doxorubicin resistance in breast cancer cells through enhanced ABCC1 expression and inhibition of mitophagy
Source: PLoS One. 2026 Jul 17;21(7):e0353376. doi: 10.1371/journal.pone.0353376 (PMC13379107; doi:10.1371/journal.pone.0353376)

**Maker**:


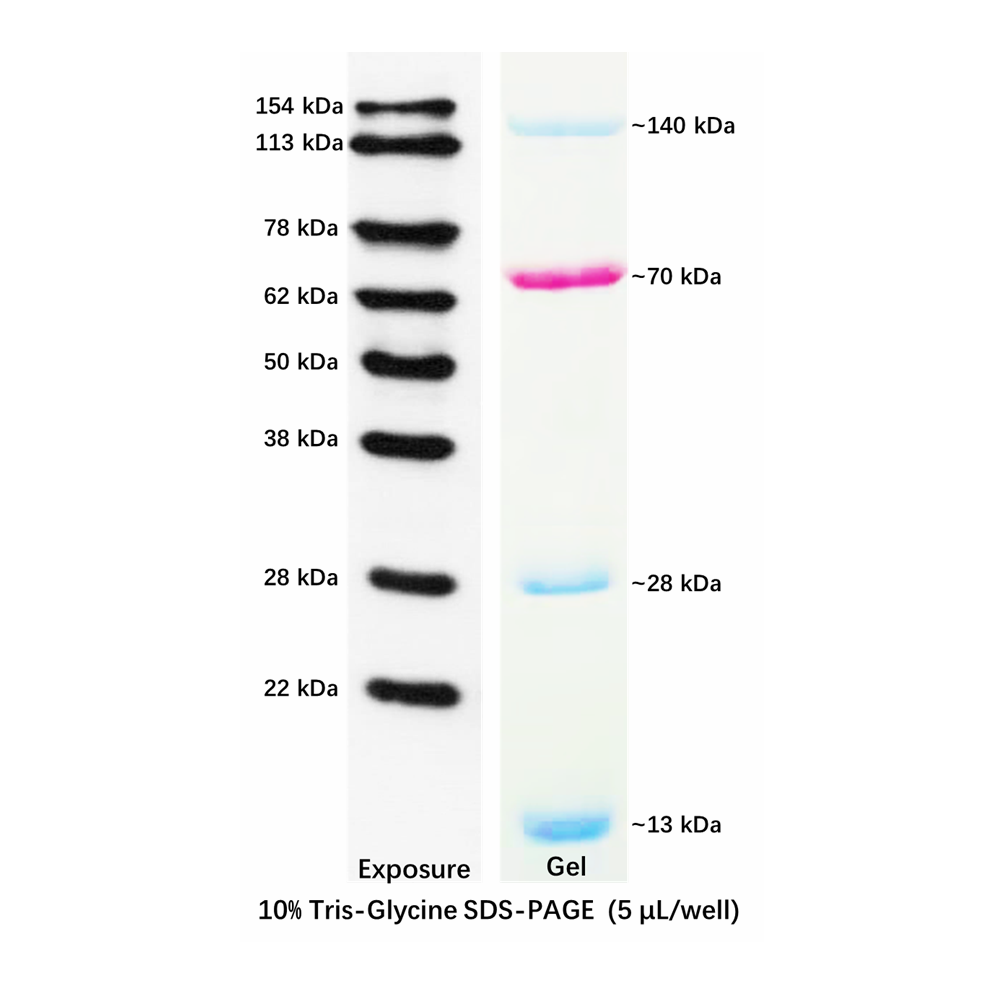

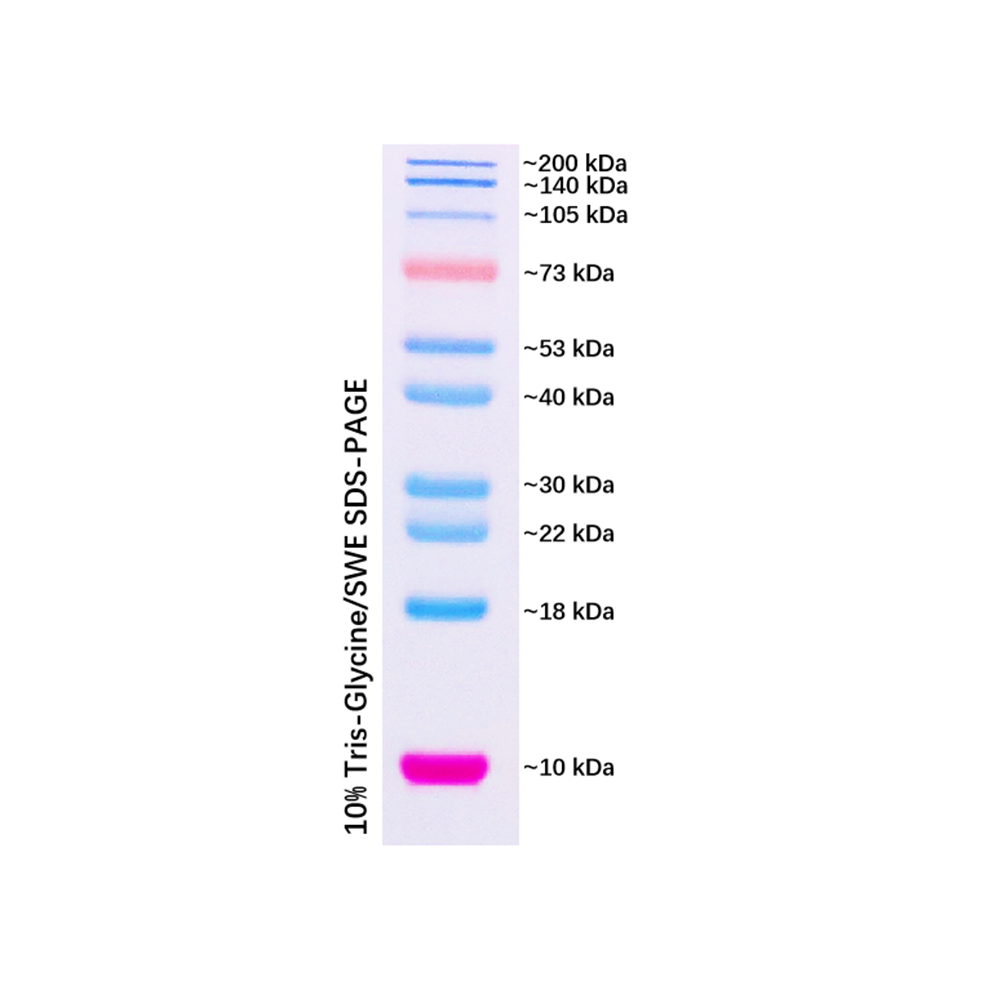


The stripes marked with red line are the ones used in this article

Figure 2B, MCF-7: TBX20: 49 KDa


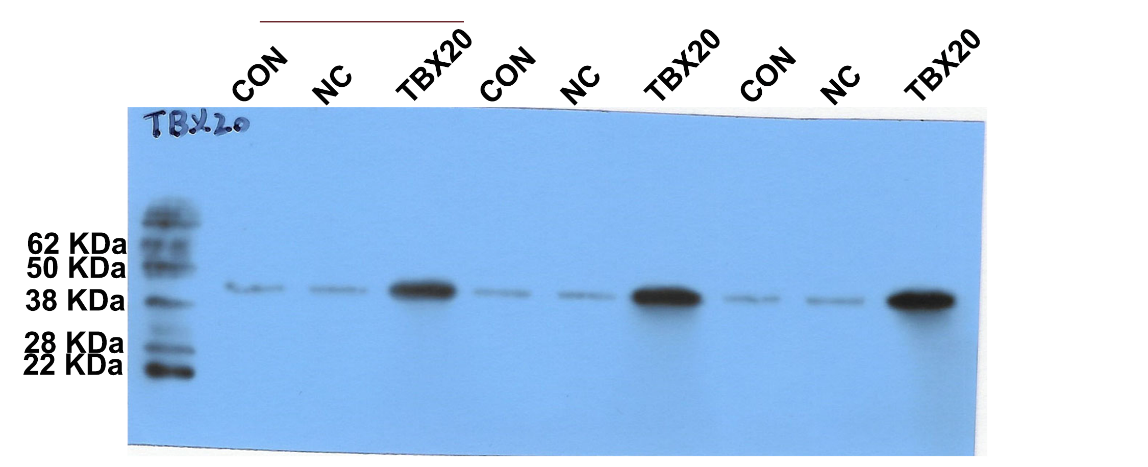


Figure 2B, MCF-7: GAPDH: 37 KDa


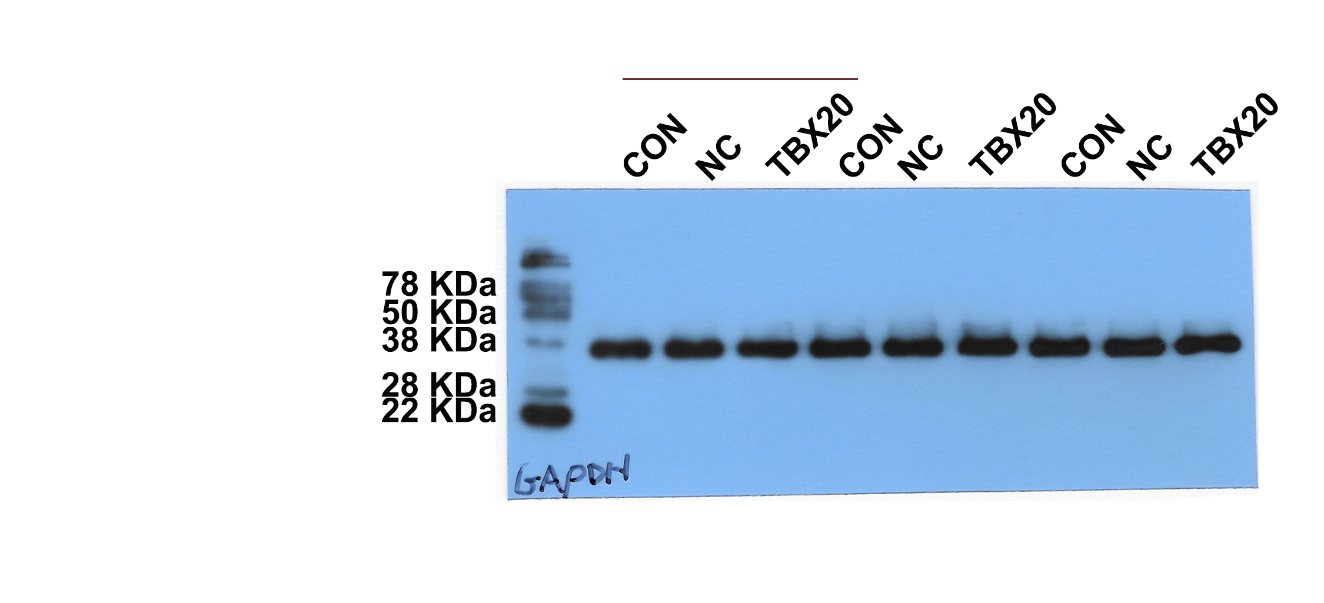


Figure 2B, MDA-MB-231: TBX20: 49 KDa


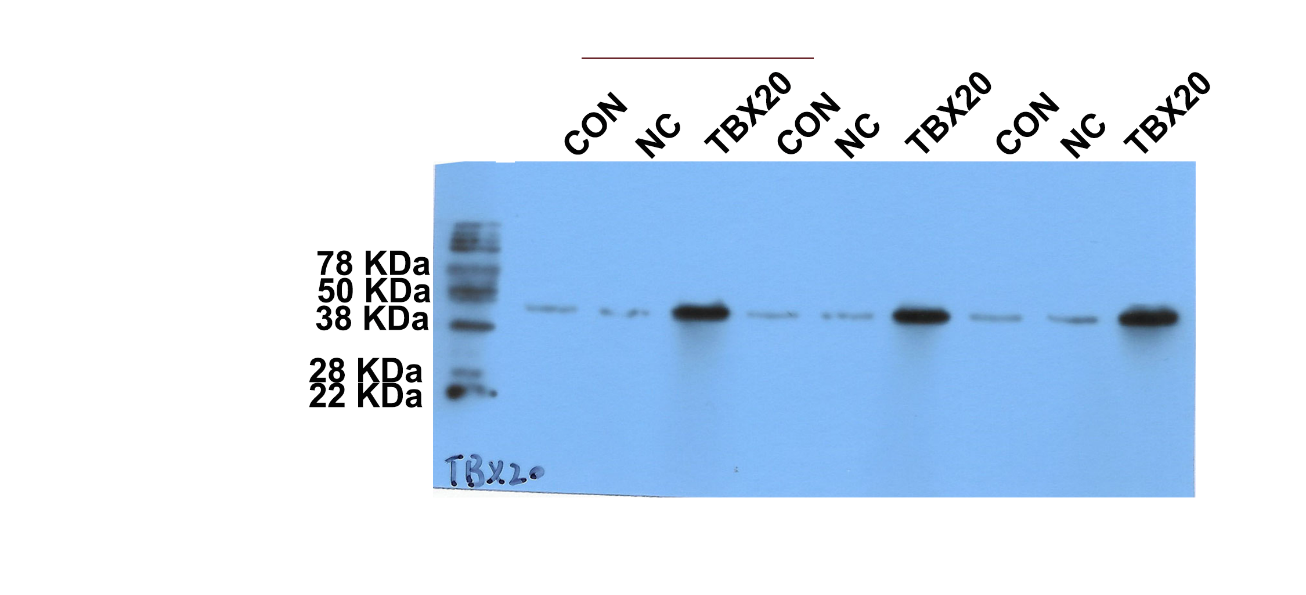


Figure 2B, MDA-MB-231: GAPDH: 37 KDa


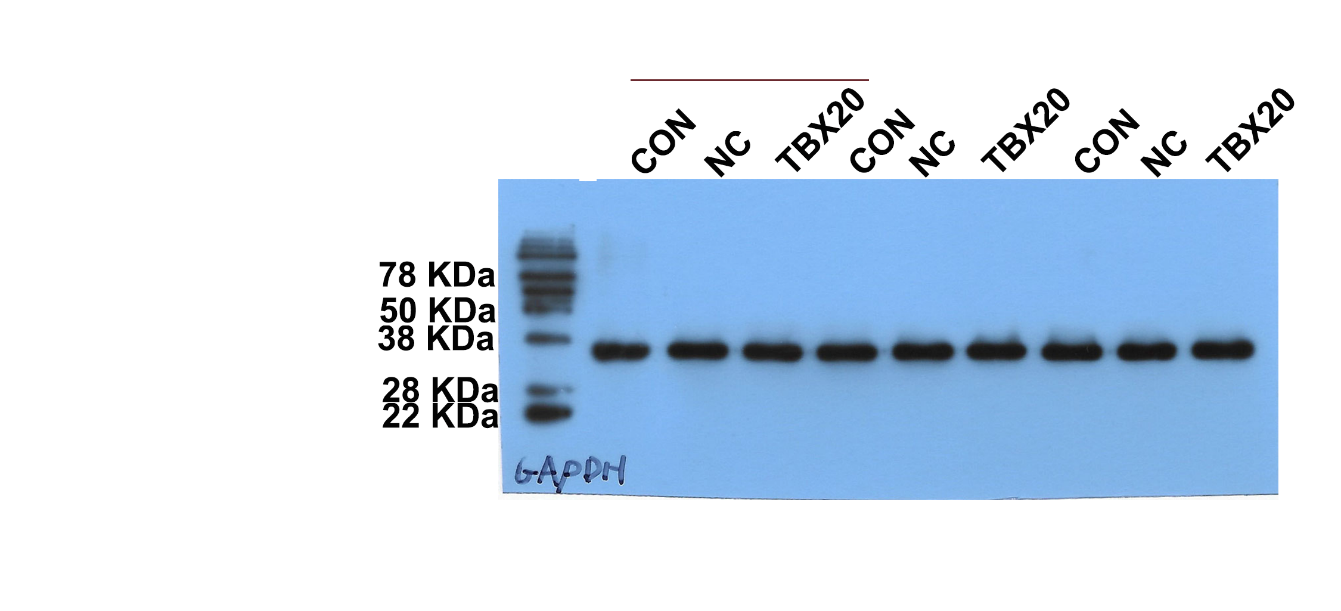


Figure 3B, MCF-7: TBX20: 49 KDa


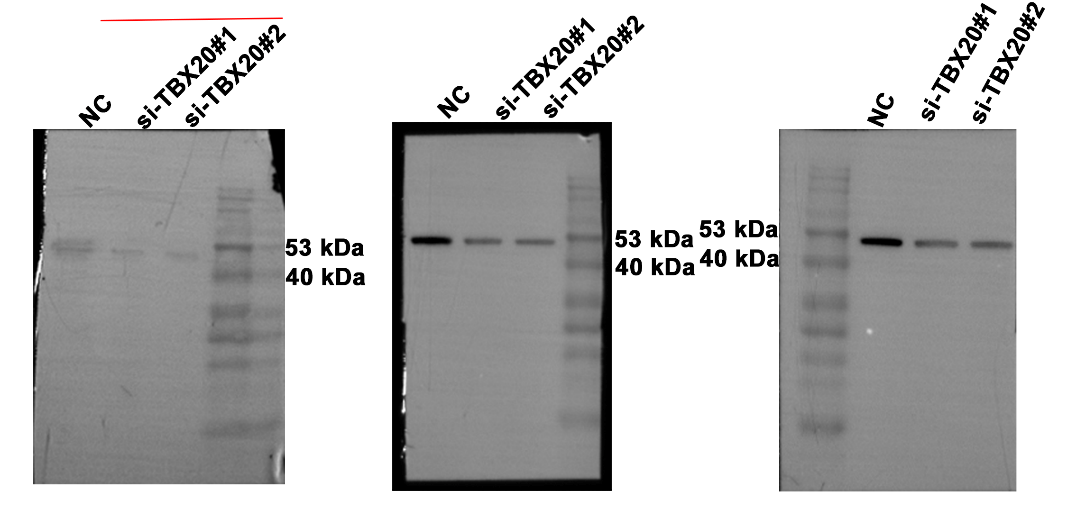


Figure 3B, MCF-7: GAPDH: 37 KDa


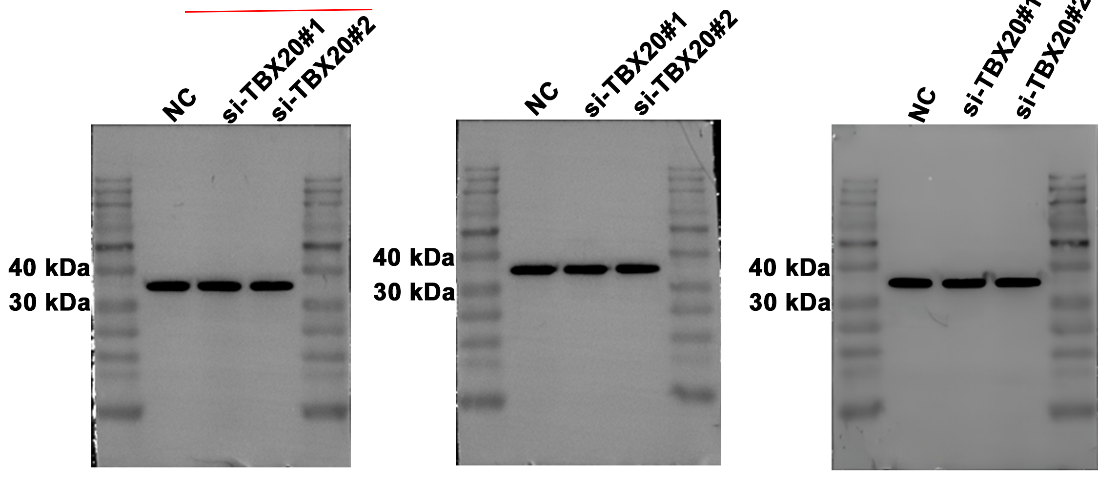


Figure 3B, MDA-MB-231: TBX20: 49 KDa


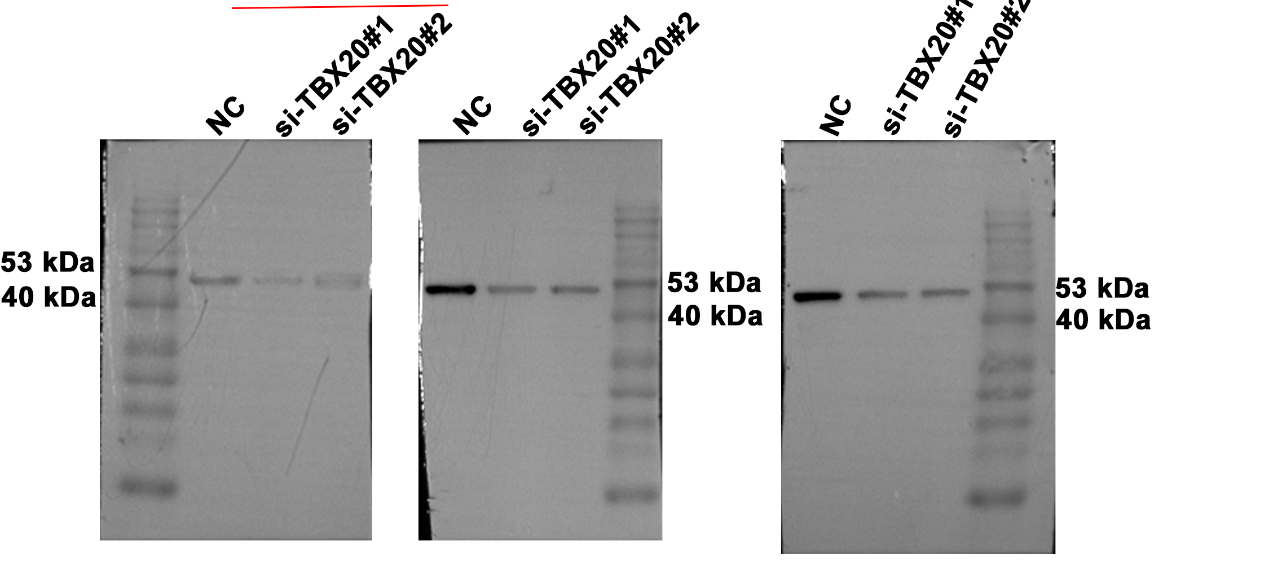


Figure 3B, MDA-MB-231: GAPDH: 37 KDa


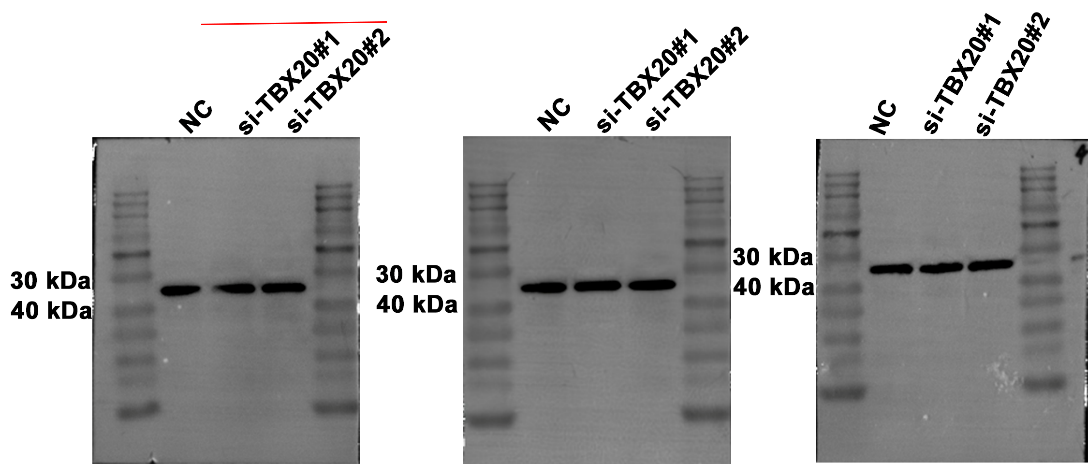


Figure 5B, MCF-7: BNIP3: 30 KDa


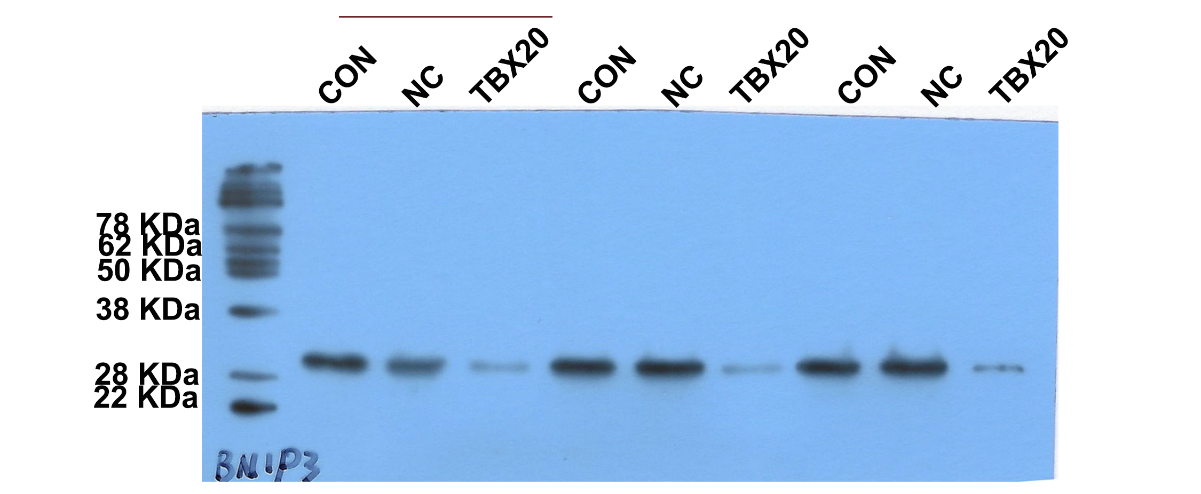


Figure 5B, MCF-7: GAPDH: 37 KDa


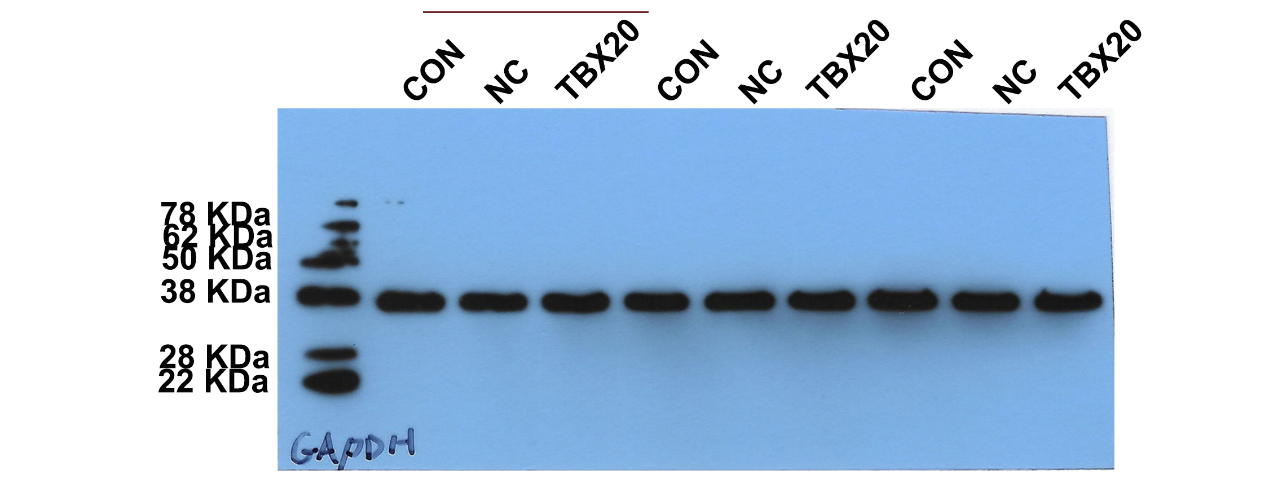


Figure 5B, MCF-7: LC3: 16 KDa, 14 KDa


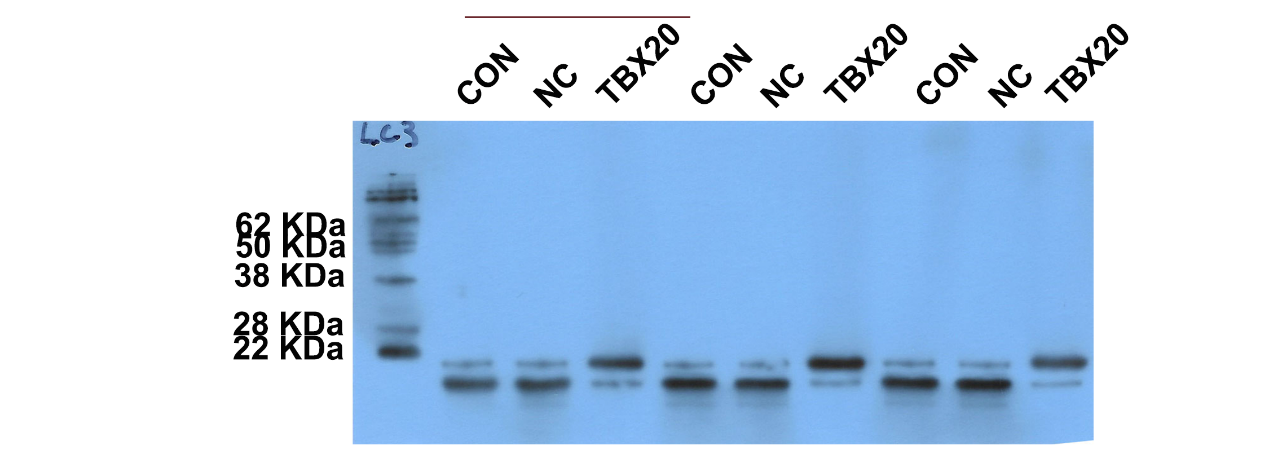


Figure 5B, MCF-7: P62: 62 KDa


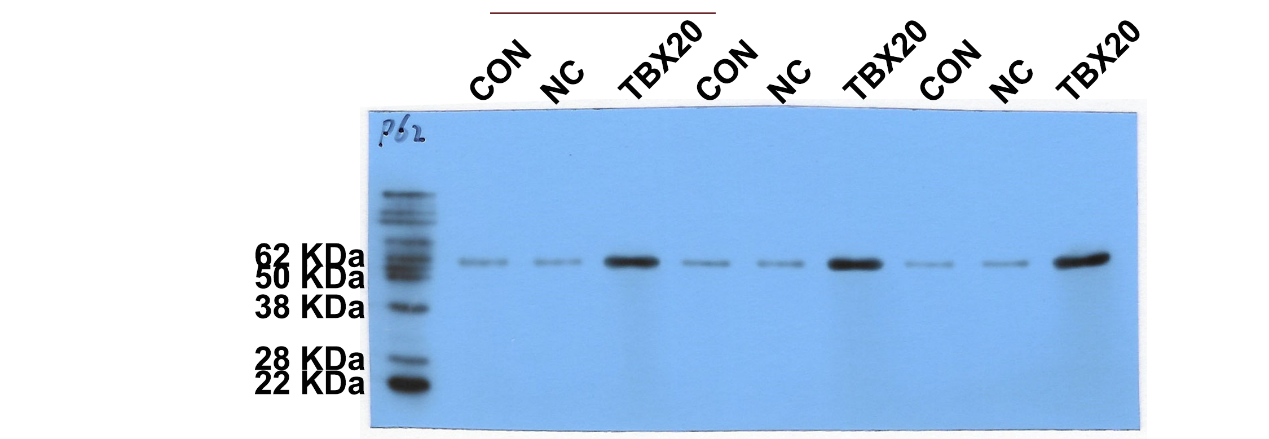


Figure 5B, MCF-7: PINK1: 63 KDa


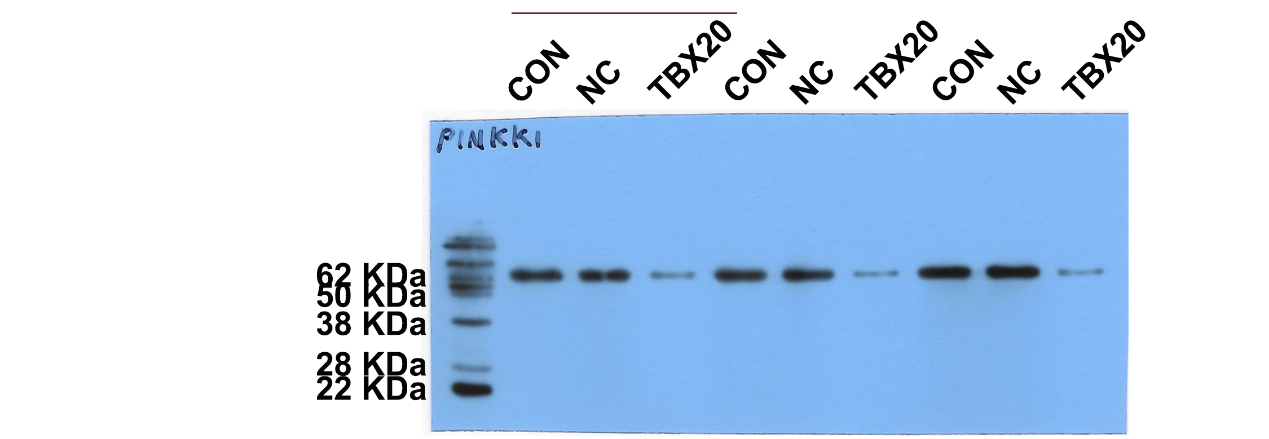


Figure 5B, MDA-MB-231: LC3: 16 KDa, 14 KDa


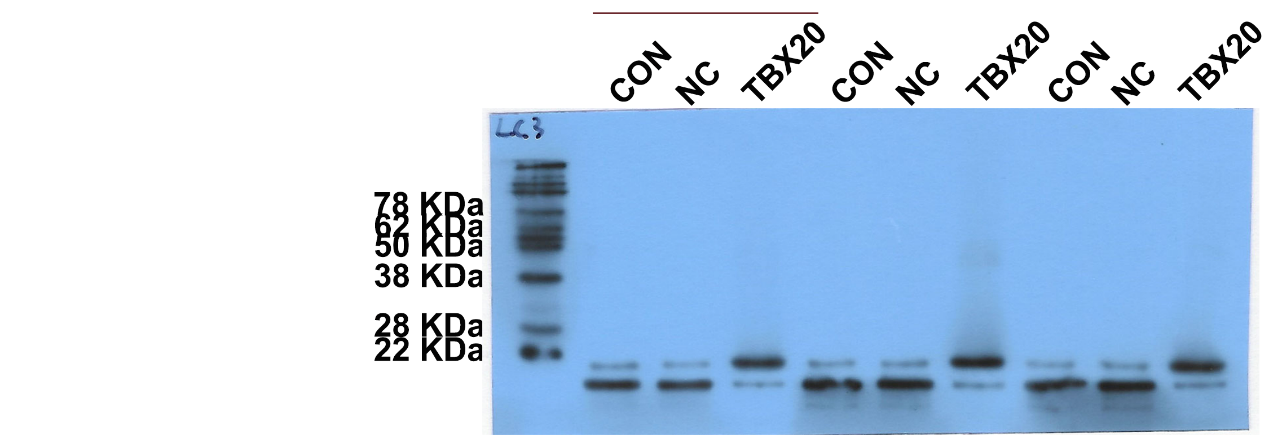


Figure 5B, MDA-MB-231: BNIP3: 30 KDa

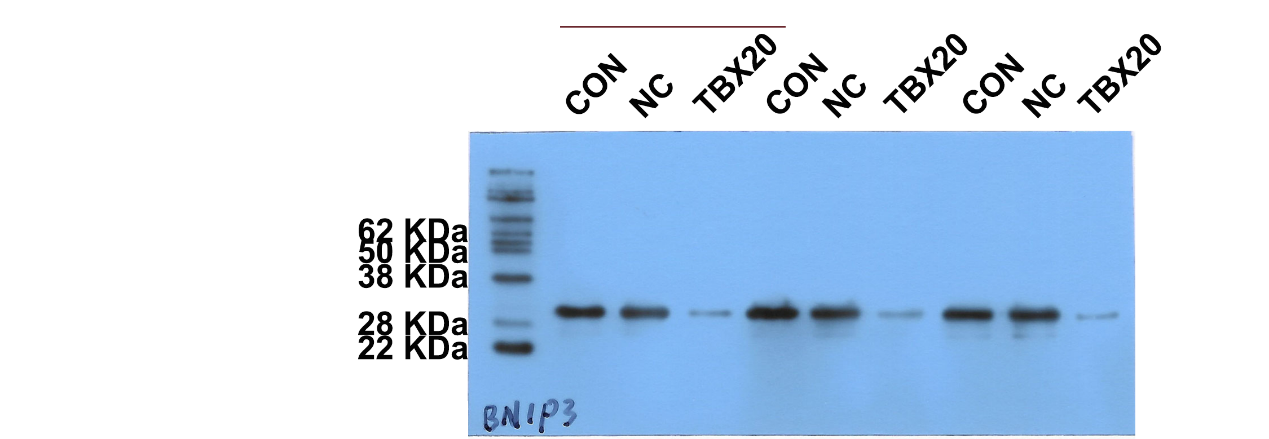


Figure 5B, MDA-MB-231: GAPDH: 37 KDa


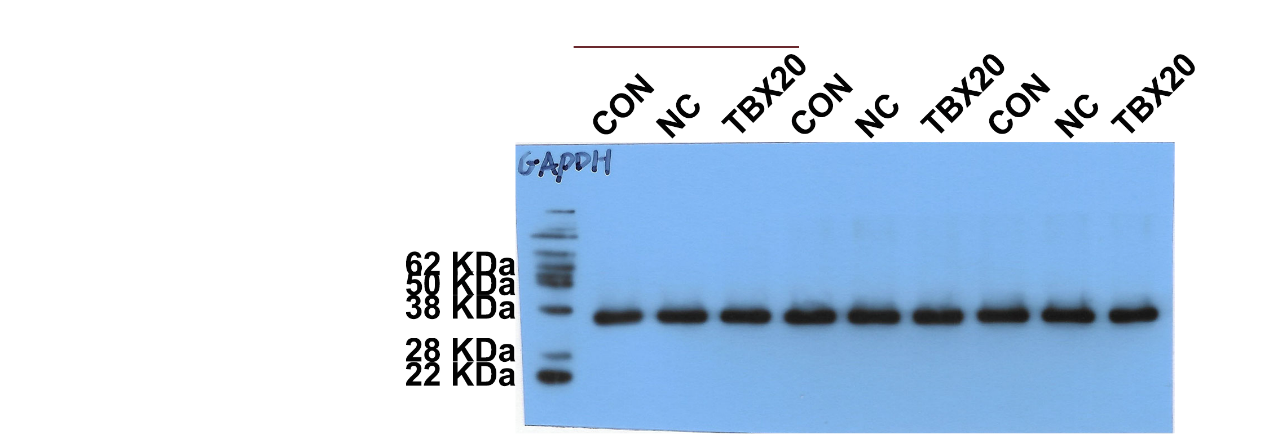


Figure 5B, MDA-MB-231: P62: 62 KDa


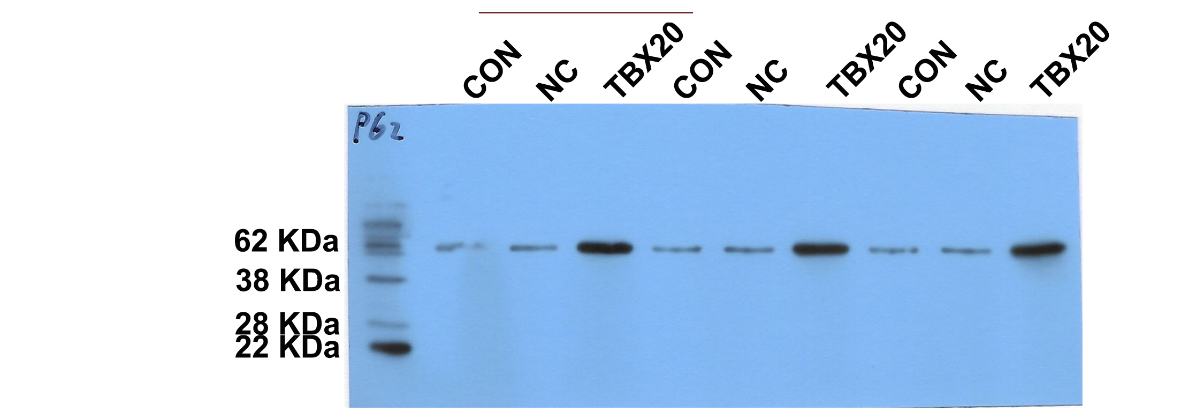


Figure 5B, MDA-MB-231: PINK1: 63 KDa


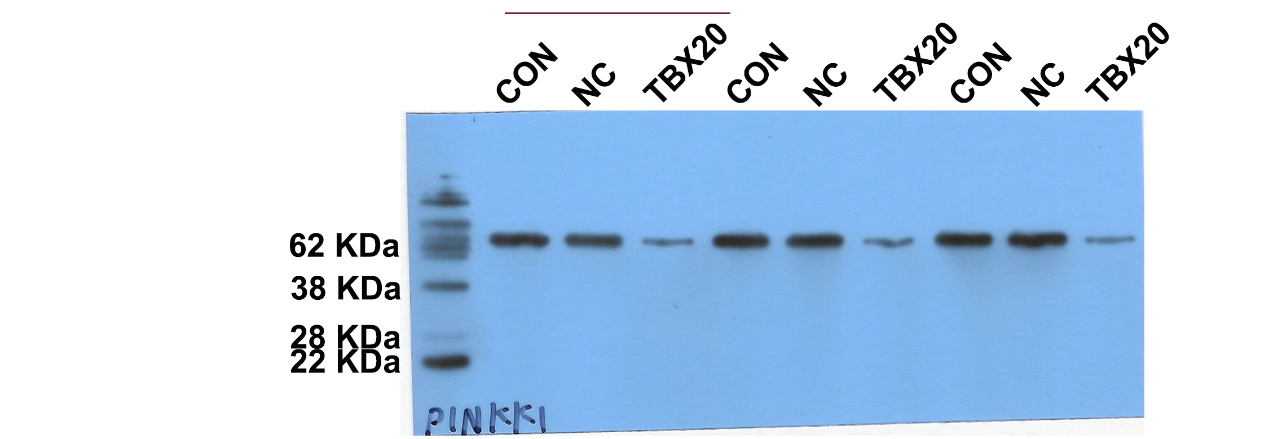


Figure 5B, MCF-7: ABCC1: 171 KDa


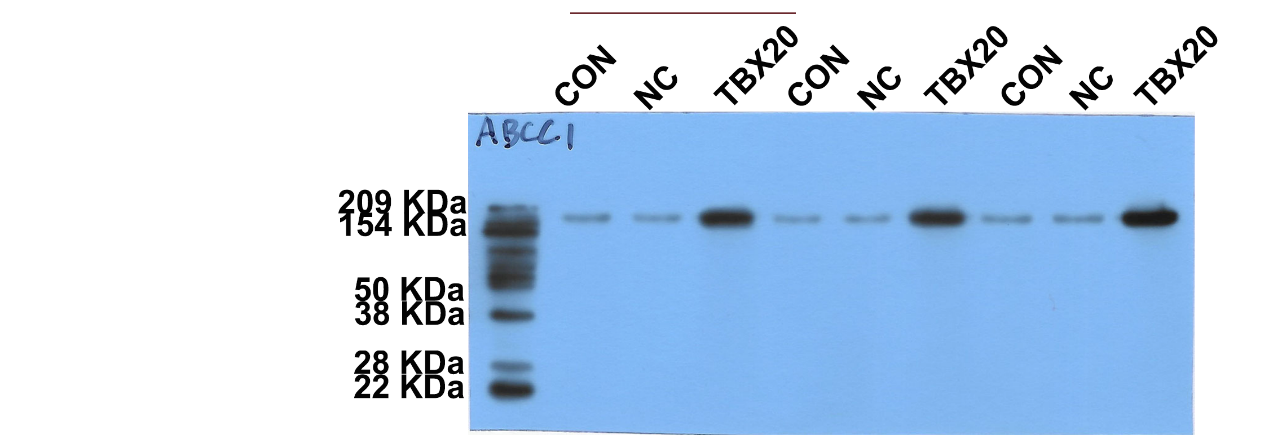


Figure 6B, MCF-7: GAPDH: 37 KDa


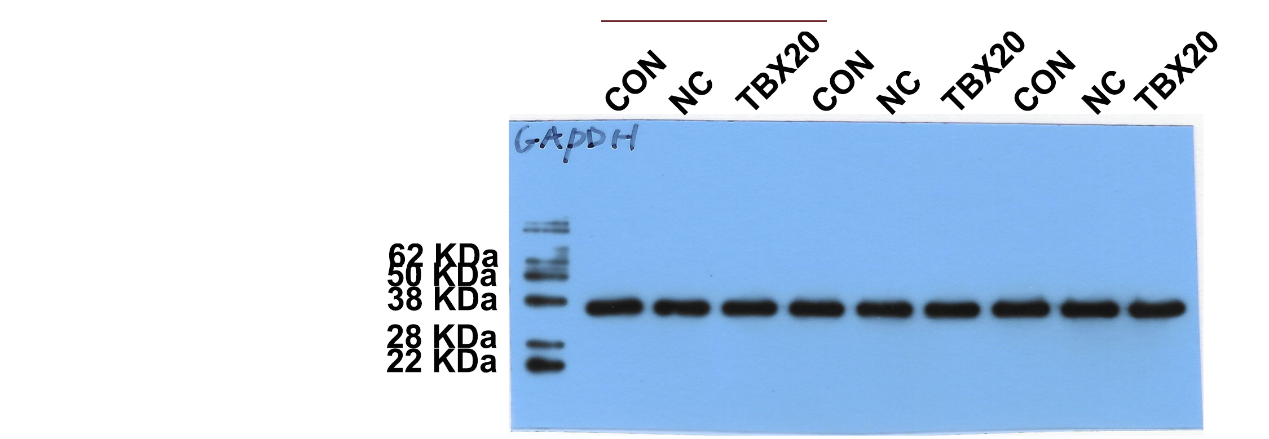


Figure 6B, MDA-MB-231: ABCC1: 171 KDa


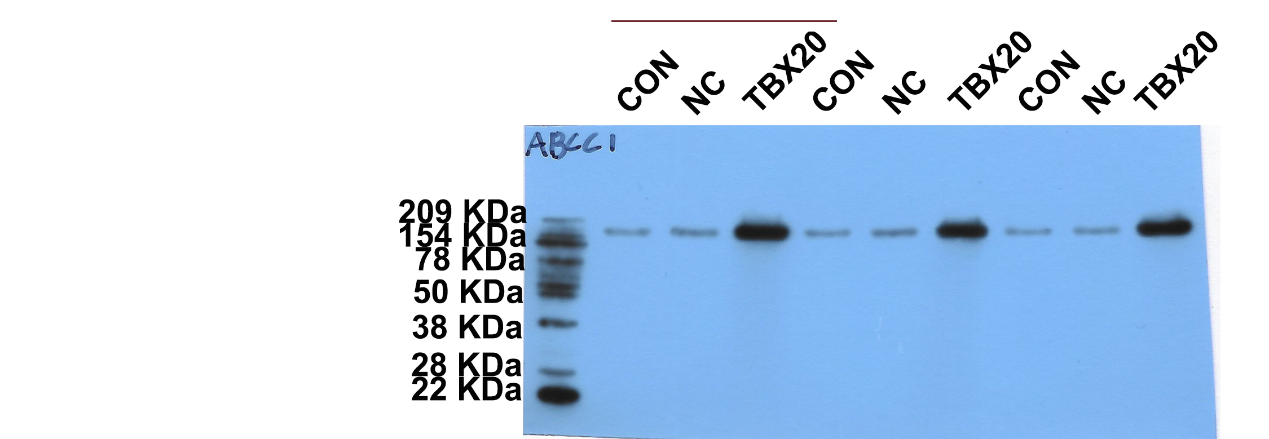


Figure 6B, MDA-MB-231: GAPDH: 37 KDa


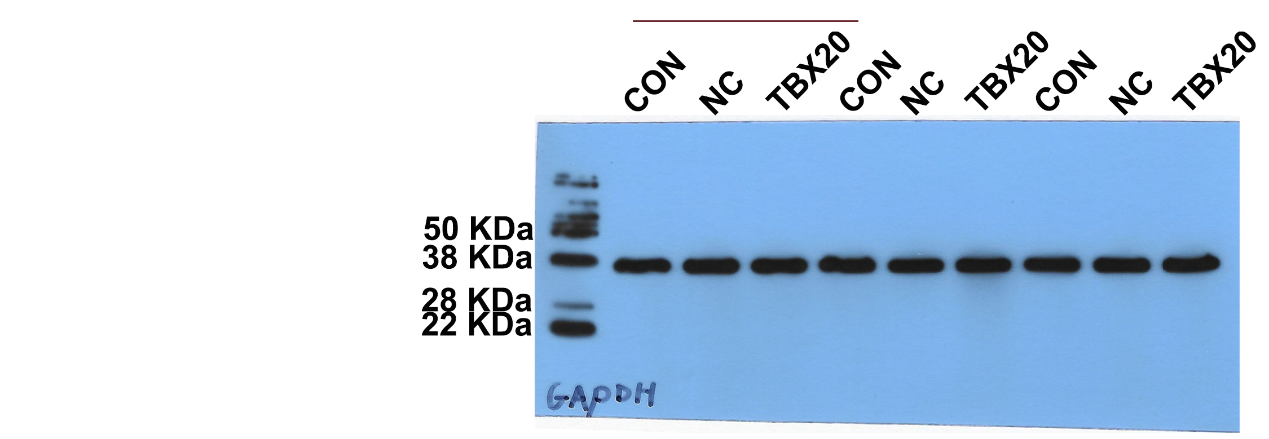


Figure 6C, MCF-7: ABCC1: 171 KDa


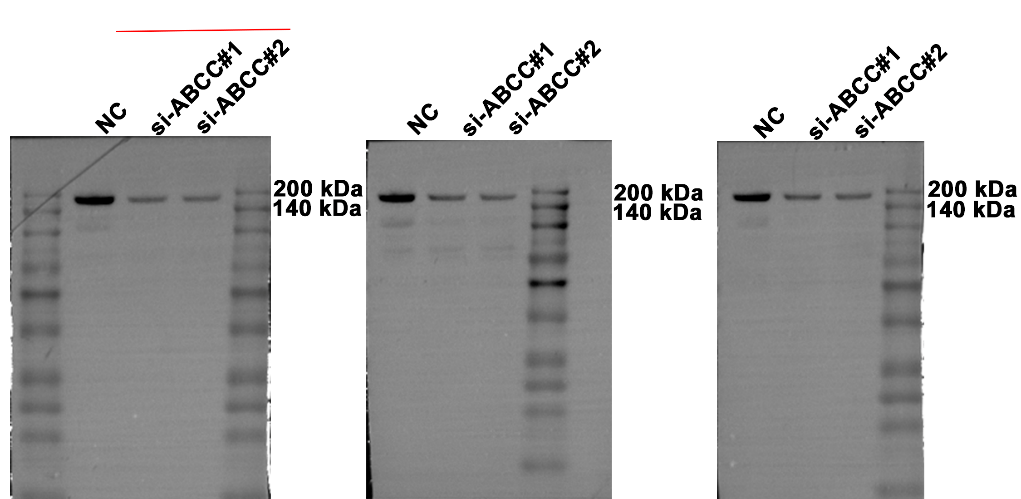


Figure 6C, MCF-7: GAPDH: 37 KDa


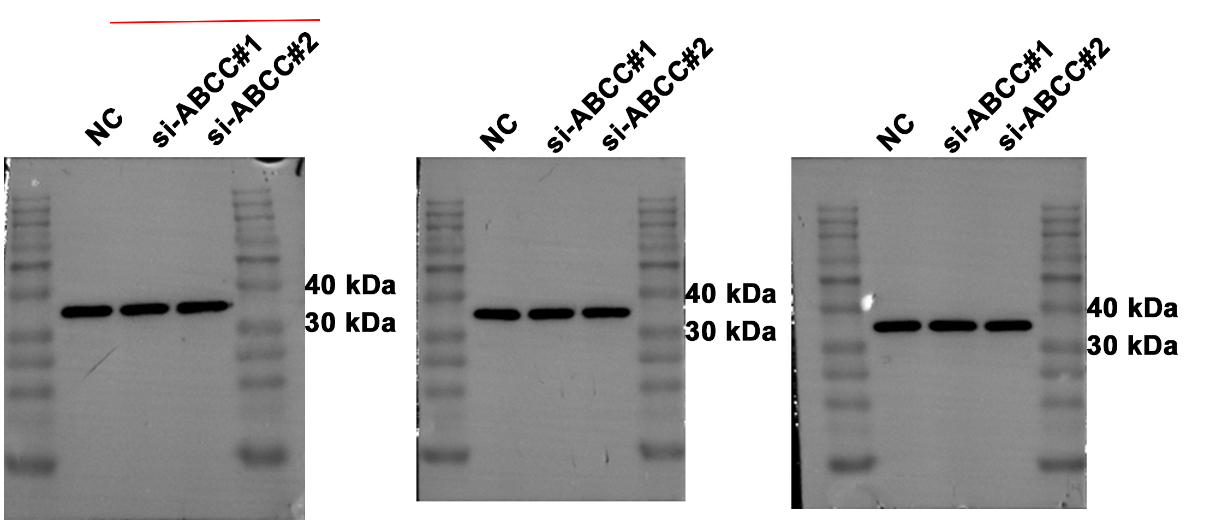


Figure 6C, MDA-MB-231: ABCC1: 171 KDa


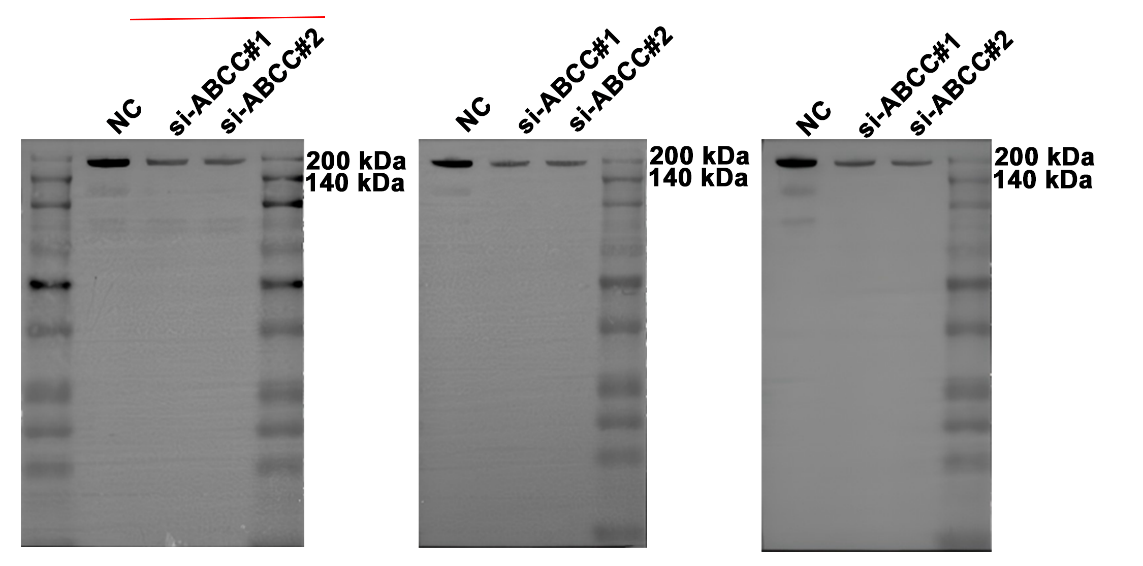


Figure 6C, MDA-MB-231: GAPDH: 37 KDa


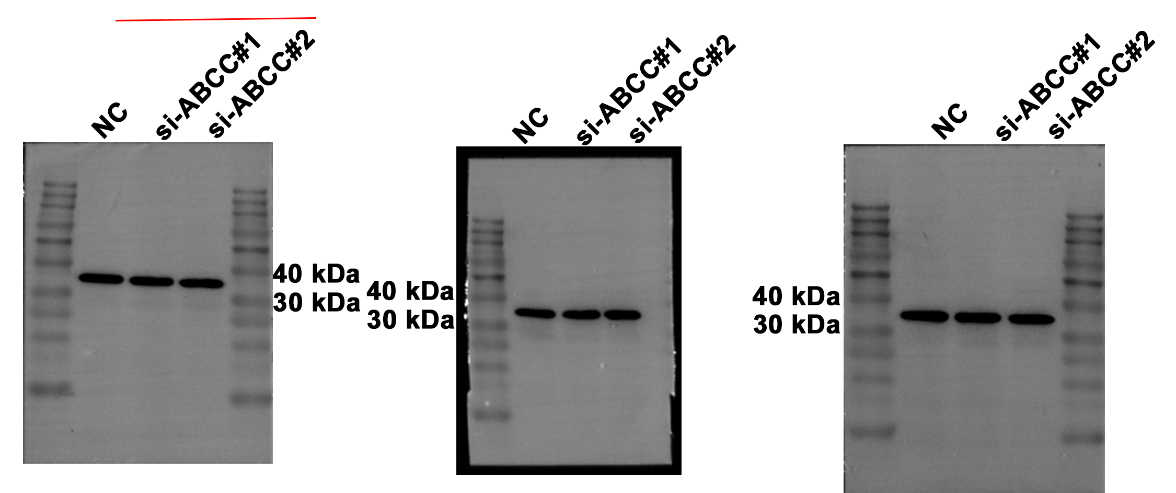


Figure 7B, MCF-7: ABCC1: 171 KDa


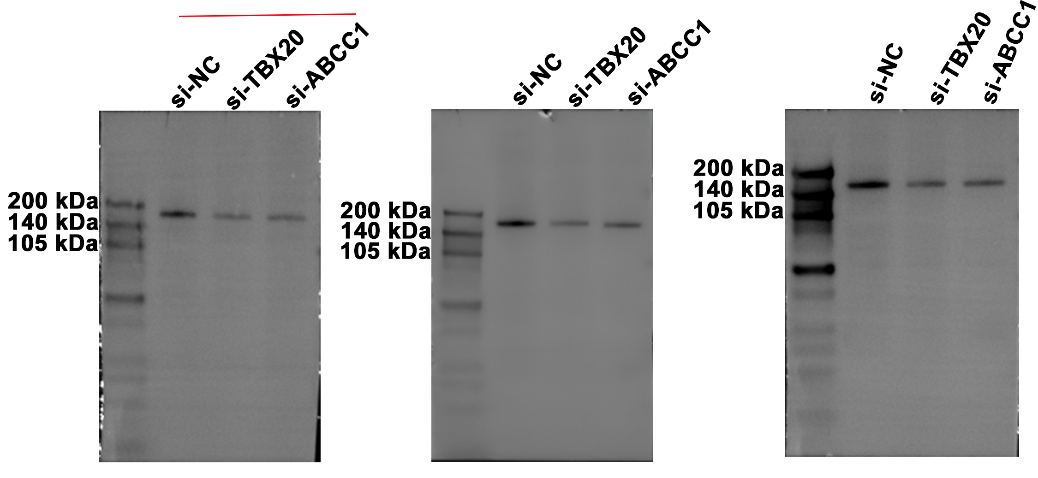


Figure 7B, MCF-7: TBX20: 49 KDa


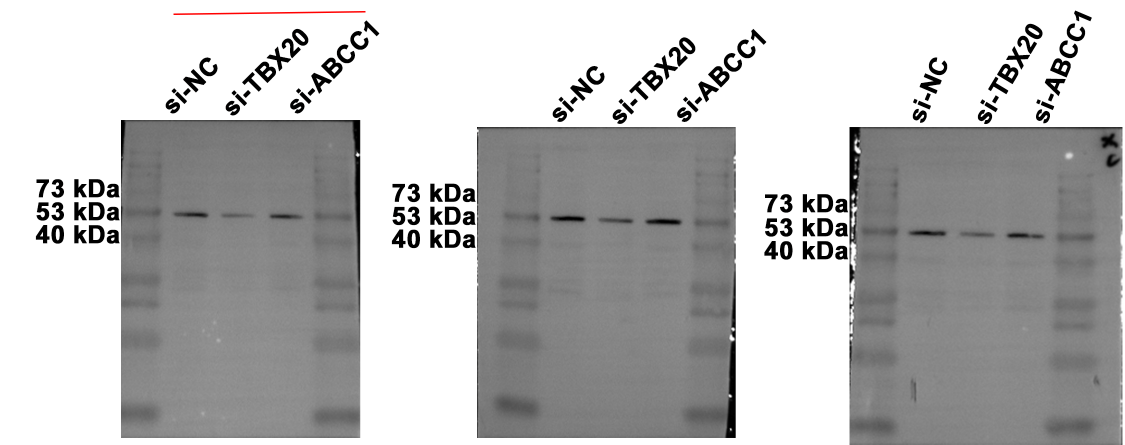


Figure 7B, MCF-7: GAPDH: 37 KDa


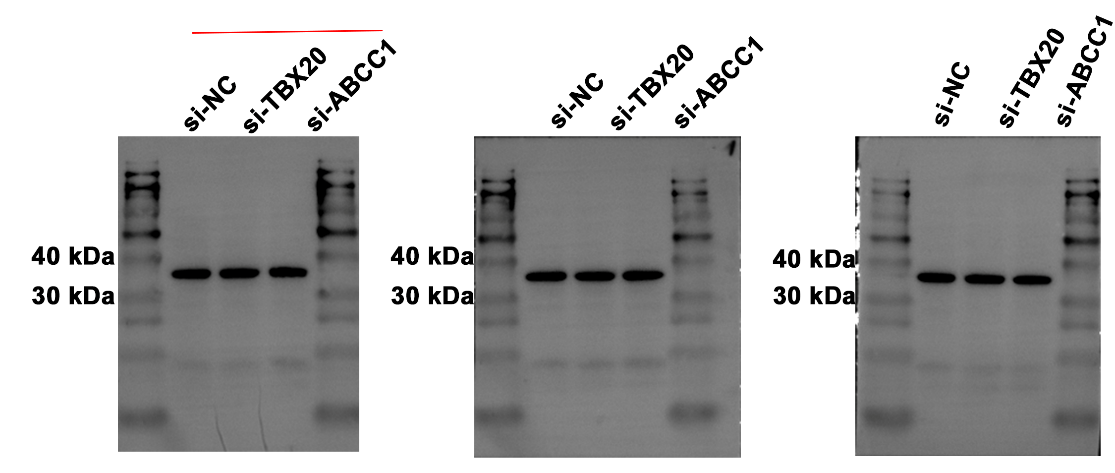


Figure 7C, MCF-7: BNIP3: 30 KDa


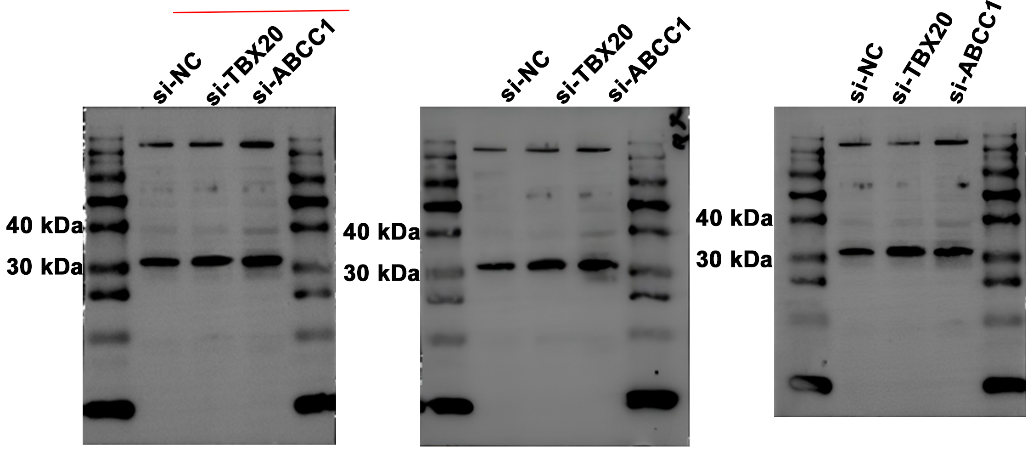


Figure 7C, MCF-7: GAPDH: 37 KDa


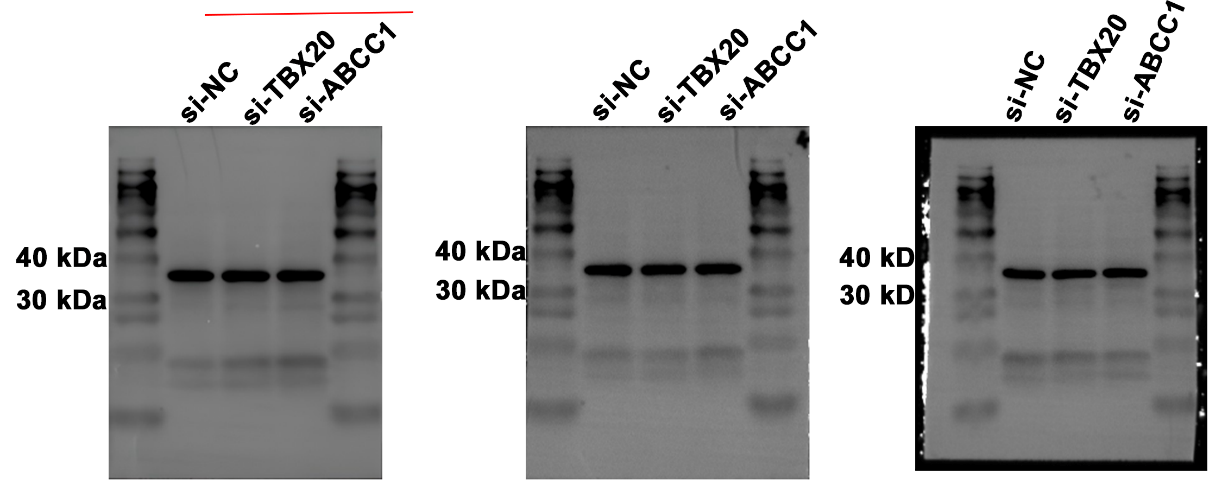


Figure 7C, MCF-7: LC3: 16 KDa, 14 KDa


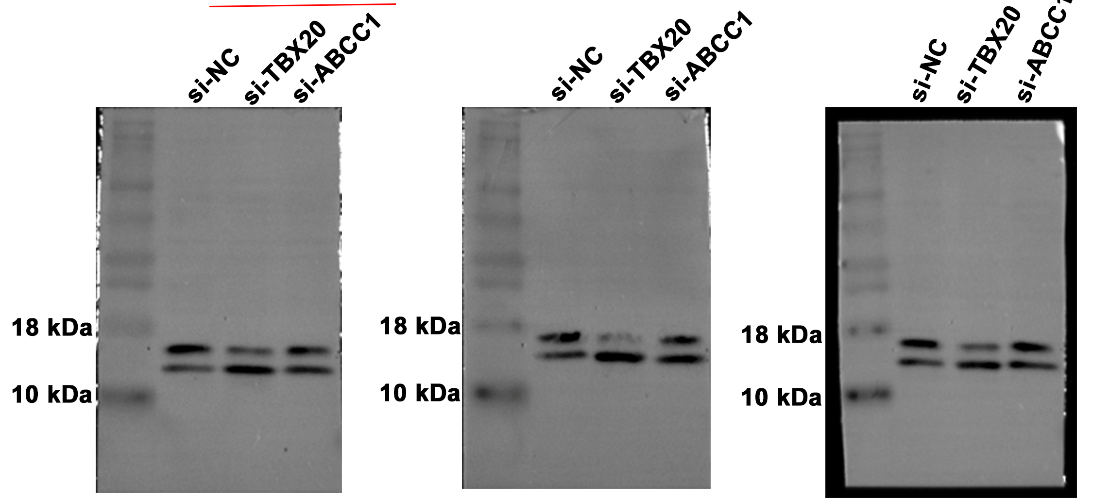


Figure 7C, MCF-7: P62: 62 KDa


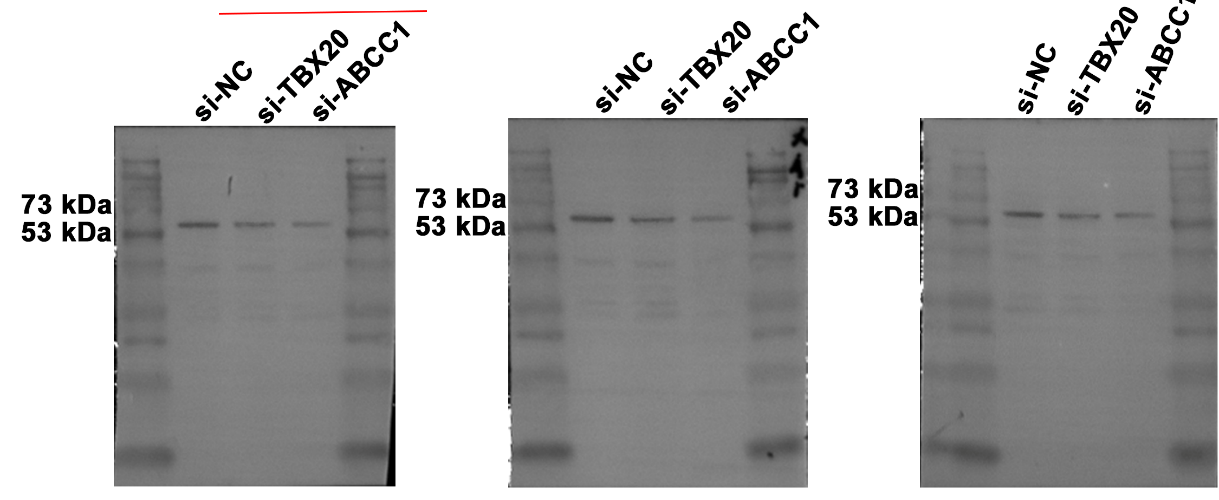


Figure 7C, MCF-7: PINK1: 63 KDa


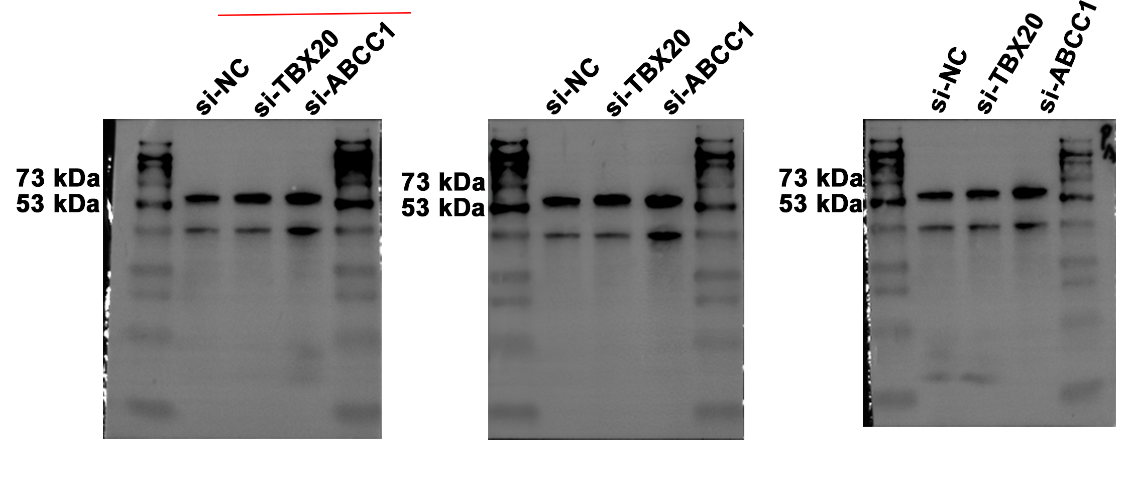


Figure 7E, MDA-MB-231: ABCC1: 171 KDa


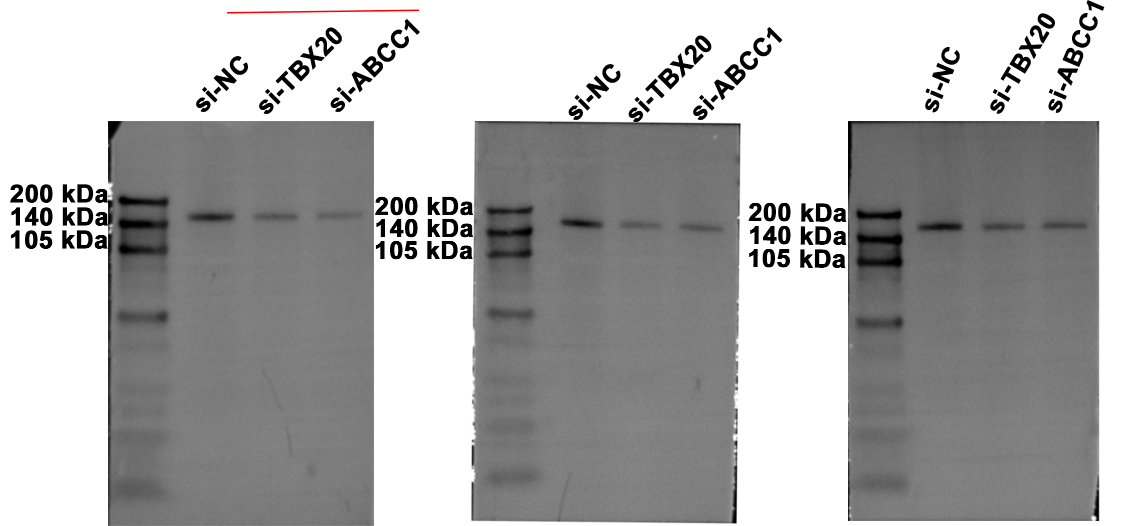


Figure 7E, MDA-MB-231: TBX20: 49 KDa


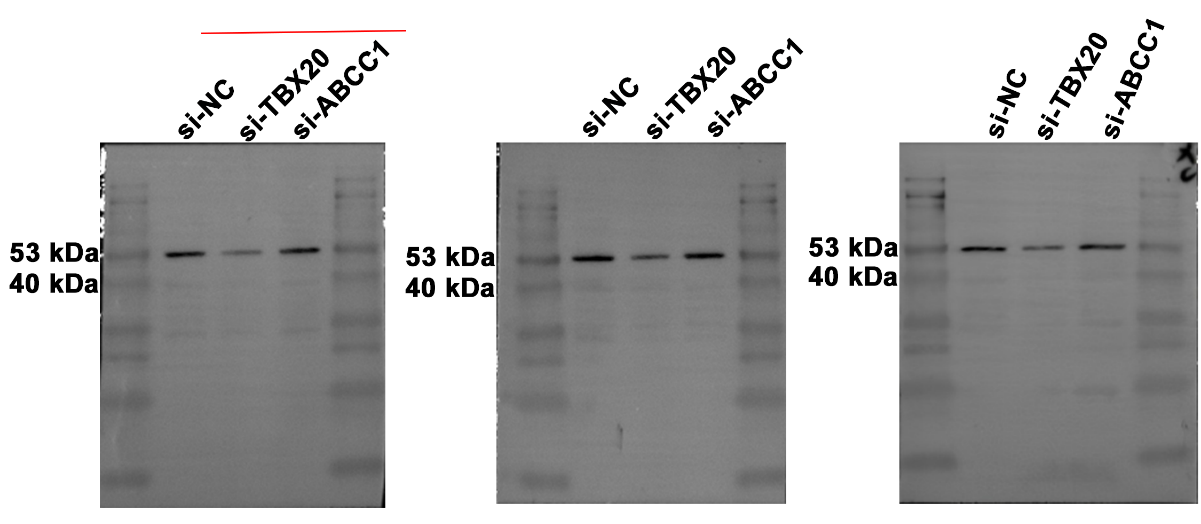


Figure 7E, MDA-MB-231: GAPDH: 37 KDa


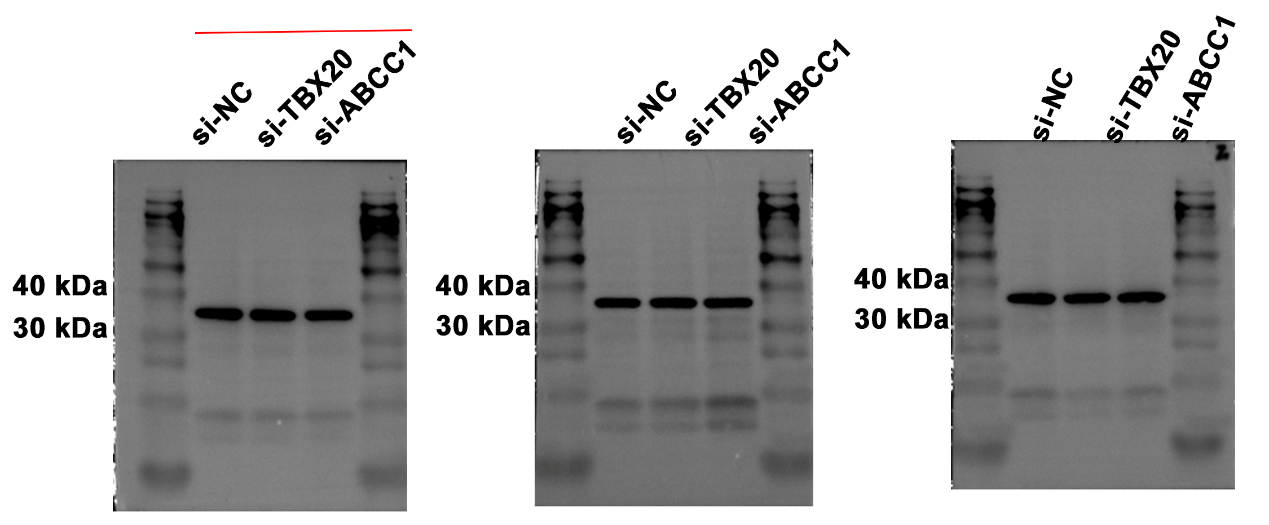


Figure 7F, MDA-MB-231: LC3: 16 KDa, 14 KDa


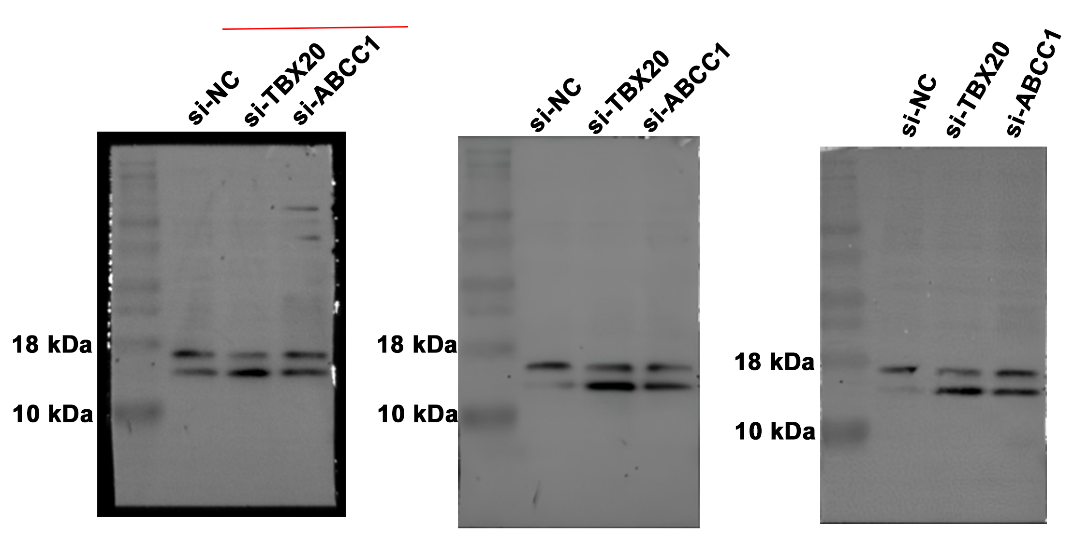


Figure 7F, MDA-MB-231: BNIP3: 30 KDa

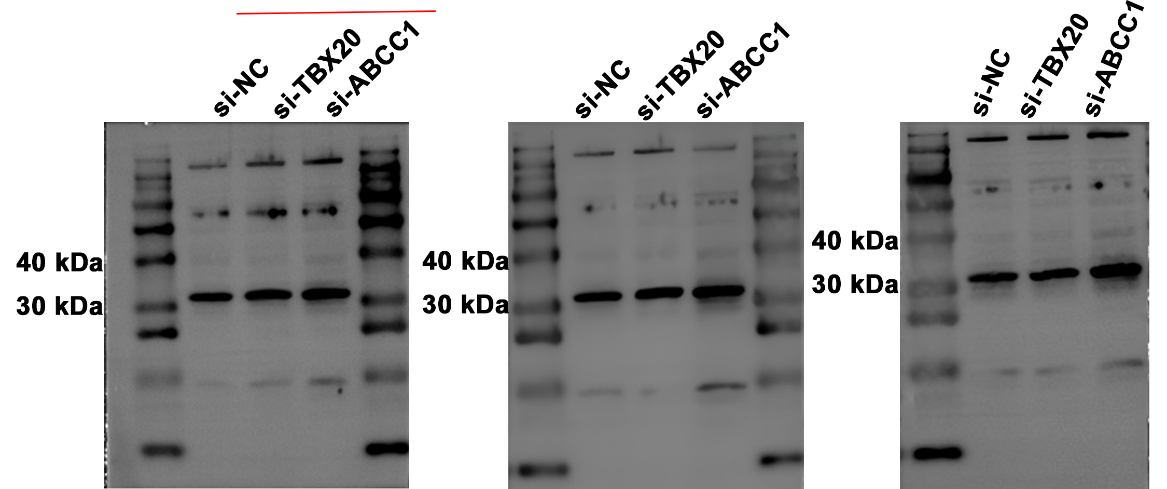


Figure 7F, MDA-MB-231: GAPDH: 37 KDa


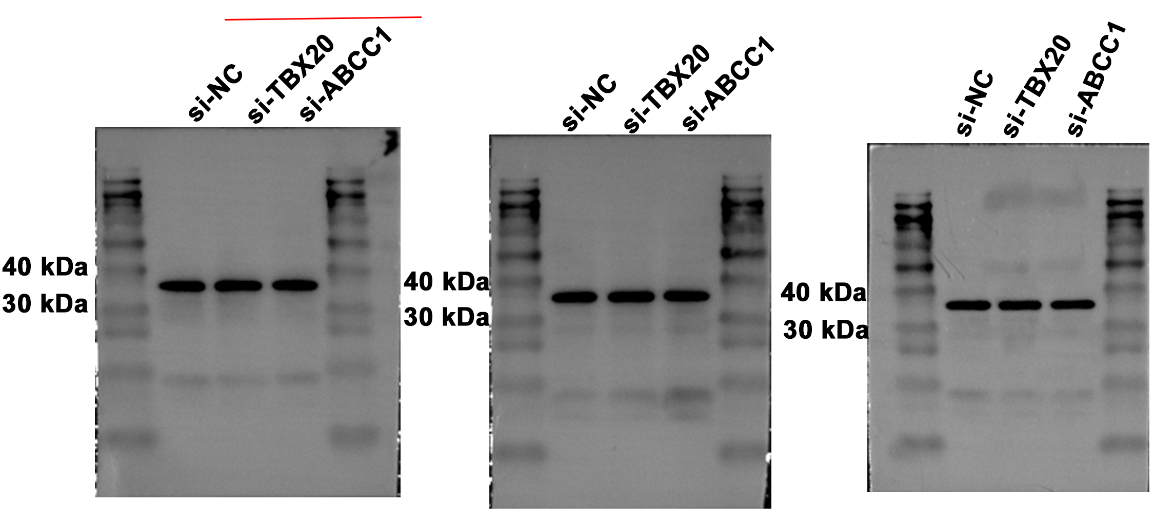


Figure 7F, MDA-MB-231: P62: 62 KDa


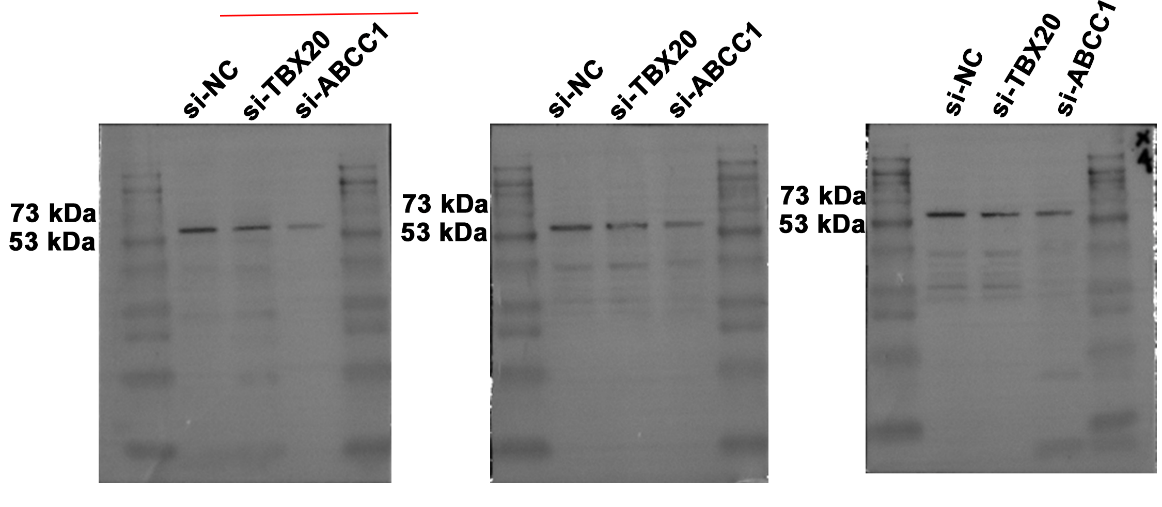


Figure 7F, MDA-MB-231: PINK1: 63 KDa


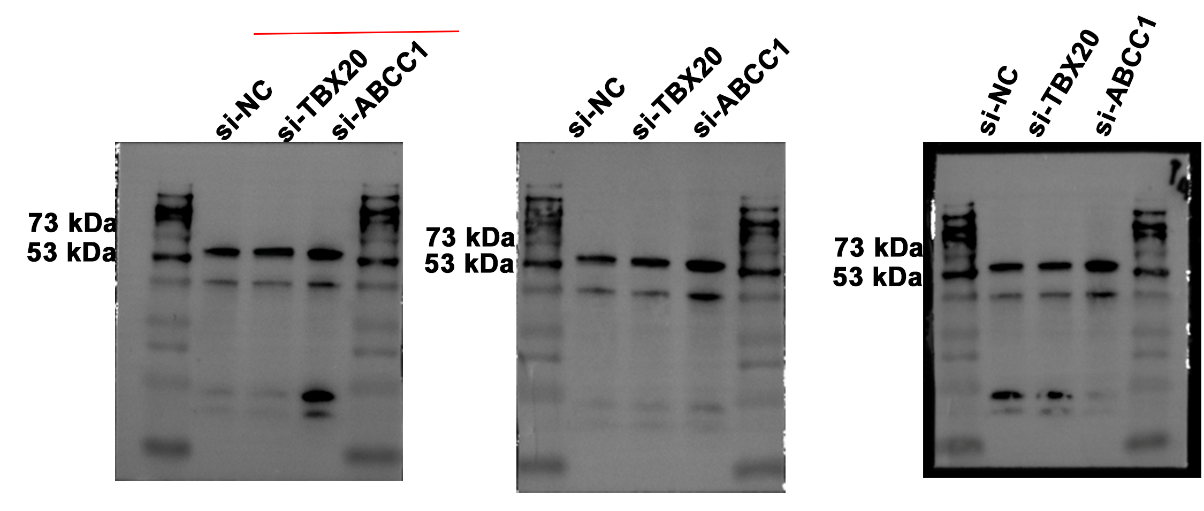

Supplement: S1 File — (DOCX) [file pone.0353376.s001.docx]
