# Supplementary material for: TBX20 promotes doxorubicin resistance in breast cancer cells through enhanced ABCC1 expression and inhibition of mitophagy
Source: PLoS One. 2026 Jul 17;21(7):e0353376. doi: 10.1371/journal.pone.0353376 (PMC13379107; doi:10.1371/journal.pone.0353376)
Supplement: Attachment — Submitted filename: Response_to_Reviewers_auresp_2.docx [file pone.0353376.s005.docx]

Reviewer #1: The authors have addressed the concern by using TBX20 and ABCC1 siRNAs to verify the functional link between TBX20 and ABCC1 in drug resistance. However, additional Western blot data should be provided to determine whether knockdown of TBX20 or ABCC1 affects mitophagy-related markers (e.g., LC3-II, PINK1, et al). This is particularly important if the authors maintain the claim that TBX20 promotes doxorubicin resistance in breast cancer cells through enhanced ABCC1 expression and inhibition of mitophagy.

**Response:** We sincerely thank Reviewer for the insightful comment regarding the need for additional mechanistic evidence linking TBX20, ABCC1, and mitophagy. We agree that providing Western blot data on mitophagy markers following gene knockdown is essential to substantiate our claim. In the revised manuscript, we have supplemented the Western blot results regarding autophagy-related markers to Figure 7 and the corresponding text has been added to the Results section. The detailed experimental results are described below:

In both MCF-7 and MDA-MB-231 cells, qPCR analysis revealed that the mRNA expression levels of ABCC1and TBX20were significantly downregulated in the si-TBX20 and si-ABCC1 groups compared with the si-NC group (*P*< 0.001). Notably, TBX20mRNA expression was significantly upregulated in the si-ABCC1 group (*P*< 0.001) (Fig. 7A and 7D). Consistent with these findings, Western blot assays showed that the protein levels of ABCC1 and TBX20 were markedly decreased in both the si-TBX20 and si-ABCC1 groups relative to the si-NC group (*P*< 0.001); however, there was no statistically significant difference in TBX20 protein expression in the si-ABCC1 group (Fig. 7B and 7E). Furthermore, regarding autophagy-related proteins, the ratio of LC3-II/LC3-I was significantly increased, while the expression of p62 was notably reduced in both knockdown groups (*P*< 0.001). Concurrently, the mitophagy-related proteins PINK1 and BNIP3 were also significantly upregulated (*P*< 0.001) (Fig. 7C and 7F).

**Fig. 7 Silencing of ABCC1 or TBX20 alters the expression of autophagy-related proteins. (A)**: qPCR analysis of ABCC1and TBX20 mRNA expression. **(B)** Western blot analysis of ABCC1 and TBX20 protein levels. **(C)** Western blot analysis of autophagy markers (LC3-I/II, p62) and mitophagy-associated proteins (PINK1, BNIP3). **(D)** Relative mRNA expression levels of ABCC1and TBX20 by qPCR. **(E)** Protein expression levels of ABCC1 and TBX20 by Western blotting. **(F)** Protein levels analysis of LC3-I/II, p62, PINK1, and BNIP3 by Western blotting. Data are presented as mean ± SD (n=3); ****P*< 0.001 compared with the si-NC group, ns: no significant difference.

In addition, could the authors clarify why LC3-II levels are elevated under basal conditions (Figure 5B)? This observation appears inconsistent with the confocal images (Figure 5A), which show no detectable LC3 puncta at baseline. Typically, increased LC3-II levels correlate with the formation of LC3 puncta in immunofluorescence staining.

**Response:** We appreciate the reviewer’s careful observation. We would like to clarify that LC3-II levels are not elevated relative to the control groups; rather, they are significantly decreased in TBX20‑overexpressing cells compared with the NC and CON groups (Fig. 5B). The apparent confusion likely stems from the direction of comparison. In our study, TBX20 overexpression suppresses autophagosome formation, leading to reduced LC3‑II levels, which is consistent with the markedly diminished LC3 puncta observed in confocal imaging (Fig. 5A). Therefore, the Western blot and immunofluorescence data are fully aligned: both indicate that TBX20 overexpression reduces LC3‑II and LC3 puncta relative to baseline controls, rather than increasing them.

Reviewer #2: Author responded to my initial comment about role of TBX20 in modifying doxorubicin sensitivity via ABCC1 and mitophagy that they did a rescue experiment and they compared the doxorubicin sensitivity between cells treated with CCCP alone (not TBX20 alone) and cells with TBX20 + CCCP; and compared siABCC1 alone with TBX20 overexpression+ siABCC1 in figure 5 and 6, however the figures and text don’t contain these new results, they are the same as the data in the original manuscript. In addition, figure 5 is A, B, and C, while its legend is A, B, C, and D. check out consistency between figure body and legend.

- The revised manuscript doesn’t contain figure 1 (only the figure body not exist, you may forgot to submit it)

**Response:** We sincerely thank the reviewer for the insightful comments and suggestions. In the revised manuscript, we have made the following corrections and supplements regarding Figures 5 and 6:

Correction of Figure 5: Due to an oversight in the previous version, the textual description and figure legend of Figure 5 did not match the final figure content. We have now updated both the results description and the legend for Figure 5 to ensure accuracy and consistency.

Supplementation of Figure 6: We have included the rescue experiment data in Figure 6E, demonstrating that knockdown of ABCC1 reverses the doxorubicin resistance induced by TBX20 overexpression. The corresponding image files have also been uploaded to the submission system.

we have supplemented the Western blot results regarding autophagy-related markers to Figure 7 and the corresponding text has been added to the Results section.

We apologize for the omission. The missing Figure 1 has now been uploaded to the system. The details of the revisions are provided below.

**Results Section**

**For Figure 5:** In confocal microscopy observation, the localization of LC3 (green) and TOM20 (red) in MCF-7 and MDA-MB-231 cells with TBX20 overexpression showed changes in the spatial pattern and fluorescence intensity compared to the NC and CON groups (*P* < 0.001), the TBX20 group exhibited markedly diminished LC3 puncta and reduced green fluorescence intensity (Fig. 5A). The Western blot results showed that the LC3-II/LC3-I ratio in MCF-7 and MDA-MB-231 cells with TBX20 overexpression significantly decreased compared with the NC group, and the expression of PINK1 and BNIP3 was also reduced. Notably, the expression of P62 was elevated (*P* < 0.001) (Fig. 5B). The analysis of cell inhibition rate treated with CCCP showed that the IC_50_ values of the TBX20+CCCP group were significantly lower than those of the TBX20 group in both cell lines, suggesting that TBX20 may affect cell sensitivity to drugs by regulating autophagy (Fig. 5C).

**Fig. 5 Overexpression of TBX20 inhibits mitophagy in breast cancer cells. (A)** Immunofluorescence staining of mitochondria (TOM20, red), autophagosomes (LC3, green), and nuclei (DAPI, blue) in MCF-7 and MDA-MB-231 cells. Representative merged images show the spatial distribution of LC3 and TOM20 signals. Yellow regions in the merged channels visually indicate areas of signal overlap, which were qualitatively assessed. Representative images were acquired at 1500× magnification; insets show enlarged regions at 5000X magnification. Scale bars: 20 μm (1500×) and 5 μm (5000×). **(B)** Western blot analysis of mitophagy-related proteins (LC3-II/I, PINK1, BNIP3, and p62) in MCF-7 and MDA-MB-231 cells transfected with TBX20-overexpressing or control vectors. **(C)** CCK-8 assay measuring IC_50_ values of doxorubicin in MCF-7 and MDA-MB-231 cells following treatment with the mitophagy inducer CCCP (10 μM, 24 h). IC_50_ values were calculated using nonlinear regression analysis. Data are presented as mean ± SD (n=3); *** *P* <0.001, TBX20 group vs NC group. CON: Untreated control cells. NC: Negative control; TBX20: Overexpression of TBX20; ADR: Adriamycin; IC_50_: Half maximal inhibitory concentration.

**For Figure 6:** In MCF-7 and MDA-MB-231 cells, TBX20 treatment significantly upregulated ABCC1 mRNA expression, with a notable increase in ABCC1 mRNA in the TBX20-treated group compared to the NC groups (P < 0.001) (Fig. 6A). Western blot analysis showed that ABCC1 protein expression was significantly elevated in the TBX20 group, with higher levels of ABCC1 protein expression compared to the NC groups (P < 0.001) (Fig. 6B). RT-PCR and western blot analyses were conducted to evaluate the silencing efficiency of two ABCC1-targeting siRNAs (si-ABCC1#1 and si-ABCC1#2). Both siRNAs effectively knocked down ABCC1 expression, as evidenced by decreased mRNA (Fig. 6C) and protein (Fig. 6D) levels. Dose-response curve analysis demonstrated that the inhibition curve for the TBX20 + si-NC group was positioned lowest, indicating the poorest sensitivity to doxorubicin and the highest IC50 value. In contrast, the TBX20 + si-ABCC1 #1 and TBX20 + si-ABCC1 #2 groups exhibited significantly higher inhibition rates and markedly reduced IC50 values compared to the control group. Furthermore, both si-ABCC1 #1 and #2 groups alone also displayed enhanced drug sensitivity and lower IC_50_, suggesting that silencing ABCC1effectively potentiates the cytotoxic effects of doxorubicin (Fig. 6E).

**Fig. 6** **TBX20 enhances doxorubicin resistance in breast cancer cells by increasing ABCC1 expression**. **(A)** Relative mRNA expression of ABCC1 in MCF-7 and MDA-MB-231 cells transfected with TBX20-overexpressing or control vectors, determined by qPCR. GAPDH was used as an internal control. **(B)** Western blot analysis confirming ABCC1 protein expression in MCF-7 and MDA-MB-231 cells following TBX20 overexpression. **(C)** Relative mRNA expression of ABCC1 in MCF-7 and MDA-MB-231 cells transfected with si-ABCC1, as determined by qPCR. **(D)** Western blot analysis confirming ABCC1 protein expression in MCF-7 and MDA-MB-231 cells. GAPDH was used as a loading control. **(E)** CCK-8 assay measuring IC_50_ values of doxorubicin in MCF-7 and MDA-MB-231 cells after co-transduction with TBX20 and si-ABCC1. IC_50_ values were determined via nonlinear regression analysis. Data are presented as mean ± SD (n=3); *** *P* <0.001. TBX20: TBX20 overexpression with negative control siRNA; TBX20 + si-ABCC1: TBX20 overexpression with

ABCC1-siRNA co-transduction.

**For Figure 7:** In both MCF-7 and MDA-MB-231 cells, qPCR analysis revealed that the mRNA expression levels of ABCC1and TBX20were significantly downregulated in the si-TBX20 and si-ABCC1 groups compared with the si-NC group (*P*< 0.001) (Fig. 7A and 7D). Consistent with these findings, Western blot assays showed that the protein levels of ABCC1 and TBX20 were markedly decreased in both the si-TBX20 and si-ABCC1 groups relative to the si-NC group (*P*< 0.001) (Fig. 7B and 7E). Furthermore, regarding autophagy-related proteins, the ratio of LC3-II/LC3-I was significantly increased, while the expression of p62 was notably reduced in both knockdown groups (*P*< 0.001). Concurrently, the mitophagy-related proteins PINK1 and BNIP3 were also significantly upregulated (*P*< 0.001) (Fig. 7C and 7F).

**Fig. 7 Silencing of ABCC1 or TBX20 alters the expression of autophagy-related proteins. (A)**: qPCR analysis of ABCC1and TBX20 mRNA expression. **(B)** Western blot analysis of ABCC1 and TBX20 protein levels. **(C)** Western blot analysis of autophagy markers (LC3-I/II, p62) and mitophagy-associated proteins (PINK1, BNIP3). **(D)** Relative mRNA expression levels of ABCC1and TBX20 by qPCR. **(E)** Protein expression levels of ABCC1 and TBX20 by Western blotting. **(F)** Protein levels analysis of LC3-I/II, p62, PINK1, and BNIP3 by Western blotting. Data are presented as mean ± SD (n=3); ****P*< 0.001 compared with the si-NC group, ns: no significant difference. The si-TBX20 group refers to cells transfected with the TBX20 siRNA duplex si-TBX20#1, and the si-ABCC1 group refers to cells transfected with the ABCC1siRNA duplex si-ABCC1#1. The si-NC group indicates cells transfected with a non-targeting negative control siRNA
