# Supplementary material for: TBX20 promotes doxorubicin resistance in breast cancer cells through enhanced ABCC1 expression and inhibition of mitophagy
Source: PLoS One. 2026 Jul 17;21(7):e0353376. doi: 10.1371/journal.pone.0353376 (PMC13379107; doi:10.1371/journal.pone.0353376)
Supplement: Attachment — Submitted filename: Review comments.docx [file pone.0353376.s002.docx]

This work highlighted the role of TBX20 in regulation ABCC1 and drug resistance in breast cancer, which is good. However, the manuscript has some concerns regarding consistency of data interpretation and consistency

**Major comments**

*Concept*

- The hypothesis of the author is based on that TBX20 induce drug resistance by inhibiting mitophagy. And this contradict the well-know and documented fact that mitophagy leads to drug resistance and , subsequently, its inhibition and targeting is a mechanism to decrease and overcome drug resistance, not induce it
- in the introduction author mentioned that “*This study will delve into the role of TBX20 in breast cancer and its mechanism in promoting ADR resistance by inhibiting mitochondrial autophagy through ABCC1*” , this sentence means that ABCC1 mediates the TBX20-mediated autophagy, which means that ABCC1 is directly involved in autophagy process itself. This role is never determined for ABCC1 before, no mechanistic validation done for this conclusion, neither by the author , nor by previous publication. The work of the authors linked between TBX20 and mitophagy, and between TBX20 and ABCC1 separately, abut **not** an axis TBX20/ABCC1/mitophagy.

*Other major concerns*

- -. In results part, author mentioned that TBX20 expression inhibit mitophagy and proofed that by increasing the LC3-II/LC3-I ratio in cells with enforced TBX20 expression compared to control, how come???. generally, in mitophagy the ratio of LC3-II/LC3-I increase, this means when you do a transduction that inhibit mitophagy it should leads to decrease, not increase of of LC3-II/LC3-I ratio. And in discussion author mentioned the ratio is decreased, so what written in results is not the same as in discussion.
- - the same in case of increasing the expression of P62, PINK1, and BNIP3 with compared to the NC and control, all of them are indicators of mitophagy, so if mitophagy inhibited, they should decrease, not increase as author mentioned in the text of results, which is not the same as mentioned in discussion. And the data in the figure 4 not matched with the text of results. in western blot figure, only p62 increase, while PINK1, and BNIP3 decrease. also LC3II is the one with 14 kd which showed decrease not increase in western image. Author need to reconsider data interpretation and drafting and avoid inconsistency

- To validate that TBX20 regulate drug resistance via inhibition of mitophagy, author compared IC50 for doxorubicin in cell with TBX20 expression vs cells with TBX20 expression+ mitophagy inducer. The correct to validate the role of TBX20 in mitophagy, you should compare ic50 in cells with mitophagy inducer vs. cells with TBX20+ mitophagy inducer, and you should test the mitophagy phenotype to confirm this. and again, the inhibition of mitophagy should reduce drug resistance, not the increase it.
- Same thing in case of validating the role of TBX20 in regulating drug resistance via ABCC1. author compared ic50 for doxorubicin in cell with TBX20 expression vs. cells with TBX20+ siABCC1. While you should compare IC50 in cells with siABCC1 vs. cells with TBX20+ siABCC1 to test the ability of TBX20 to restore the effect of siABCC1
- - data of IF need more explanation in text part of results and discussion. Also in legend of figure 4 of IF, author used the merged LC3 and TOM20 to conclude their colocalization. Which is not correct, because standard merged immunofluorescence (IF) is **not sufficient** on its own to *validate* colocalization because visual overlap (yellow color) can be misleading, often depending heavily on probe brightness and potentially masking true proximity or creating false positives. Colocalization requires other techniques not involved here, so you cant conclude they are colocalized.

**Minor comment:**

- the r value of sperman correlation is 0.243 revealed weak correlation between TBX20 and ABCC1

-in the second paragraph of results titled ‘Overexpression of TBX20 promotes doxorubicin resistance in breast cancer cell”:

* there is an error of figure citation, author cite figure in this paragraph as figure 1, while the representing figure is figure 2.

* the paragraph need rewriting to elaborate that the cell line are transfected, because the way it written in is confusing and look like the results are for naïve cell line. Elaboration is also required for CON and NC when they are written for the first time. And values representing the results (such as p value) should be mentioned in the text, not only in the legend of the figure

*treatment of cells with lentivirus for gene expression is called transduction, not transfection

- treatment with Amphotericin B and cccp not mentioned in methodology. Need explanation.
