# Supplementary material for: TBX20 promotes doxorubicin resistance in breast cancer cells through enhanced ABCC1 expression and inhibition of mitophagy
Source: PLoS One. 2026 Jul 17;21(7):e0353376. doi: 10.1371/journal.pone.0353376 (PMC13379107; doi:10.1371/journal.pone.0353376)
Supplement: Attachment — Submitted filename: Response to reviewers.docx [file pone.0353376.s003.docx]

Response to reviewers

Manuscript ID: PONE-D-25-64318

Title: TBX20 promotes doxorubicin resistance in breast cancer cells through enhanced ABCC1 expression and inhibition of mitophagy

Journal: PLOS ONE

Dear Dr. Peng Zhang and Reviewers,

Thank you for giving us the opportunity to revise our manuscript. We sincerely appreciate the time and effort that you and the reviewers have dedicated to providing insightful and constructive comments. We are grateful for the valuable feedback, which has helped us substantially improve the quality and clarity of our work.

We have carefully addressed all the points raised by the academic editor and reviewers. A copy of the revised manuscript with all changes highlighted using the “Track Changes” function in Microsoft Word has been uploaded as a separate file. The specific descriptions of the revisions are provided point-by-point below.

**Reviewer #1:** This manuscript investigates the role of TBX20 in doxorubicin resistance in breast cancer cells and proposes a mechanism involving enhanced ABCC1 expression and inhibition of mitophagy. The topic is clinically relevant, as chemoresistance remains a major challenge in breast cancer treatment and identifying novel regulatory factors may provide new therapeutic opportunities. The study presents several strengths. The authors combine bioinformatics analysis of TCGA datasets with in vitro functional experiments in two breast cancer cell lines, which strengthens the biological relevance of their findings. The use of multiple assays to assess proliferation, migration, drug resistance, and mitophagy provides a relatively comprehensive evaluation of TBX20 function. The observation that ABCC1 silencing reverses doxorubicin resistance supports a functional link between TBX20 and drug efflux mechanisms.

However, several issues limit the impact of the study. First, the reliance on TBX20 overexpression without complementary loss-of-function experiments (e.g., TBX20 knockdown) weakens the causal interpretation of TBX20’s role in chemoresistance.For knockdown of ABCC1, at least two siRNAs could be appiled to rule out off-target effects. Second, the mechanistic connection between TBX20 and ABCC1 regulation is not fully established; it remains unclear whether TBX20 directly regulates ABCC1 transcription or acts indirectly through other pathways. Third, the assessment of mitophagy is based mainly on marker expression (LC3, P62, PINK1, and BNIP3), which may not be sufficient to conclusively demonstrate changes in mitophagic flux. Additional functional or dynamic assays would strengthen this conclusion. Finally, the absence of in vivo validation limits the translational significance of the findings. In summary, the manuscript addresses an important question and provides suggestive evidence that TBX20 contributes to doxorubicin resistance in breast cancer cells. With additional mechanistic validation, inclusion of loss-of-function studies, and stronger mitophagy assays, the study could be significantly strengthened and make a more substantial contribution to the field.

**Response:**

Thank you for the reviewer's constructive comments. We have made important additions and revisions to the study and the manuscript in accordance with your suggestions. Our detailed responses are as follows:

①Supplementation of TBX20 loss-of-function experiments: The results indicate that silencing TBX20 leads to reduced ABCC1 expression and partially reverses doxorubicin resistance, thereby corroborating the findings from overexpression experiments and strengthening the causal role of TBX20 in chemotherapy resistance (see figures 3).

②Enhanced rigor of ABCC1 silencing experiments: We have included silencing efficiency data for ABCC1 using two independent siRNAs (see figures 6).

③Elucidation of the direct regulatory mechanism of TBX20 on ABCC1: Additional experiments have been conducted, including chromatin immunoprecipitation assays, which confirm that the TBX20 protein directly binds to the promoter region of the ABCC1 gene. Dual-luciferase reporter assays further demonstrate that TBX20 activates ABCC1 promoter activity (see figures 7).

④Strengthened functional validation of mitophagy: Our research is not only based on marker expression (LC3, P62, PINK1, and BNIP3) but also includes immunofluorescence to observe mitophagy levels using LC-3 and TOM20. This provides a visual assessment of mitophagy. Of course, further observations of mitophagy require additional techniques, such as electron microscopy and dynamic monitoring using LC-3 dual-fluorescence autophagy flux markers. However, given the current experimental conditions, these approaches are not feasible at this stage. We have acknowledged these limitations in the discussion section.

⑤Clarification regarding in vivo experiments: We fully understand and agree with the reviewer’s perspective on the importance of in vivo experiments for translational relevance. It is important to note that the focus of this study is to elucidate, for the first time at the cellular and molecular levels, a novel mechanism by which TBX20 mediates doxorubicin resistance in breast cancer through direct transcriptional regulation of ABCC1 and inhibition of mitophagy. The rigorous in vitro gain- and loss-of-function experiments, along with the mechanistic exploration conducted in this study, provide a solid theoretical foundation for TBX20 as a potential driver and therapeutic target in breast cancer chemoresistance. While the lack of in vivo experimental data is a limitation of the current study, we have explicitly acknowledged this point in the discussion section and emphasized its importance for future research.

**Reviewer #2:** This work highlighted the role of TBX20 in regulation ABCC1 and drug resistance in breast cancer, which is good. However, the manuscript has some concerns regarding consistency of data interpretation and consistency.

Major comments Concept

-The hypothesis of the author is based on that TBX20 induce drug resistance by inhibiting mitophagy. And this contradict the well-know and documented fact that mitophagy leads to drug resistance and , subsequently, its inhibition and targeting is a mechanism to decrease and overcome drug resistance, not induce it- in the introduction author mentioned that “This study will delve into the role of TBX20 in breast cancer and its mechanism in promoting ADR resistance by inhibiting mitochondrial autophagy through ABCC1” , this sentence means that ABCC1 mediates the TBX20-mediated autophagy, which means that ABCC1 is directly involved in autophagy process itself. This role is never determined for ABCC1 before, no mechanistic validation done for this conclusion, neither by the author, nor by previous publication. The work of the authors linked between TBX20 and mitophagy, and between TBX20 and ABCC1 separately, abut not an axis TBX20/ABCC1/mitophagy.

**Response:** We sincerely thank the reviewer for this insightful and critical comment, which allows us to clarify a key and novel aspect of our findings. We fully acknowledge the well-established paradigm that chemotherapy-induced mitophagy often acts as a cytoprotective mechanism, contributing to therapy resistance. Our data, however, appear to reveal a different, context-dependent role of mitophagy under the specific drive of TBX20.

①We agree that the observation—inhibition of mitophagy correlating with increased drug resistance—seems counterintuitive within the classic framework. To address this point, we have substantially revised the Discussion section. We now propose that TBX20 may not be modulating a “therapy-induced” mitophagy but rather suppressing basal or homeostatic mitophagic flux. This chronic suppression could lead to the accumulation of dysfunctional mitochondria, creating a state of sustained mitochondrial stress. In response, cells might activate robust compensatory survival pathways (such as the upregulation of the drug efflux pump ABCC1, as observed in our study) to adapt, ultimately resulting in a drug-resistant phenotype. This concept is supported by emerging literature showing that genetic or functional deficiencies in core mitophagy components (e.g., ULK1, ARL3) can promote tumor progression and therapy resistance in specific contexts [cited as ref. 21 & 22 in our revised manuscript]. Thus, our findings do not contradict but rather complement the existing knowledge by highlighting the “double-edged sword” nature of mitophagy in cancer. We have rewritten the relevant sections to present our hypothesis within this more nuanced and evolving theoretical framework.

We greatly appreciate the reviewer for pointing out this overstatement and lack of clarity in our original introduction. The reviewer is absolutely correct. We have no experimental evidence to support a direct role of ABCC1 in mediating mitophagy, and such a role has not been established in the literature. The sentence in question was an unintended and inaccurate synthesis of two separate observations.We have therefore made the following key revisions to the manuscript:

Introduction: The problematic sentence has been completely removed. The concluding paragraph of the introduction now states our aims more precisely and separately: “This study aims to... examining whether TBX20 promotes chemoresistance through the transcriptional upregulation of the ABCC1 and/or via the modulation of mitochondrial autophagy.”

Results & Discussion: Throughout the text, we now clearly present the upregulation of ABCC1 and the suppression of mitophagy as two distinct, important downstream consequences of TBX20 overexpression. We have replaced causal language (e.g., “through ABCC1”) with associative and correlative language (e.g., “is associated with,” “accompanied by”) to accurately reflect our data. We explicitly state in the Discussion that the mechanistic connection between ABCC1 upregulation and mitophagy suppression remains an open question and a valuable direction for future research.

②Regarding the construction of the “TBX20/ABCC1/Mitophagy” regulatory axis:

Clarifying the experimentally confirmed direct regulatory relationship: We emphasize the new experimental evidence, confirmed by chromatin immunoprecipitation and dual-luciferase reporter assays, that TBX20 directly transcriptionally upregulates ABCC1, and we clearly state that this is one of the core findings of this study.

Revising the functional role of ABCC1: We now define ABCC1 as a key downstream effector of TBX20, responsible for mediating TBX20-induced resistance to doxorubicin. This is strongly supported by our data (TBX20 upregulates ABCC1, and ABCC1 knockdown reverses TBX20-mediated resistance).

Clarifying the relationship between ABCC1 and mitophagy: We explicitly state that it remains unknown whether and how ABCC1 directly participates in the mitophagy process. Our data indicate that TBX20 upregulation of ABCC1 is associated with changes in mitophagy markers. However, the two may be parallel or co-downstream events, or they may be indirectly linked through mechanisms yet to be elucidated. We propose that ABCC1 may indirectly influence mitochondrial homeostasis—and thereby the status of mitophagy—through its drug efflux function, which alters the intracellular environment, or through other uncharacterized functions. We have revised the conclusion from a direct causal chain to a description of the observed association and have identified elucidating the precise mechanism as an important direction for future research. This limitation is also clearly noted in the discussion.

Other major concerns

- In results part, author mentioned that TBX20 expression inhibit mitophagy and proofed that by increasing the LC3-II/LC3-I ratio in cells with enforced TBX20 expression compared to control, how come? generally, in mitophagy the ratio of LC3-II/LC3-I increase, this means when you do a transduction that inhibit mitophagy it should leads to decrease, not increase of of LC3-II/LC3-I ratio. And in discussion author mentioned the ratio is decreased, so what written in results is not the same as in discussion.

- the same in case of increasing the expression of P62, PINK1, and BNIP3 with compared to the NC and control, all of them are indicators of mitophagy, so if mitophagy inhibited, they should decrease, not increase as author mentioned in the text of results, which is not the same as mentioned in discussion. And the data in the figure 4 not matched with the text of results. in western blot figure, only p62 increase, while PINK1, and BNIP3 decrease. Also LC3II is the one with 14 kd which showed decrease not increase in western image. Author need to reconsider data interpretation and drafting and avoid inconsistency.

**Response:** Thank you for the reviewer's meticulous and professional feedback. The inconsistencies you highlighted between the text description, the data figures, and the conclusions are of critical importance. We have conducted a thorough review and made corresponding revisions to the manuscript. Our detailed responses are as follows:

①We confirmed that the Western blot results in Figure 4B indeed show that, compared to the control group, the TBX20 overexpression group exhibited a decreased LC3-II/LC3-I ratio, as well as reduced expression of PINK1 and BNIP3 proteins. The phrase “significantly increased” in the original text was a serious clerical error. In the Results section, we have now corrected this to “significantly decreased” and “reduced” to ensure that the textual description perfectly matches the graphical data and supports the conclusion of “inhibited mitophagy.”

②Regarding a separate clarification on the P62 data: Your observation that the trend of P62 in the figure is inconsistent with that of PINK1/BNIP3 is very accurate. P62 is a multifunctional adaptor protein, and its accumulation is typically associated with the inhibition of autophagic flux (including mitophagy). To describe the data more precisely and avoid confusion, we have separately noted the trend of P62 in the revision, stating, "Notably, the expression of the autophagy receptor protein P62 was elevated." This formulation remains faithful to the Western blot results (which show an intensified P62 band) and aligns with the classic interpretation that "inhibition of autophagy/mitophagy leads to P62 accumulation." This resolves the apparent contradiction between the text and the figure and ensures consistency with the overall conclusions.

③Regarding consistency between the Results and Discussion sections: We have also cross-checked the Discussion section to ensure that all references to the results are consistent with the revised descriptions in the Results section. The discussion now clearly supports the finding that “TBX20 inhibits mitophagy,” thereby addressing the inconsistency you pointed out between different sections of the text.

④Regarding the figure citation numbering: We have corrected the figure numbers cited in the text to ensure they accurately correspond to the figure labels in the manuscript.

- To validate that TBX20 regulate drug resistance via inhibition of mitophagy, author compared IC50 for doxorubicin in cell with TBX20 expression vs cells with TBX20 expression+ mitophagy inducer. The correct to validate the role of TBX20 in mitophagy, you should compare ic50 in cells with mitophagy inducer vs. cells with TBX20+ mitophagy inducer, and you should test the mitophagy phenotype to confirm this. and again, the inhibition of mitophagy should reduce drug resistance, not the increase it.

- Same thing in case of validating the role of TBX20 in regulating drug resistance via ABCC1. author compared ic50 for doxorubicin in cell with TBX20 expression vs. cells with TBX20+ siABCC1. While you should compare IC50 in cells with siABCC1 vs. cells with TBX20+ siABCC1 to test the ability of TBX20 to restore the effect of siABCC1.

**Response:**

We have performed the following additional experiments as suggested:

Comparative IC50 Analysis: We compared the doxorubicin sensitivity between cells treated with CCCP alone and cells with TBX20 + CCCP. The results, now included in the revised manuscript (Fig. 4D), show that while CCCP treatment alone significantly sensitizes control cells to doxorubicin, this sensitizing effect is markedly blunted in TBX20-overexpressing cells. The IC50 of the TBX20+CCCP group is significantly higher than that of the CCCP group, indicating that TBX20 overexpression counteracts the chemosensitizing effect of induced mitophagy. Our finding—that inhibiting mitophagy conversely promotes drug resistance—indeed challenges the established paradigm. We believe this precisely reveals the distinctive role of TBX20: it may not regulate acute therapy-induced autophagy but rather continuously suppresses basal mitophagy. This persistent inhibition leads to the accumulation of damaged mitochondria, triggering sustained cellular stress, which may activate robust compensatory survival programs exemplified by the upregulation of ABCC1. Consequently, the net effect manifests as enhanced drug resistance. This aligns with emerging perspectives from recent studies (such as those reporting on ULK1 and ARL3 deficiencies) [cited as ref. 21 & 22 in our revised manuscript], which suggest that “mitophagy defects can promote cancer progression in certain contexts.” We have now more clearly elaborated this context-dependent model in the Discussion section.

As suggested, we have performed the recommended rescue experiment. The results, now included in the revised manuscript (Fig. 5 and corresponding text in the Results section), clearly show that: 1) Silencing ABCC1 alone sensitizes cells to doxorubicin; and 2) Ectopic overexpression of TBX20 in the ABCC1-silenced background effectively restores the doxorubicin resistance phenotype. This rescue experiment provides direct functional evidence that TBX20 acts, at least in part, through ABCC1 to promote chemoresistance. We believe this addition has substantially strengthened our conclusion regarding the TBX20/ABCC1 axis.

- data of IF need more explanation in text part of results and discussion. Also in legend of figure 4 of IF, author used the merged LC3 and TOM20 to conclude their colocalization. Which is not correct, because standard merged immunofluorescence (IF) is not sufficient on its own to validate colocalization because visual overlap (yellow color) can be misleading, often depending heavily on probe brightness and potentially masking true proximity or creating false positives. Colocalization requires other techniques not involved here, so you can’t conclude they are colocalized.

**Response:** Thank you for the reviewer's valuable comments. Your reminder regarding the rigor of the immunofluorescence (IF) data analysis is highly pertinent. In accordance with your suggestions, we have revised the descriptions of the IF data in the manuscript. In the results section, we have entirely removed explicit conclusions regarding “co-localization.” The descriptions have been amended to instead discuss the observed spatial distribution patterns and changes in fluorescence intensity for LC3 and TOM20.

Minor comment:

- the r value of sperman correlation is 0.243 revealed weak correlation between TBX20 and ABCC1.

-in the second paragraph of results titled ‘Overexpression of TBX20 promotes doxorubicin resistance in breast cancer cell”:

* there is an error of figure citation, author cite figure in this paragraph as figure 1, while the representing figure is figure 2.

* the paragraph need rewriting to elaborate that the cell line are transfected, because the way it written in is confusing and look like the results are for naïve cell line. Elaboration is also required for CON and NC when they are written for the first time. And values representing the results (such as p value) should be mentioned in the text, not only in the legend of the figure.

*treatment of cells with lentivirus for gene expression is called transduction, not transfection.

- treatment with Amphotericin B and cccp not mentioned in methodology. Need explanation.

**Response:** Thank you for the reviewer’s further valuable and meticulous suggestions. In accordance with each of your comments, we have conducted a comprehensive revision and clarification of the Results section and the corresponding figure legends in the manuscript. The specific changes and our responses are outlined below:

Regarding the weak correlation (R=0.243) between TBX20 and ABCC1: We thank the reviewer for this observation. We agree that the correlation coefficient indicates a weak positive association at the transcriptional level in TCGA datasets. We wish to clarify that this bioinformatic analysis was intended as an initial screen to identify a potential link. The primary evidence supporting the TBX20-ABCC1 regulatory axis in our study comes from the subsequent functional experiments, including chromatin immunoprecipitation, dual-luciferase reporter assays, and the rescue experiments, which demonstrate a direct and functional relationship.

Regarding errors and clarifications in the second results paragraph:

Figure citation error: We sincerely apologize for the incorrect figure citations (Fig. 1A2A, etc.). This has been corrected to refer to the proper figures (Fig. 2A, 2B, 2C).

Clarity on cell treatment: We have rewritten the paragraph to explicitly state that the cells were transduced with lentivirus for TBX20 overexpression. The control groups (CON: Untreated control; NC: negative control) are now clearly defined upon their first mention.

Inclusion of statistical values in text: As suggested, the relevant p-values have now been incorporated into the main text description.

We have replaced “transfection” with “transduction”.

Regarding the missing methodology for CCCP and Amphotericin B treatment: We apologize for this omission. The mention of “Amphotericin B” in the result text was a typographical error; the correct compound used was the mitophagy inducer CCCP. We have corrected this in the manuscript. Furthermore, a subsection titled “CCK8 assay” has been the Materials and methods section, detailing the preparation, storage, and application of doxorubicin and CCCP.
