# Supplementary material for: TBX20 promotes doxorubicin resistance in breast cancer cells through enhanced ABCC1 expression and inhibition of mitophagy
Source: PLoS One. 2026 Jul 17;21(7):e0353376. doi: 10.1371/journal.pone.0353376 (PMC13379107; doi:10.1371/journal.pone.0353376)
Supplement: Attachment — Submitted filename: new review comments.docx [file pone.0353376.s004.docx]

The revised manuscript contains good explanations that made the manuscript clearer and more consistent. Authors carried out most of required modifications, however, there is an important experiment they responded by doing it, but it is not included in the manuscript:

- Author responded to my initial comment about role of TBX20 in modifying doxorubicin sensitivity via ABCC1 and mitophagy that they did a rescue experiment and they compared the doxorubicin sensitivity between cells treated with CCCP alone (not TBX20 alone) and cells with TBX20 + CCCP; and compared siABCC1 alone with TBX20 overexpression+ siABCC1 in figure 5 and 6, however the figures and text don’t contain these new results, they are the same as the data in the original manuscript. In addition, figure 5 is A, B, and C, while its legend is A, B, C, and D. check out consistency between figure body and legend.
- The revised manuscript doesn’t contain figure 1 (only the figure body not exist, you may forgot to submit it)
