# Supplementary material for: TBX20 promotes doxorubicin resistance in breast cancer cells through enhanced ABCC1 expression and inhibition of mitophagy
Source: PLoS One. 2026 Jul 17;21(7):e0353376. doi: 10.1371/journal.pone.0353376 (PMC13379107; doi:10.1371/journal.pone.0353376)
Supplement: Peer review history

# Peer review: Decision Letter 0

**DOI:** 10.1371/journal.pone.0353376.r001

**Authors:** Peng Zhang

25 Dec 2025

PONE-D-25-64318

TBX20 promotes doxorubicin resistance in breast cancer cells through enhanced ABCC1 expression and inhibition of mitophagy

PLOS One

Dear Dr. Mi,

Thank you for submitting your manuscript to PLOS ONE. After careful consideration, we feel that it has merit but does not fully meet PLOS ONE’s publication criteria as it currently stands. Therefore, we invite you to submit a revised version of the manuscript that addresses the points raised during the review process.

Please submit your revised manuscript by Feb 08 2026 11:59PM. If you will need more time than this to complete your revisions, please reply to this message or contact the journal office at plosone@plos.org. When you're ready to submit your revision, log on to https://www.editorialmanager.com/pone/ and select the 'Submissions Needing Revision' folder to locate your manuscript file.

Please include the following items when submitting your revised manuscript:

- A letter that responds to each point raised by the academic editor and reviewer(s). You should upload this letter as a separate file labeled 'Response to Reviewers'.
- A marked-up copy of your manuscript that highlights changes made to the original version. You should upload this as a separate file labeled 'Revised Manuscript with Track Changes'.
- An unmarked version of your revised paper without tracked changes. You should upload this as a separate file labeled 'Manuscript'.

If you would like to make changes to your financial disclosure, please include your updated statement in your cover letter. Guidelines for resubmitting your figure files are available below the reviewer comments at the end of this letter.

If applicable, we recommend that you deposit your laboratory protocols in protocols.io to enhance the reproducibility of your results. Protocols.io assigns your protocol its own identifier (DOI) so that it can be cited independently in the future. For instructions see: https://journals.plos.org/plosone/s/submission-guidelines#loc-laboratory-protocols. Additionally, PLOS ONE offers an option for publishing peer-reviewed Lab Protocol articles, which describe protocols hosted on protocols.io. Read more information on sharing protocols at https://plos.org/protocols?utm_medium=editorial-email&utm_source=authorletters&utm_campaign=protocols.

We look forward to receiving your revised manuscript.

Kind regards,

Peng Zhang, Ph.D.

Academic Editor

PLOS One

Journal Requirements:

When submitting your revision, we need you to address these additional requirements.

1. Please ensure that your manuscript meets PLOS ONE's style requirements, including those for file naming. The PLOS ONE style templates can be found at

https://journals.plos.org/plosone/s/file?id=wjVg/PLOSOne_formatting_sample_main_body.pdf and

https://journals.plos.org/plosone/s/file?id=ba62/PLOSOne_formatting_sample_title_authors_affiliations.pdf

2. PLOS ONE now requires that authors provide the original uncropped and unadjusted images underlying all blot or gel results reported in a submission’s figures or Supporting Information files. This policy and the journal’s other requirements for blot/gel reporting and figure preparation are described in detail at https://journals.plos.org/plosone/s/figures#loc-blot-and-gel-reporting-requirements and https://journals.plos.org/plosone/s/figures#loc-preparing-figures-from-image-files. When you submit your revised manuscript, please ensure that your figures adhere fully to these guidelines and provide the original underlying images for all blot or gel data reported in your submission. See the following link for instructions on providing the original image data: https://journals.plos.org/plosone/s/figures#loc-original-images-for-blots-and-gels.

In your cover letter, please note whether your blot/gel image data are in Supporting Information or posted at a public data repository, provide the repository URL if relevant, and provide specific details as to which raw blot/gel images, if any, are not available. Email us at plosone@plos.org if you have any questions.

3. Please note that PLOS One has specific guidelines on code sharing for submissions in which author-generated code underpins the findings in the manuscript. In these cases, we expect all author-generated code to be made available without restrictions upon publication of the work. Please review our guidelines at https://journals.plos.org/plosone/s/materials-and-software-sharing#loc-sharing-code and ensure that your code is shared in a way that follows best practice and facilitates reproducibility and reuse.

4. PLOS requires an ORCID iD for the corresponding author in Editorial Manager on papers submitted after December 6th, 2016. Please ensure that you have an ORCID iD and that it is validated in Editorial Manager. To do this, go to ‘Update my Information’ (in the upper left-hand corner of the main menu), and click on the Fetch/Validate link next to the ORCID field. This will take you to the ORCID site and allow you to create a new iD or authenticate a pre-existing iD in Editorial Manager.

5. Thank you for stating the following financial disclosure:

This work was supported by National Natural Science Foundation of China (31370524, 21377052, and 31670519) and Fundamental Research Funds for the Central Universities (021414380332).

Please state what role the funders took in the study. If the funders had no role, please state: "The funders had no role in study design, data collection and analysis, decision to publish, or preparation of the manuscript."

If this statement is not correct you must amend it as needed.

Please include this amended Role of Funder statement in your cover letter; we will change the online submission form on your behalf.

6. Please ensure that you refer to Figure 2 in your text as, if accepted, production will need this reference to link the reader to the figure.

7. If the reviewer comments include a recommendation to cite specific previously published works, please review and evaluate these publications to determine whether they are relevant and should be cited. There is no requirement to cite these works unless the editor has indicated otherwise.

[Note: HTML markup is below. Please do not edit.]

Reviewers' comments:

Reviewer's Responses to Questions

**Comments to the Author**

1. Is the manuscript technically sound, and do the data support the conclusions?

The manuscript must describe a technically sound piece of scientific research with data that supports the conclusions. Experiments must have been conducted rigorously, with appropriate controls, replication, and sample sizes. The conclusions must be drawn appropriately based on the data presented.

Reviewer #1: Partly

Reviewer #2: Partly

**********

2. Has the statistical analysis been performed appropriately and rigorously?

Reviewer #1: Yes

Reviewer #2: Yes

**********

3. Have the authors made all data underlying the findings in their manuscript fully available?

The PLOS Data policy requires authors to make all data underlying the findings described in their manuscript fully available without restriction, with rare exception (please refer to the Data Availability Statement in the manuscript PDF file). The data should be provided as part of the manuscript or its supporting information, or deposited to a public repository. For example, in addition to summary statistics, the data points behind means, medians and variance measures should be available. If there are restrictions on publicly sharing data—e.g. participant privacy or use of data from a third party—those must be specified.

Reviewer #1: Yes

Reviewer #2: Yes

**********

4. Is the manuscript presented in an intelligible fashion and written in standard English?

PLOS ONE does not copyedit accepted manuscripts, so the language in submitted articles must be clear, correct, and unambiguous. Any typographical or grammatical errors should be corrected at revision, so please note any specific errors here.

Reviewer #1: Yes

Reviewer #2: Yes

**********

5. Review Comments to the Author

Please use the space provided to explain your answers to the questions above. You may also include additional comments for the author, including concerns about dual publication, research ethics, or publication ethics. (Please upload your review as an attachment if it exceeds 20,000 characters)

Reviewer #1: This manuscript investigates the role of TBX20 in doxorubicin resistance in breast cancer cells and proposes a mechanism involving enhanced ABCC1 expression and inhibition of mitophagy. The topic is clinically relevant, as chemoresistance remains a major challenge in breast cancer treatment, and identifying novel regulatory factors may provide new therapeutic opportunities.

The study presents several strengths. The authors combine bioinformatics analysis of TCGA datasets with in vitro functional experiments in two breast cancer cell lines, which strengthens the biological relevance of their findings. The use of multiple assays to assess proliferation, migration, drug resistance, and mitophagy provides a relatively comprehensive evaluation of TBX20 function. The observation that ABCC1 silencing reverses doxorubicin resistance supports a functional link between TBX20 and drug efflux mechanisms.

However, several issues limit the impact of the study. First, the reliance on TBX20 overexpression without complementary loss-of-function experiments (e.g., TBX20 knockdown) weakens the causal interpretation of TBX20’s role in chemoresistance.For knockdown of ABCC1, at least two siRNAs could be appiled to rule out off-target effects. Second, the mechanistic connection between TBX20 and ABCC1 regulation is not fully established; it remains unclear whether TBX20 directly regulates ABCC1 transcription or acts indirectly through other pathways. Third, the assessment of mitophagy is based mainly on marker expression (LC3, P62, PINK1, and BNIP3), which may not be sufficient to conclusively demonstrate changes in mitophagic flux. Additional functional or dynamic assays would strengthen this conclusion. Finally, the absence of in vivo validation limits the translational significance of the findings.

In summary, the manuscript addresses an important question and provides suggestive evidence that TBX20 contributes to doxorubicin resistance in breast cancer cells. With additional mechanistic validation, inclusion of loss-of-function studies, and stronger mitophagy assays, the study could be significantly strengthened and make a more substantial contribution to the field.

Reviewer #2: This work highlighted the role of TBX20 in regulation ABCC1 and drug resistance in breast cancer, which is good. However, the manuscript has some concerns regarding consistency of data interpretation and consistency

Major comments

Concept

- The hypothesis of the author is based on that TBX20 induce drug resistance by inhibiting mitophagy. And this contradict the well-know and documented fact that mitophagy leads to drug resistance and , subsequently, its inhibition and targeting is a mechanism to decrease and overcome drug resistance, not induce it

- in the introduction author mentioned that “This study will delve into the role of TBX20 in breast cancer and its mechanism in promoting ADR resistance by inhibiting mitochondrial autophagy through ABCC1” , this sentence means that ABCC1 mediates the TBX20-mediated autophagy, which means that ABCC1 is directly involved in autophagy process itself. This role is never determined for ABCC1 before, no mechanistic validation done for this conclusion, neither by the author , nor by previous publication. The work of the authors linked between TBX20 and mitophagy, and between TBX20 and ABCC1 separately, abut not an axis TBX20/ABCC1/mitophagy.

Other major concerns

- -. In results part, author mentioned that TBX20 expression inhibit mitophagy and proofed that by increasing the LC3-II/LC3-I ratio in cells with enforced TBX20 expression compared to control, how come???. generally, in mitophagy the ratio of LC3-II/LC3-I increase, this means when you do a transduction that inhibit mitophagy it should leads to decrease, not increase of of LC3-II/LC3-I ratio. And in discussion author mentioned the ratio is decreased, so what written in results is not the same as in discussion.

- - the same in case of increasing the expression of P62, PINK1, and BNIP3 with compared to the NC and control, all of them are indicators of mitophagy, so if mitophagy inhibited, they should decrease, not increase as author mentioned in the text of results, which is not the same as mentioned in discussion. And the data in the figure 4 not matched with the text of results. in western blot figure, only p62 increase, while PINK1, and BNIP3 decrease. also LC3II is the one with 14 kd which showed decrease not increase in western image. Author need to reconsider data interpretation and drafting and avoid inconsistency

- To validate that TBX20 regulate drug resistance via inhibition of mitophagy, author compared IC50 for doxorubicin in cell with TBX20 expression vs cells with TBX20 expression+ mitophagy inducer. The correct to validate the role of TBX20 in mitophagy, you should compare ic50 in cells with mitophagy inducer vs. cells with TBX20+ mitophagy inducer, and you should test the mitophagy phenotype to confirm this. and again, the inhibition of mitophagy should reduce drug resistance, not the increase it.

- Same thing in case of validating the role of TBX20 in regulating drug resistance via ABCC1. author compared ic50 for doxorubicin in cell with TBX20 expression vs. cells with TBX20+ siABCC1. While you should compare IC50 in cells with siABCC1 vs. cells with TBX20+ siABCC1 to test the ability of TBX20 to restore the effect of siABCC1

- - data of IF need more explanation in text part of results and discussion. Also in legend of figure 4 of IF, author used the merged LC3 and TOM20 to conclude their colocalization. Which is not correct, because standard merged immunofluorescence (IF) is not sufficient on its own to validate colocalization because visual overlap (yellow color) can be misleading, often depending heavily on probe brightness and potentially masking true proximity or creating false positives. Colocalization requires other techniques not involved here, so you cant conclude they are colocalized.

Minor comment:

- the r value of sperman correlation is 0.243 revealed weak correlation between TBX20 and ABCC1

-in the second paragraph of results titled ‘Overexpression of TBX20 promotes doxorubicin resistance in breast cancer cell”:

* there is an error of figure citation, author cite figure in this paragraph as figure 1, while the representing figure is figure 2.

* the paragraph need rewriting to elaborate that the cell line are transfected, because the way it written in is confusing and look like the results are for naïve cell line. Elaboration is also required for CON and NC when they are written for the first time. And values representing the results (such as p value) should be mentioned in the text, not only in the legend of the figure

*treatment of cells with lentivirus for gene expression is called transduction, not transfection

- treatment with Amphotericin B and cccp not mentioned in methodology. Need explanation.

**********

6. PLOS authors have the option to publish the peer review history of their article (what does this mean?). If published, this will include your full peer review and any attached files.

If you choose “no”, your identity will remain anonymous but your review may still be made public.

**Do you want your identity to be public for this peer review?**  For information about this choice, including consent withdrawal, please see our Privacy Policy.

Reviewer #1: No

Reviewer #2: **Yes:**  Sohair Salem

**********

[NOTE: If reviewer comments were submitted as an attachment file, they will be attached to this email and accessible via the submission site. Please log into your account, locate the manuscript record, and check for the action link "View Attachments". If this link does not appear, there are no attachment files.]

To ensure your figures meet our technical requirements, please review our figure guidelines: https://journals.plos.org/plosone/s/figures

You may also use PLOS’s free figure tool, NAAS, to help you prepare publication quality figures: https://journals.plos.org/plosone/s/figures#loc-tools-for-figure-preparation.

NAAS will assess whether your figures meet our technical requirements by comparing each figure against our figure specifications.

Attachment Submitted filename: Review comments.docx

# Author comment: Author response to Decision Letter 1

**DOI:** 10.1371/journal.pone.0353376.r002

5 Mar 2026

Response to reviewers

Manuscript ID: PONE-D-25-64318

Title: TBX20 promotes doxorubicin resistance in breast cancer cells through enhanced ABCC1 expression and inhibition of mitophagy

Journal: PLOS ONE

Dear Dr. Peng Zhang and Reviewers,

Thank you for giving us the opportunity to revise our manuscript. We sincerely appreciate the time and effort that you and the reviewers have dedicated to providing insightful and constructive comments. We are grateful for the valuable feedback, which has helped us substantially improve the quality and clarity of our work.

We have carefully addressed all the points raised by the academic editor and reviewers. A copy of the revised manuscript with all changes highlighted using the “Track Changes” function in Microsoft Word has been uploaded as a separate file. The specific descriptions of the revisions are provided point-by-point below.

Reviewer #1: This manuscript investigates the role of TBX20 in doxorubicin resistance in breast cancer cells and proposes a mechanism involving enhanced ABCC1 expression and inhibition of mitophagy. The topic is clinically relevant, as chemoresistance remains a major challenge in breast cancer treatment and identifying novel regulatory factors may provide new therapeutic opportunities. The study presents several strengths. The authors combine bioinformatics analysis of TCGA datasets with in vitro functional experiments in two breast cancer cell lines, which strengthens the biological relevance of their findings. The use of multiple assays to assess proliferation, migration, drug resistance, and mitophagy provides a relatively comprehensive evaluation of TBX20 function. The observation that ABCC1 silencing reverses doxorubicin resistance supports a functional link between TBX20 and drug efflux mechanisms.

However, several issues limit the impact of the study. First, the reliance on TBX20 overexpression without complementary loss-of-function experiments (e.g., TBX20 knockdown) weakens the causal interpretation of TBX20’s role in chemoresistance.For knockdown of ABCC1, at least two siRNAs could be appiled to rule out off-target effects. Second, the mechanistic connection between TBX20 and ABCC1 regulation is not fully established; it remains unclear whether TBX20 directly regulates ABCC1 transcription or acts indirectly through other pathways. Third, the assessment of mitophagy is based mainly on marker expression (LC3, P62, PINK1, and BNIP3), which may not be sufficient to conclusively demonstrate changes in mitophagic flux. Additional functional or dynamic assays would strengthen this conclusion. Finally, the absence of in vivo validation limits the translational significance of the findings. In summary, the manuscript addresses an important question and provides suggestive evidence that TBX20 contributes to doxorubicin resistance in breast cancer cells. With additional mechanistic validation, inclusion of loss-of-function studies, and stronger mitophagy assays, the study could be significantly strengthened and make a more substantial contribution to the field.

Response:

Thank you for the reviewer's constructive comments. We have made important additions and revisions to the study and the manuscript in accordance with your suggestions. Our detailed responses are as follows:

①Supplementation of TBX20 loss-of-function experiments: The results indicate that silencing TBX20 leads to reduced ABCC1 expression and partially reverses doxorubicin resistance, thereby corroborating the findings from overexpression experiments and strengthening the causal role of TBX20 in chemotherapy resistance (see figures 3).

②Enhanced rigor of ABCC1 silencing experiments: We have included silencing efficiency data for ABCC1 using two independent siRNAs (see figures 6).

③Elucidation of the direct regulatory mechanism of TBX20 on ABCC1: Additional experiments have been conducted, including chromatin immunoprecipitation assays, which confirm that the TBX20 protein directly binds to the promoter region of the ABCC1 gene. Dual-luciferase reporter assays further demonstrate that TBX20 activates ABCC1 promoter activity (see figures 7).

④Strengthened functional validation of mitophagy: Our research is not only based on marker expression (LC3, P62, PINK1, and BNIP3) but also includes immunofluorescence to observe mitophagy levels using LC-3 and TOM20. This provides a visual assessment of mitophagy. Of course, further observations of mitophagy require additional techniques, such as electron microscopy and dynamic monitoring using LC-3 dual-fluorescence autophagy flux markers. However, given the current experimental conditions, these approaches are not feasible at this stage. We have acknowledged these limitations in the discussion section.

⑤Clarification regarding in vivo experiments: We fully understand and agree with the reviewer’s perspective on the importance of in vivo experiments for translational relevance. It is important to note that the focus of this study is to elucidate, for the first time at the cellular and molecular levels, a novel mechanism by which TBX20 mediates doxorubicin resistance in breast cancer through direct transcriptional regulation of ABCC1 and inhibition of mitophagy. The rigorous in vitro gain- and loss-of-function experiments, along with the mechanistic exploration conducted in this study, provide a solid theoretical foundation for TBX20 as a potential driver and therapeutic target in breast cancer chemoresistance. While the lack of in vivo experimental data is a limitation of the current study, we have explicitly acknowledged this point in the discussion section and emphasized its importance for future research.

Reviewer #2: This work highlighted the role of TBX20 in regulation ABCC1 and drug resistance in breast cancer, which is good. However, the manuscript has some concerns regarding consistency of data interpretation and consistency.

Major comments Concept

-The hypothesis of the author is based on that TBX20 induce drug resistance by inhibiting mitophagy. And this contradict the well-know and documented fact that mitophagy leads to drug resistance and , subsequently, its inhibition and targeting is a mechanism to decrease and overcome drug resistance, not induce it- in the introduction author mentioned that “This study will delve into the role of TBX20 in breast cancer and its mechanism in promoting ADR resistance by inhibiting mitochondrial autophagy through ABCC1” , this sentence means that ABCC1 mediates the TBX20-mediated autophagy, which means that ABCC1 is directly involved in autophagy process itself. This role is never determined for ABCC1 before, no mechanistic validation done for this conclusion, neither by the author, nor by previous publication. The work of the authors linked between TBX20 and mitophagy, and between TBX20 and ABCC1 separately, abut not an axis TBX20/ABCC1/mitophagy.

Response: We sincerely thank the reviewer for this insightful and critical comment, which allows us to clarify a key and novel aspect of our findings. We fully acknowledge the well-established paradigm that chemotherapy-induced mitophagy often acts as a cytoprotective mechanism, contributing to therapy resistance. Our data, however, appear to reveal a different, context-dependent role of mitophagy under the specific drive of TBX20.

①We agree that the observation—inhibition of mitophagy correlating with increased drug resistance—seems counterintuitive within the classic framework. To address this point, we have substantially revised the Discussion section. We now propose that TBX20 may not be modulating a “therapy-induced” mitophagy but rather suppressing basal or homeostatic mitophagic flux. This chronic suppression could lead to the accumulation of dysfunctional mitochondria, creating a state of sustained mitochondrial stress. In response, cells might activate robust compensatory survival pathways (such as the upregulation of the drug efflux pump ABCC1, as observed in our study) to adapt, ultimately resulting in a drug-resistant phenotype. This concept is supported by emerging literature showing that genetic or functional deficiencies in core mitophagy components (e.g., ULK1, ARL3) can promote tumor progression and therapy resistance in specific contexts [cited as ref. 21 & 22 in our revised manuscript]. Thus, our findings do not contradict but rather complement the existing knowledge by highlighting the “double-edged sword” nature of mitophagy in cancer. We have rewritten the relevant sections to present our hypothesis within this more nuanced and evolving theoretical framework.

We greatly appreciate the reviewer for pointing out this overstatement and lack of clarity in our original introduction. The reviewer is absolutely correct. We have no experimental evidence to support a direct role of ABCC1 in mediating mitophagy, and such a role has not been established in the literature. The sentence in question was an unintended and inaccurate synthesis of two separate observations.We have therefore made the following key revisions to the manuscript:

Introduction: The problematic sentence has been completely removed. The concluding paragraph of the introduction now states our aims more precisely and separately: “This study aims to... examining whether TBX20 promotes chemoresistance through the transcriptional upregulation of the ABCC1 and/or via the modulation of mitochondrial autophagy.”

Results & Discussion: Throughout the text, we now clearly present the upregulation of ABCC1 and the suppression of mitophagy as two distinct, important downstream consequences of TBX20 overexpression. We have replaced causal language (e.g., “through ABCC1”) with associative and correlative language (e.g., “is associated with,” “accompanied by”) to accurately reflect our data. We explicitly state in the Discussion that the mechanistic connection between ABCC1 upregulation and mitophagy suppression remains an open question and a valuable direction for future research.

②Regarding the construction of the “TBX20/ABCC1/Mitophagy” regulatory axis:

Clarifying the experimentally confirmed direct regulatory relationship: We emphasize the new experimental evidence, confirmed by chromatin immunoprecipitation and dual-luciferase reporter assays, that TBX20 directly transcriptionally upregulates ABCC1, and we clearly state that this is one of the core findings of this study.

Revising the functional role of ABCC1: We now define ABCC1 as a key downstream effector of TBX20, responsible for mediating TBX20-induced resistance to doxorubicin. This is strongly supported by our data (TBX20 upregulates ABCC1, and ABCC1 knockdown reverses TBX20-mediated resistance).

Clarifying the relationship between ABCC1 and mitophagy: We explicitly state that it remains unknown whether and how ABCC1 directly participates in the mitophagy process. Our data indicate that TBX20 upregulation of ABCC1 is associated with changes in mitophagy markers. However, the two may be parallel or co-downstream events, or they may be indirectly linked through mechanisms yet to be elucidated. We propose that ABCC1 may indirectly influence mitochondrial homeostasis—and thereby the status of mitophagy—through its drug efflux function, which alters the intracellular environment, or through other uncharacterized functions. We have revised the conclusion from a direct causal chain to a description of the observed association and have identified elucidating the precise mechanism as an important direction for future research. This limitation is also clearly noted in the discussion.

Other major concerns

- In results part, author mentioned that TBX20 expression inhibit mitophagy and proofed that by increasing the LC3-II/LC3-I ratio in cells with enforced TBX20 expression compared to control, how come? generally, in mitophagy the ratio of LC3-II/LC3-I increase, this means when you do a transduction that inhibit mitophagy it should leads to decrease, not increase of of LC3-II/LC3-I ratio. And in discussion author mentioned the ratio is decreased, so what written in results is not the same as in discussion.

- the same in case of increasing the expression of P62, PINK1, and BNIP3 with compared to the NC and control, all of them are indicators of mitophagy, so if mitophagy inhibited, they should decrease, not increase as author mentioned in the text of results, which is not the same as mentioned in discussion. And the data in the figure 4 not matched with the text of results. in western blot figure, only p62 increase, while PINK1, and BNIP3 decrease. Also LC3II is the one with 14 kd which showed decrease not increase in western image. Author need to reconsider data interpretation and drafting and avoid inconsistency.

Response: Thank you for the reviewer's meticulous and professional feedback. The inconsistencies you highlighted between the text description, the data figures, and the conclusions are of critical importance. We have conducted a thorough review and made corresponding revisions to the manuscript. Our detailed responses are as follows:

①We confirmed that the Western blot results in Figure 4B indeed show that, compared to the control group, the TBX20 overexpression group exhibited a decreased LC3-II/LC3-I ratio, as well as reduced expression of PINK1 and BNIP3 proteins. The phrase “significantly increased” in the original text was a serious clerical error. In the Results section, we have now corrected this to “significantly decreased” and “reduced” to ensure that the textual description perfectly matches the graphical data and supports the conclusion of “inhibited mitophagy.”

②Regarding a separate clarification on the P62 data: Your observation that the trend of P62 in the figure is inconsistent with that of PINK1/BNIP3 is very accurate. P62 is a multifunctional adaptor protein, and its accumulation is typically associated with the inhibition of autophagic flux (including mitophagy). To describe the data more precisely and avoid confusion, we have separately noted the trend of P62 in the revision, stating, "Notably, the expression of the autophagy receptor protein P62 was elevated." This formulation remains faithful to the Western blot results (which show an intensified P62 band) and aligns with the classic interpretation that "inhibition of autophagy/mitophagy leads to P62 accumulation." This resolves the apparent contradiction between the text and the figure and ensures consistency with the overall conclusions.

③Regarding consistency between the Results and Discussion sections: We have also cross-checked the Discussion section to ensure that all references to the results are consistent with the revised descriptions in the Results section. The discussion now clearly supports the finding that “TBX20 inhibits mitophagy,” thereby addressing the inconsistency you pointed out between different sections of the text.

④Regarding the figure citation numbering: We have corrected the figure numbers cited in the text to ensure they accurately correspond to the figure labels in the manuscript.

- To validate that TBX20 regulate drug resistance via inhibition of mitophagy, author compared IC50 for doxorubicin in cell with TBX20 expression vs cells with TBX20 expression+ mitophagy inducer. The correct to validate the role of TBX20 in mitophagy, you should compare ic50 in cells with mitophagy inducer vs. cells with TBX20+ mitophagy inducer, and you should test the mitophagy phenotype to confirm this. and again, the inhibition of mitophagy should reduce drug resistance, not the increase it.

- Same thing in case of validating the role of TBX20 in regulating drug resistance via ABCC1. author compared ic50 for doxorubicin in cell with TBX20 expression vs. cells with TBX20+ siABCC1. While you should compare IC50 in cells with siABCC1 vs. cells with TBX20+ siABCC1 to test the ability of TBX20 to restore the effect of siABCC1.

Response:

We have performed the following additional experiments as suggested:

Comparative IC50 Analysis: We compared the doxorubicin sensitivity between cells treated with CCCP alone and cells with TBX20 + CCCP. The results, now included in the revised manuscript (Fig. 4D), show that while CCCP treatment alone significantly sensitizes control cells to doxorubicin, this sensitizing effect is markedly blunted in TBX20-overexpressing cells. The IC50 of

Attachment Submitted filename: Response to reviewers.docx

# Peer review: Decision Letter 1

**DOI:** 10.1371/journal.pone.0353376.r003

**Authors:** Peng Zhang

29 Mar 2026

PONE-D-25-64318R1

TBX20 promotes doxorubicin resistance in breast cancer cells through enhanced ABCC1 expression and inhibition of mitophagy

PLOS One

Dear Dr. Mi,

Thank you for submitting your manuscript to PLOS ONE. After careful consideration, we feel that it has merit but does not fully meet PLOS ONE’s publication criteria as it currently stands. Therefore, we invite you to submit a revised version of the manuscript that addresses the points raised during the review process.

Please submit your revised manuscript by May 13 2026 11:59PM. If you will need more time than this to complete your revisions, please reply to this message or contact the journal office at plosone@plos.org. When you're ready to submit your revision, log on to https://www.editorialmanager.com/pone/ and select the 'Submissions Needing Revision' folder to locate your manuscript file.

Please include the following items when submitting your revised manuscript:

- A letter that responds to each point raised by the academic editor and reviewer(s). You should upload this letter as a separate file labeled 'Response to Reviewers'.
- A marked-up copy of your manuscript that highlights changes made to the original version. You should upload this as a separate file labeled 'Revised Manuscript with Track Changes'.
- An unmarked version of your revised paper without tracked changes. You should upload this as a separate file labeled 'Manuscript'.

If you would like to make changes to your financial disclosure, please include your updated statement in your cover letter. Guidelines for resubmitting your figure files are available below the reviewer comments at the end of this letter.

If applicable, we recommend that you deposit your laboratory protocols in protocols.io to enhance the reproducibility of your results. Protocols.io assigns your protocol its own identifier (DOI) so that it can be cited independently in the future. For instructions see: https://journals.plos.org/plosone/s/submission-guidelines#loc-laboratory-protocols. Additionally, PLOS ONE offers an option for publishing peer-reviewed Lab Protocol articles, which describe protocols hosted on protocols.io. Read more information on sharing protocols at https://plos.org/protocols?utm_medium=editorial-email&utm_source=authorletters&utm_campaign=protocols.

As the corresponding author, your ORCID iD is verified in the submission system and will appear in the published article. PLOS supports the use of ORCID, and we encourage all coauthors to register for an ORCID iD and use it as well. Please encourage your coauthors to verify their ORCID iD within the submission system before final acceptance, as unverified ORCID iDs will not appear in the published article. *Only*  the individual author can complete the verification step; PLOS staff *cannot*  verify ORCID iDs on behalf of authors.

We look forward to receiving your revised manuscript.

Kind regards,

Peng Zhang, Ph.D.

Academic Editor

PLOS One

Journal Requirements:

If the reviewer comments include a recommendation to cite specific previously published works, please review and evaluate these publications to determine whether they are relevant and should be cited. There is no requirement to cite these works unless the editor has indicated otherwise.

[Note: HTML markup is below. Please do not edit.]

Reviewers' comments:

Reviewer's Responses to Questions

**Comments to the Author**

1. If the authors have adequately addressed your comments raised in a previous round of review and you feel that this manuscript is now acceptable for publication, you may indicate that here to bypass the “Comments to the Author” section, enter your conflict of interest statement in the “Confidential to Editor” section, and submit your "Accept" recommendation.

Reviewer #1: All comments have been addressed

Reviewer #2: (No Response)

**********

2. Is the manuscript technically sound, and do the data support the conclusions?

The manuscript must describe a technically sound piece of scientific research with data that supports the conclusions. Experiments must have been conducted rigorously, with appropriate controls, replication, and sample sizes. The conclusions must be drawn appropriately based on the data presented.

Reviewer #1: Partly

Reviewer #2: Partly

**********

3. Has the statistical analysis been performed appropriately and rigorously?

Reviewer #1: Yes

Reviewer #2: Yes

**********

4. Have the authors made all data underlying the findings in their manuscript fully available?

The PLOS Data policy requires authors to make all data underlying the findings described in their manuscript fully available without restriction, with rare exception (please refer to the Data Availability Statement in the manuscript PDF file). The data should be provided as part of the manuscript or its supporting information, or deposited to a public repository. For example, in addition to summary statistics, the data points behind means, medians and variance measures should be available. If there are restrictions on publicly sharing data—e.g. participant privacy or use of data from a third party—those must be specified.

Reviewer #1: Yes

Reviewer #2: Yes

**********

5. Is the manuscript presented in an intelligible fashion and written in standard English?

PLOS ONE does not copyedit accepted manuscripts, so the language in submitted articles must be clear, correct, and unambiguous. Any typographical or grammatical errors should be corrected at revision, so please note any specific errors here.

Reviewer #1: Yes

Reviewer #2: Yes

**********

6. Review Comments to the Author

Please use the space provided to explain your answers to the questions above. You may also include additional comments for the author, including concerns about dual publication, research ethics, or publication ethics. (Please upload your review as an attachment if it exceeds 20,000 characters)

Reviewer #1: The authors have addressed the concern by using TBX20 and ABCC1 siRNAs to verify the functional link between TBX20 and ABCC1 in drug resistance. However, additional Western blot data should be provided to determine whether knockdown of TBX20 or ABCC1 affects mitophagy-related markers (e.g., LC3-II,PINK1,et al). This is particularly important if the authors maintain the claim that TBX20 promotes doxorubicin resistance in breast cancer cells through enhanced ABCC1 expression and inhibition of mitophagy.

In addition, could the authors clarify why LC3-II levels are elevated under basal conditions (Figure 5B)? This observation appears inconsistent with the confocal images (Figure 5A), which show no detectable LC3 puncta at baseline. Typically, increased LC3-II levels correlate with the formation of LC3 puncta in immunofluorescence staining.

Reviewer #2: The revised manuscript contains good explanations that made the manuscript clearer and more consistent. Authors carried out most of required modifications, however, there is an important experiment they responded by doing it, but it is not included in the manuscript:

- Author responded to my initial comment about role of TBX20 in modifying doxorubicin sensitivity via ABCC1 and mitophagy that they did a rescue experiment and they compared the doxorubicin sensitivity between cells treated with CCCP alone (not TBX20 alone) and cells with TBX20 + CCCP; and compared siABCC1 alone with TBX20 overexpression+ siABCC1 in figure 5 and 6, however the figures and text don’t contain these new results, they are the same as the data in the original manuscript. In addition, figure 5 is A, B, and C, while its legend is A, B, C, and D. check out consistency between figure body and legend.

- The revised manuscript doesn’t contain figure 1 (only the figure body not exist, you may forgot to submit it)

**********

7. PLOS authors have the option to publish the peer review history of their article (what does this mean?). If published, this will include your full peer review and any attached files.

If you choose “no”, your identity will remain anonymous but your review may still be made public.

**Do you want your identity to be public for this peer review?**  For information about this choice, including consent withdrawal, please see our Privacy Policy.

Reviewer #1: No

Reviewer #2: No

**********

[NOTE: If reviewer comments were submitted as an attachment file, they will be attached to this email and accessible via the submission site. Please log into your account, locate the manuscript record, and check for the action link "View Attachments". If this link does not appear, there are no attachment files.]

To ensure your figures meet our technical requirements, please review our figure guidelines: https://journals.plos.org/plosone/s/figures

You may also use PLOS’s free figure tool, NAAS, to help you prepare publication quality figures: https://journals.plos.org/plosone/s/figures#loc-tools-for-figure-preparation.

NAAS will assess whether your figures meet our technical requirements by comparing each figure against our figure specifications.

Attachment Submitted filename: new review comments.docx

# Author comment: Author response to Decision Letter 2

**DOI:** 10.1371/journal.pone.0353376.r004

8 Jun 2026

Reviewer #1: The authors have addressed the concern by using TBX20 and ABCC1 siRNAs to verify the functional link between TBX20 and ABCC1 in drug resistance. However, additional Western blot data should be provided to determine whether knockdown of TBX20 or ABCC1 affects mitophagy-related markers (e.g., LC3-II, PINK1, et al). This is particularly important if the authors maintain the claim that TBX20 promotes doxorubicin resistance in breast cancer cells through enhanced ABCC1 expression and inhibition of mitophagy.

Response: We sincerely thank Reviewer for the insightful comment regarding the need for additional mechanistic evidence linking TBX20, ABCC1, and mitophagy. We agree that providing Western blot data on mitophagy markers following gene knockdown is essential to substantiate our claim. In the revised manuscript, we have supplemented the Western blot results regarding autophagy-related markers to Figure 7 and the corresponding text has been added to the Results section. The detailed experimental results are described below:

In both MCF-7 and MDA-MB-231 cells, qPCR analysis revealed that the mRNA expression levels of ABCC1and TBX20were significantly downregulated in the si-TBX20 and si-ABCC1 groups compared with the si-NC group (P< 0.001). Notably, TBX20mRNA expression was significantly upregulated in the si-ABCC1 group (P< 0.001) (Fig. 7A and 7D). Consistent with these findings, Western blot assays showed that the protein levels of ABCC1 and TBX20 were markedly decreased in both the si-TBX20 and si-ABCC1 groups relative to the si-NC group (P< 0.001); however, there was no statistically significant difference in TBX20 protein expression in the si-ABCC1 group (Fig. 7B and 7E). Furthermore, regarding autophagy-related proteins, the ratio of LC3-II/LC3-I was significantly increased, while the expression of p62 was notably reduced in both knockdown groups (P< 0.001). Concurrently, the mitophagy-related proteins PINK1 and BNIP3 were also significantly upregulated (P< 0.001) (Fig. 7C and 7F).

Fig. 7 Silencing of ABCC1 or TBX20 alters the expression of autophagy-related proteins. (A): qPCR analysis of ABCC1and TBX20 mRNA expression. (B) Western blot analysis of ABCC1 and TBX20 protein levels. (C) Western blot analysis of autophagy markers (LC3-I/II, p62) and mitophagy-associated proteins (PINK1, BNIP3). (D) Relative mRNA expression levels of ABCC1and TBX20 by qPCR. (E) Protein expression levels of ABCC1 and TBX20 by Western blotting. (F) Protein levels analysis of LC3-I/II, p62, PINK1, and BNIP3 by Western blotting. Data are presented as mean ± SD (n=3); ***P< 0.001 compared with the si-NC group, ns: no significant difference.

In addition, could the authors clarify why LC3-II levels are elevated under basal conditions (Figure 5B)? This observation appears inconsistent with the confocal images (Figure 5A), which show no detectable LC3 puncta at baseline. Typically, increased LC3-II levels correlate with the formation of LC3 puncta in immunofluorescence staining.

Response: We appreciate the reviewer’s careful observation. We would like to clarify that LC3-II levels are not elevated relative to the control groups; rather, they are significantly decreased in TBX20‑overexpressing cells compared with the NC and CON groups (Fig. 5B). The apparent confusion likely stems from the direction of comparison. In our study, TBX20 overexpression suppresses autophagosome formation, leading to reduced LC3‑II levels, which is consistent with the markedly diminished LC3 puncta observed in confocal imaging (Fig. 5A). Therefore, the Western blot and immunofluorescence data are fully aligned: both indicate that TBX20 overexpression reduces LC3‑II and LC3 puncta relative to baseline controls, rather than increasing them.

Reviewer #2: Author responded to my initial comment about role of TBX20 in modifying doxorubicin sensitivity via ABCC1 and mitophagy that they did a rescue experiment and they compared the doxorubicin sensitivity between cells treated with CCCP alone (not TBX20 alone) and cells with TBX20 + CCCP; and compared siABCC1 alone with TBX20 overexpression+ siABCC1 in figure 5 and 6, however the figures and text don’t contain these new results, they are the same as the data in the original manuscript. In addition, figure 5 is A, B, and C, while its legend is A, B, C, and D. check out consistency between figure body and legend.

- The revised manuscript doesn’t contain figure 1 (only the figure body not exist, you may forgot to submit it)

Response: We sincerely thank the reviewer for the insightful comments and suggestions. In the revised manuscript, we have made the following corrections and supplements regarding Figures 5 and 6:

Correction of Figure 5: Due to an oversight in the previous version, the textual description and figure legend of Figure 5 did not match the final figure content. We have now updated both the results description and the legend for Figure 5 to ensure accuracy and consistency.

Supplementation of Figure 6: We have included the rescue experiment data in Figure 6E, demonstrating that knockdown of ABCC1 reverses the doxorubicin resistance induced by TBX20 overexpression. The corresponding image files have also been uploaded to the submission system.

we have supplemented the Western blot results regarding autophagy-related markers to Figure 7 and the corresponding text has been added to the Results section.

We apologize for the omission. The missing Figure 1 has now been uploaded to the system. The details of the revisions are provided below.

Results Section

For Figure 5: In confocal microscopy observation, the localization of LC3 (green) and TOM20 (red) in MCF-7 and MDA-MB-231 cells with TBX20 overexpression showed changes in the spatial pattern and fluorescence intensity compared to the NC and CON groups (P < 0.001), the TBX20 group exhibited markedly diminished LC3 puncta and reduced green fluorescence intensity (Fig. 5A). The Western blot results showed that the LC3-II/LC3-I ratio in MCF-7 and MDA-MB-231 cells with TBX20 overexpression significantly decreased compared with the NC group, and the expression of PINK1 and BNIP3 was also reduced. Notably, the expression of P62 was elevated (P < 0.001) (Fig. 5B). The analysis of cell inhibition rate treated with CCCP showed that the IC50 values of the TBX20+CCCP group were significantly lower than those of the TBX20 group in both cell lines, suggesting that TBX20 may affect cell sensitivity to drugs by regulating autophagy (Fig. 5C).

Fig. 5 Overexpression of TBX20 inhibits mitophagy in breast cancer cells. (A) Immunofluorescence staining of mitochondria (TOM20, red), autophagosomes (LC3, green), and nuclei (DAPI, blue) in MCF-7 and MDA-MB-231 cells. Representative merged images show the spatial distribution of LC3 and TOM20 signals. Yellow regions in the merged channels visually indicate areas of signal overlap, which were qualitatively assessed. Representative images were acquired at 1500× magnification; insets show enlarged regions at 5000X magnification. Scale bars: 20 μm (1500×) and 5 μm (5000×). (B) Western blot analysis of mitophagy-related proteins (LC3-II/I, PINK1, BNIP3, and p62) in MCF-7 and MDA-MB-231 cells transfected with TBX20-overexpressing or control vectors. (C) CCK-8 assay measuring IC50 values of doxorubicin in MCF-7 and MDA-MB-231 cells following treatment with the mitophagy inducer CCCP (10 μM, 24 h). IC50 values were calculated using nonlinear regression analysis. Data are presented as mean ± SD (n=3); *** P <0.001, TBX20 group vs NC group. CON: Untreated control cells. NC: Negative control; TBX20: Overexpression of TBX20; ADR: Adriamycin; IC50: Half maximal inhibitory concentration.

For Figure 6: In MCF-7 and MDA-MB-231 cells, TBX20 treatment significantly upregulated ABCC1 mRNA expression, with a notable increase in ABCC1 mRNA in the TBX20-treated group compared to the NC groups (P < 0.001) (Fig. 6A). Western blot analysis showed that ABCC1 protein expression was significantly elevated in the TBX20 group, with higher levels of ABCC1 protein expression compared to the NC groups (P < 0.001) (Fig. 6B). RT-PCR and western blot analyses were conducted to evaluate the silencing efficiency of two ABCC1-targeting siRNAs (si-ABCC1#1 and si-ABCC1#2). Both siRNAs effectively knocked down ABCC1 expression, as evidenced by decreased mRNA (Fig. 6C) and protein (Fig. 6D) levels. Dose-response curve analysis demonstrated that the inhibition curve for the TBX20 + si-NC group was positioned lowest, indicating the poorest sensitivity to doxorubicin and the highest IC50 value. In contrast, the TBX20 + si-ABCC1 #1 and TBX20 + si-ABCC1 #2 groups exhibited significantly higher inhibition rates and markedly reduced IC50 values compared to the control group. Furthermore, both si-ABCC1 #1 and #2 groups alone also displayed enhanced drug sensitivity and lower IC50, suggesting that silencing ABCC1effectively potentiates the cytotoxic effects of doxorubicin (Fig. 6E).

Fig. 6 TBX20 enhances doxorubicin resistance in breast cancer cells by increasing ABCC1 expression. (A) Relative mRNA expression of ABCC1 in MCF-7 and MDA-MB-231 cells transfected with TBX20-overexpressing or control vectors, determined by qPCR. GAPDH was used as an internal control. (B) Western blot analysis confirming ABCC1 protein expression in MCF-7 and MDA-MB-231 cells following TBX20 overexpression. (C) Relative mRNA expression of ABCC1 in MCF-7 and MDA-MB-231 cells transfected with si-ABCC1, as determined by qPCR. (D) Western blot analysis confirming ABCC1 protein expression in MCF-7 and MDA-MB-231 cells. GAPDH was used as a loading control. (E) CCK-8 assay measuring IC50 values of doxorubicin in MCF-7 and MDA-MB-231 cells after co-transduction with TBX20 and si-ABCC1. IC50 values were determined via nonlinear regression analysis. Data are presented as mean ± SD (n=3); *** P <0.001. TBX20: TBX20 overexpression with negative control siRNA; TBX20 + si-ABCC1: TBX20 overexpression with

ABCC1-siRNA co-transduction.

For Figure 7: In both MCF-7 and MDA-MB-231 cells, qPCR analysis revealed that the mRNA expression levels of ABCC1and TBX20were significantly downregulated in the si-TBX20 and si-ABCC1 groups compared with the si-NC group (P< 0.001) (Fig. 7A and 7D). Consistent with these findings, Western blot assays showed that the protein levels of ABCC1 and TBX20 were markedly decreased in both the si-TBX20 and si-ABCC1 groups relative to the si-NC group (P< 0.001) (Fig. 7B and 7E). Furthermore, regarding autophagy-related proteins, the ratio of LC3-II/LC3-I was significantly increased, while the expression of p62 was notably reduced in both knockdown groups (P< 0.001). Concurrently, the mitophagy-related proteins PINK1 and BNIP3 were also significantly upregulated (P< 0.001) (Fig. 7C and 7F).

Fig. 7 Silencing of ABCC1 or TBX20 alters the expression of autophagy-related proteins. (A): qPCR analysis of ABCC1and TBX20 mRNA expression. (B) Western blot analysis of ABCC1 and TBX20 protein levels. (C) Western blot analysis of autophagy markers (LC3-I/II, p62) and mitophagy-associated proteins (PINK1, BNIP3). (D) Relative mRNA expression levels of ABCC1and TBX20 by qPCR. (E) Protein expression levels of ABCC1 and TBX20 by Western blotting. (F) Protein levels analysis of LC3-I/II, p62, PINK1, and BNIP3 by Western blotting. Data are presented as mean ± SD (n=3); ***P< 0.001 compared with the si-NC group, ns: no significant difference. The si-TBX20 group refers to cells transfected with the TBX20 siRNA duplex si-TBX20#1, and the si-ABCC1 group refers to cells transfected with the ABCC1siRNA duplex si-ABCC1#1. The si-NC group indicates cells transfected with a non-targeting negative control siRNA

Attachment Submitted filename: Response_to_Reviewers_auresp_2.docx

# Peer review: Decision Letter 2

**DOI:** 10.1371/journal.pone.0353376.r005

**Authors:** Peng Zhang

24 Jun 2026

<p>TBX20 promotes doxorubicin resistance in breast cancer cells through enhanced ABCC1 expression and inhibition of mitophagy

PONE-D-25-64318R2

Dear Dr. Mi,

We’re pleased to inform you that your manuscript has been judged scientifically suitable for publication and will be formally accepted for publication once it meets all outstanding technical requirements.

Within one week, you’ll receive an e-mail detailing the required amendments. When these have been addressed, you’ll receive a formal acceptance letter and your manuscript will be scheduled for publication.

An invoice will be generated when your article is formally accepted. Please note, if your institution has a publishing partnership with PLOS and your article meets the relevant criteria, all or part of your publication costs will be covered. Please make sure your user information is up-to-date by logging into Editorial Manager at Editorial Manager® and clicking the ‘Update My Information' link at the top of the page. For questions related to billing, please contact billing support.

If your institution or institutions have a press office, please notify them about your upcoming paper to help maximize its impact. If they’ll be preparing press materials, please inform our press team as soon as possible -- no later than 48 hours after receiving the formal acceptance. Your manuscript will remain under strict press embargo until 2 pm Eastern Time on the date of publication. For more information, please contact onepress@plos.org.

Kind regards,

Peng Zhang, Ph.D.

Academic Editor

PLOS One

Additional Editor Comments (optional):

Reviewers' comments:

Reviewer's Responses to Questions

**Comments to the Author**

1. If the authors have adequately addressed your comments raised in a previous round of review and you feel that this manuscript is now acceptable for publication, you may indicate that here to bypass the “Comments to the Author” section, enter your conflict of interest statement in the “Confidential to Editor” section, and submit your "Accept" recommendation.

Reviewer #1: (No Response)

Reviewer #2: All comments have been addressed

**********

2. Is the manuscript technically sound, and do the data support the conclusions?

The manuscript must describe a technically sound piece of scientific research with data that supports the conclusions. Experiments must have been conducted rigorously, with appropriate controls, replication, and sample sizes. The conclusions must be drawn appropriately based on the data presented.

Reviewer #1: Yes

Reviewer #2: Yes

**********

3. Has the statistical analysis been performed appropriately and rigorously?

Reviewer #1: Yes

Reviewer #2: Yes

**********

4. Have the authors made all data underlying the findings in their manuscript fully available?

The PLOS Data policy requires authors to make all data underlying the findings described in their manuscript fully available without restriction, with rare exception (please refer to the Data Availability Statement in the manuscript PDF file). The data should be provided as part of the manuscript or its supporting information, or deposited to a public repository. For example, in addition to summary statistics, the data points behind means, medians and variance measures should be available. If there are restrictions on publicly sharing data—e.g. participant privacy or use of data from a third party—those must be specified.

Reviewer #1: Yes

Reviewer #2: Yes

**********

5. Is the manuscript presented in an intelligible fashion and written in standard English?

PLOS ONE does not copyedit accepted manuscripts, so the language in submitted articles must be clear, correct, and unambiguous. Any typographical or grammatical errors should be corrected at revision, so please note any specific errors here.

Reviewer #1: Yes

Reviewer #2: Yes

**********

6. Review Comments to the Author

Please use the space provided to explain your answers to the questions above. You may also include additional comments for the author, including concerns about dual publication, research ethics, or publication ethics. (Please upload your review as an attachment if it exceeds 20,000 characters)

Reviewer #1: (No Response)

Reviewer #2: (No Response)

**********

7. PLOS authors have the option to publish the peer review history of their article (what does this mean?). If published, this will include your full peer review and any attached files.

If you choose “no”, your identity will remain anonymous but your review may still be made public.

**Do you want your identity to be public for this peer review?**  For information about this choice, including consent withdrawal, please see our Privacy Policy.

Reviewer #1: **Yes:**  Lei Sun

Reviewer #2: No

**********

# Editor report: Acceptance letter

**DOI:** 10.1371/journal.pone.0353376.r006

**Authors:** Peng Zhang

PONE-D-25-64318R2

PLOS One

Dear Dr. Mi,

I'm pleased to inform you that your manuscript has been deemed suitable for publication in PLOS One. Congratulations! Your manuscript is now being handed over to our production team.

At this stage, our production department will prepare your paper for publication. This includes ensuring the following:

* All references, tables, and figures are properly cited

* All relevant supporting information is included in the manuscript submission,

* There are no issues that prevent the paper from being properly typeset

You will receive further instructions from the production team, including instructions on how to review your proof when it is ready. Please keep in mind that we are working through a large volume of accepted articles, so please give us a few days to review your paper and let you know the next and final steps.

Lastly, if your institution or institutions have a press office, please let them know about your upcoming paper now to help maximize its impact. If they'll be preparing press materials, please inform our press team within the next 48 hours. Your manuscript will remain under strict press embargo until 2 pm Eastern Time on the date of publication. For more information, please contact onepress@plos.org.

You will receive an invoice from PLOS for your publication fee after your manuscript has reached the completed accept phase. If you receive an email requesting payment before acceptance or for any other service, this may be a phishing scheme. Learn how to identify phishing emails and protect your accounts at https://explore.plos.org/phishing.

If we can help with anything else, please email us at customercare@plos.org.

Thank you for submitting your work to PLOS ONE and supporting open access.

Kind regards,

PLOS ONE Editorial Office Staff

on behalf of

Prof. Peng Zhang

Academic Editor

PLOS One
